# CMPK2 is a host restriction factor that inhibits infection of multiple coronaviruses in a cell-intrinsic manner

**DOI:** 10.1371/journal.pbio.3002039

**Published:** 2023-03-17

**Authors:** Mingjun Zhu, Jiahuang Lv, Wei Wang, Rongli Guo, Chunyan Zhong, Avan Antia, Qiru Zeng, Jizong Li, Qingtao Liu, Jinzhu Zhou, Xuejiao Zhu, Baochao Fan, Siyuan Ding, Bin Li

**Affiliations:** 1 Key Laboratory of Veterinary Biological Engineering and Technology Ministry of Agriculture, Institute of Veterinary Medicine, Jiangsu Academy of Agricultural Sciences, Nanjing, Jiangsu, China; 2 Jiangsu Key Laboratory for Food Quality and Safety-State Key Laboratory Cultivation Base of Ministry of Science and Technology, Institute of Veterinary Medicine, Jiangsu Academy of Agricultural Sciences; Nanjing, Jiangsu, China; 3 Jiangsu Co-innovation Center for Prevention and Control of Important Animal Infectious Diseases and Zoonoses, Yangzhou University, Yangzhou, Jiangsu, China; 4 College of Animal Science, Tibet Agricultural and Animal Husbandry University College of Veterinary Medicine, Nyingchi, Tibet, China; 5 Biological Engineering Department, Southwest Guizhou Vocational and Technical College for Nationalities, Xingyi, China; 6 Department of Molecular Microbiology, Washington University School of Medicine, St. Louis, Missouri, United States of America; Ulm University Medical Center, GERMANY

## Abstract

Coronaviruses (CoVs) comprise a group of important human and animal pathogens. Despite extensive research in the past 3 years, the host innate immune defense mechanisms against CoVs remain incompletely understood, limiting the development of effective antivirals and non-antibody-based therapeutics. Here, we performed an integrated transcriptomic analysis of porcine jejunal epithelial cells infected with porcine epidemic diarrhea virus (PEDV) and identified cytidine/uridine monophosphate kinase 2 (CMPK2) as a potential host restriction factor. CMPK2 exhibited modest antiviral activity against PEDV infection in multiple cell types. CMPK2 transcription was regulated by interferon-dependent and interferon regulatory factor 1 (IRF1)-dependent pathways post-PEDV infection. We demonstrated that 3′-deoxy-3′,4′-didehydro-cytidine triphosphate (ddhCTP) catalysis by Viperin, another interferon-stimulated protein, was essential for CMPK2’s antiviral activity. Both the classical catalytic domain and the newly identified antiviral key domain of CMPK2 played crucial roles in this process. Together, CMPK2, viperin, and ddhCTP suppressed the replication of several other CoVs of different genera through inhibition of the RNA-dependent RNA polymerase activities. Our results revealed a previously unknown function of CMPK2 as a restriction factor for CoVs, implying that CMPK2 might be an alternative target of interfering with the viral polymerase activity.

## Abstract 2

This study shows that cytidine monophosphate kinase 2 (CMPK2), whose induction is at least partially independent of interferons, broadly inhibits coronavirus replication; the progression of viral RNA-dependent RNA polymerase is stalled by ddhCTP synthesized by CMPK2 and Viperin.

## Introduction

Coronaviruses (CoVs) are important pathogens that pose serious threats to human and animal health. Research on CoVs has largely been lagging, with sporadic attention during the outbreaks of Severe Acute Respiratory Syndrome Coronavirus (SARS-CoV) and Middle East Respiratory Syndrome Coronavirus (MERS-CoV) [1]. The ongoing SARS-CoV-2 pandemic has infected more than 600 million and caused the death of more than 6.6 million people worldwide [2]. Even in the presence of effective vaccines of various platforms, there is an urgent need to understand SARS-CoV-2 biology and develop specific and broad-spectrum antiviral inhibitors.

CoVs are positive-sense single-stranded RNA viruses and possess the largest genome among RNA viruses, which encodes the standard set of 4 structural proteins: spike (S), envelope (E), membrane (M), and nucleocapsid (N) and 16 (mostly) functional nonstructural proteins (nsp1-nsp16) [3–5]. These proteins are relevant for CoV replication and pathogenesis in vivo, by either interfering with the host immune response or directly assisting steps of the viral replication cycle. Nsp12, a major component of RNA-dependent RNA polymerase (RdRp), is the powerhouse of CoV replication [6–8]. Although RdRp is an obvious target of antiviral therapeutics development, Gilead’s remdesivir and Merck’s molnupiravir represent the only 2 FDA-authorized antiviral drugs [9,10]. In addition, there is significant concern that monotherapy would rapidly result in the emergence of resistance [11,12].

CoVs consist of 4 genera: α, β, γ, and δ, represented by porcine epidemic diarrhea virus (PEDV, α), SARS-CoV-2 (β), infectious bronchitis virus (IBV, γ), and porcine delta-coronavirus (PDCoV, δ), which are common pathogens for birds and mammals [13,14]. Here, we use PEDV as a model virological system to probe the molecular mechanisms of host defense against CoV infection, which will pave the path for the development of effective broad-spectrum anti-CoV pharmacological inhibitors.

Cytidine/uridine monophosphate kinase 2 (CMPK2, also known as TYKi/TMPK2) is a nucleoside monophosphate kinase and is implicated in the synthesis of mitochondrial DNA (mtDNA) and maintaining intracellular UTP/CTP [15,16]. CMPK2 was originally identified as one of the interferon-stimulated genes (ISGs) induced by type I interferon (IFN-I) and was confirmed for its ability to suppress human immunodeficiency virus (HIV), spring viremia of carp virus (SVCV) as well as dengue virus (DENV) replication [17–19]. However, many CoVs are propagated in Vero cells that naturally lack genes encoding IFN-I, and multiple proteins of CoVs suppress IFN production via various mechanisms [20–22]. Therefore, the relationship between CoVs, CMPK2, and IFN-I still needs further clarification. Furthermore, in the human genome, CMPK2 was found adjacent to and inverted with respect to the RSAD2 (encoding Viperin, a well-characterized ISG with antiviral activity; [23]). Intriguingly, CMPK2 and Viperin are often coexpressed after IFN-I stimulation or viral infection. CMPK2 catalyzes the phosphorylation of CDP to produce CTP, and Viperin mediates the conversion of CTP to 3′-deoxy-3′,4′-didehydro-cytidine triphosphate (ddhCTP) [24,25], resulting in an approximately 4-fold increase in ddhCTP production [24,26]. More evidence showed that ddhCTP acts as a chain terminator for RdRp and directly prevents the replication of many viruses including Zika virus and West Nile virus [25,27]. CMPK2 was identified in a recent ISG screen to potentially inhibit SARS-CoV-2 replication [28]. Nevertheless, whether CMPK2 broadly inhibits CoV replication and the underlying molecular mechanisms are still not clear.

Here, we employed a transcriptome sequencing approach to identify host factors that confer protection against CoV infection and demonstrated that CMPK2 was both up-regulated in vitro and in vivo following PEDV infection. Although multiple proteins of CoVs including PEDV restrain antiviral innate immune response via various mechanisms [20,21], up-regulation of CMPK2 expression occurred in host cells at least partially independent of the IFN-I pathway. Mechanistically, we found that via the catalytic residues and an antiviral key domain (AKD), CMPK2 inhibits PEDV replication through increasing ddhCTP production and inhibiting the RdRp activities. Collectively, our results implicate CMPK2 as an effective host defense factor during virus infection and provided a novel cellular target for controlling CoV infections.

## Results

### PEDV infection up-regulates the expression of CMPK2

To identify potential host antiviral factors against CoVs, we infected neonatal porcine jejunal columnar epithelial IPEC-J2 cells with PEDV (strain AH2012/12) at MOI (multiplicity of infection) of 1 and examined the global transcriptomics at 20 hours post-infection (hpi). We found that multiple ISGs were up-regulated during PEDV infection, such as MX1, ISG15, and RSAD2 (S1A Fig). The innate immune system provides the first line of defense against foreign pathogens and mitochondria functions as a central hub in the process [29]. Multiple innate immune pathways are subjected to mitochondrial regulation [30]. Therefore, we performed an integrated analysis of mitochondrial related genes in our transcriptome data. We found that cytidine monophosphate kinase 2 (encoded by *CMPK2*, a rate-limiting enzyme of mtDNA synthesis) was highly and specifically up-regulated than other mitochondrial genes (S1B Fig).

**Fig 1:**
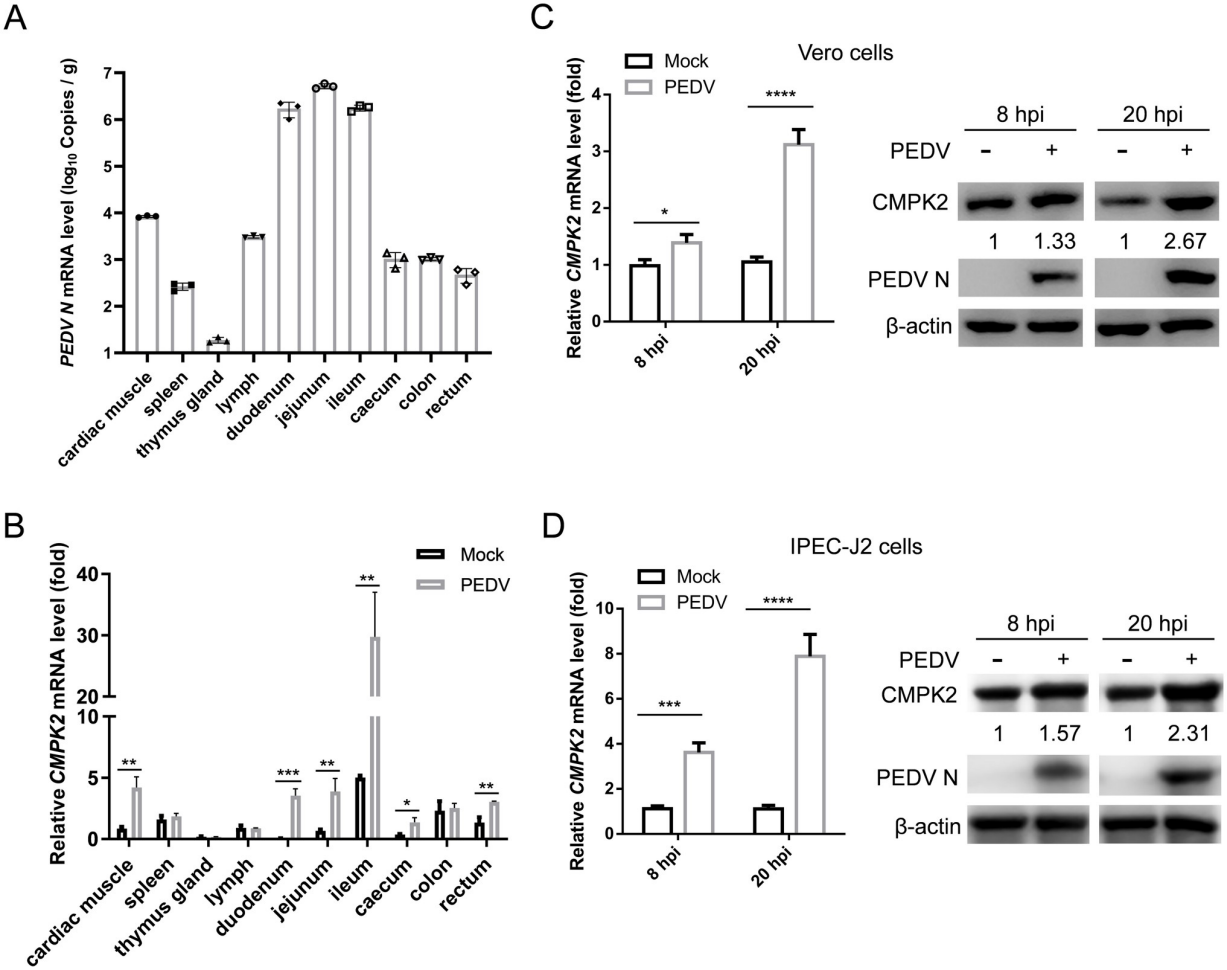
PEDV infection up-regulates CMPK2 expression. **(A and B)** At 7 dpi with PEDV, viral copy numbers in per g of tissue (**A**) and relative transcriptional levels of CMPK2 (**B**) in piglet tissues (*N* = 3) were, respectively, analyzed with a standard curve and qRT-PCR. **(C and D)** Vero and IPEC-J2 cells were infected with PEDV (MOI = 1) and harvested at 8 and 20 hpi, respectively. The expression of CMPK2 were detected by qRT-PCR (left) and western blot (right). Data are means ± SD of triplicate samples; statistical analysis was conducted using two-way ANOVA followed by Tukey’s multiple comparison test; only the *p*-value for the most relevant comparisons are shown for simplicity. The intensities of bands were quantified by ImageJ. *p < 0.05, **p < 0.01, ***p < 0.001, ****p < 0.0001. Data underlying this figure can be found in S1 Data and S1 Raw Images. CMPK2, cytidine/uridine monophosphate kinase 2; dpi, days post-infection; hpi, hours post-infection; MOI, multiplicity of infection; PEDV, porcine epidemic diarrhea virus; qRT-PCR, quantitative real-time PCR.

To assay the expression levels of CMPK2 during PEDV infection in vivo, 7-day-old piglets were orally infected with PEDV at an inoculum of 2 × 10^5^ TCID_50_ and killed at 48 hpi. We quantified PEDV genome copy numbers, based on a standard curve (S2 Fig), and relative transcriptional levels of CMPK2 by quantitative real-time PCR (qRT-PCR). The results showed that CMPK2 was significantly up-regulated in all 3 segments of the small intestine with the most robust PEDV replication (Fig 1A and 1B). We further validated the RNA-sequencing results by qRT-PCR and western blotting in Vero cells and IPEC-J2 cells. The levels of CMPK2 were up-regulated on the mRNA and protein levels in Vero cells (Fig 1C) and IPEC-J2 cells (Fig 1D) compared with those in uninfected cells. Furthermore, we found that both the virulent strain AH2012/12 and the attenuated strain JS2008 of PEDV could induce CMPK2 expression (S3 Fig). In contrast, infection of ultraviolet-inactivated PEDV did not alter CMPK2 expression (S4 Fig). These results suggest that PEDV infection up-regulates the expression of CMPK2 both in vitro and in vivo and that this process is dependent on active viral replication.

### PEDV induced CMPK2 expression by type I IFN-dependent and independent signaling pathways

To dissect the upstream signaling responsible for CMPK2 up-regulation by PEDV infection, we selected specific inhibitors that target different steps of the IFN induction and response pathways including TBK1 (GSK8612), STAT1 (Fludarabine), and JAK1/2 (Ruxolitinib). The cytotoxicity of these inhibitors was tested, and at the concentrations used in this study, none of them induce more than 20% of cell death (S5 Fig).

**Fig 2:**
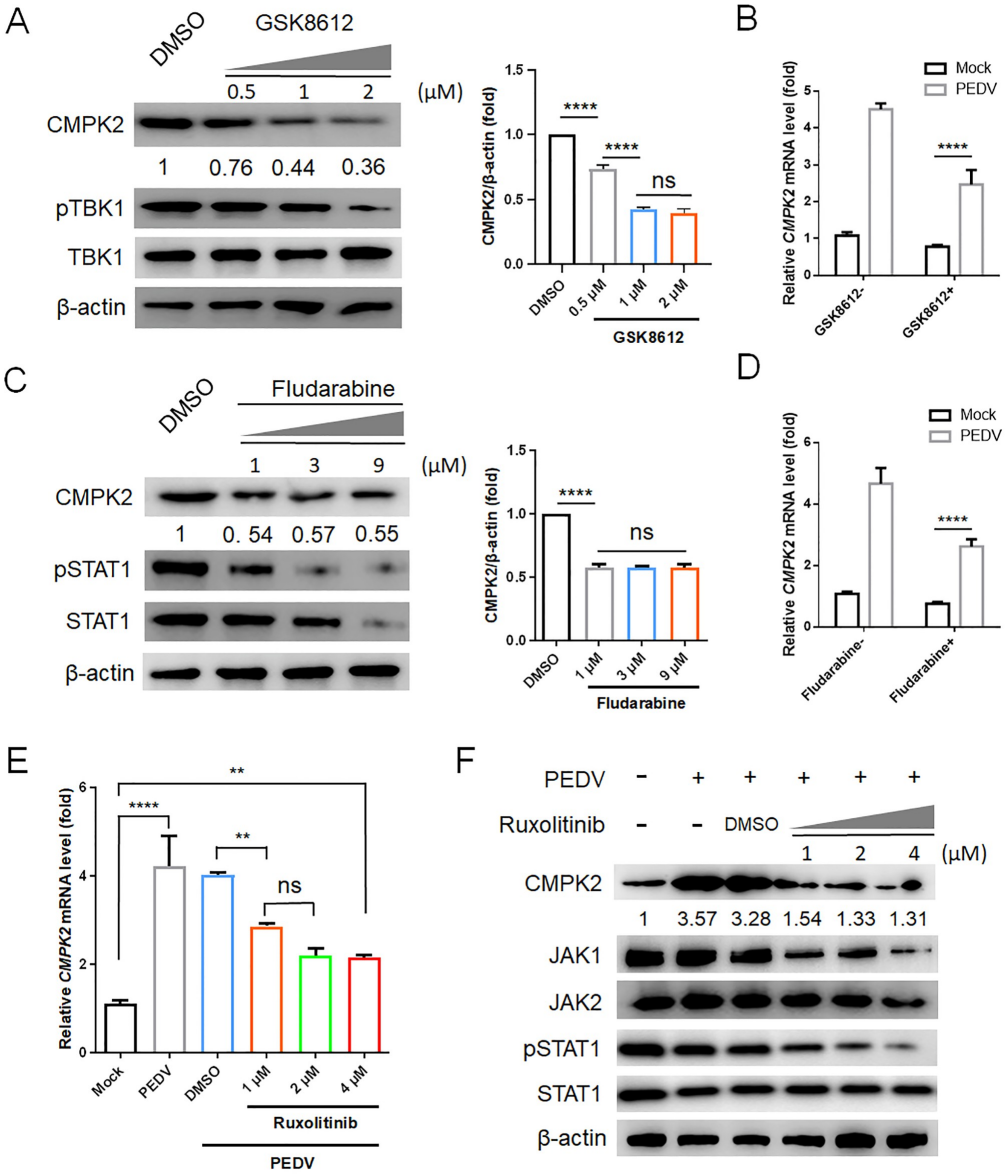
PEDV-induced CMPK2 expression is partially independent of the IFN-I pathway. **(A and C)** IPEC-J2 cells were treated with DMSO or different concentrations of GSK8612 (**A**) or Fludarabine (**C**) as indicated. The expression of CMPK2 were detected by western blot (left). Quantitative comparisons of the indicated protein levels were analyzed by gray intensity scanning of blots (right). **(B and D)** qRT-PCR analysis of CMPK2 mRNA levels in IPEC-J2 cells infected with PEDV (MOI = 1) followed by treatment with GSK8612 (**B**, 2 μM) or Fludarabine (**D**, 7 μM). **(E and F)** IPEC-J2 cells were treated with DMSO or different concentrations of Ruxolitinib as indicated. The expression of CMPK2 were detected by qRT-PCR (**E**) and western blot (**F**), respectively. Data are means ± SD of triplicate samples; statistical analysis was conducted using one-way ANOVA followed by Dunnett’s multiple comparison test or two-way ANOVA followed by Tukey’s multiple comparison test; only the *p*-value for the most relevant comparisons are shown for simplicity. The intensities of bands were quantified by ImageJ. **p < 0.01. ****p < 0.0001. Data underlying this figure can be found in S1 Data and S1 Raw Images. CMPK2, cytidine/uridine monophosphate kinase 2; IFN-I, type I interferon; MOI, multiplicity of infection; ns, no significance; PEDV, porcine epidemic diarrhea virus; qRT-PCR, quantitative real-time PCR.

Subsequently, PEDV-infected IPEC-J2 cells were treated with different concentrations of GSK8612 and harvested at 16 h post-treatment. Although phosphorylation of TBK1 was strongly inhibited by GSK8612 treatment, CMPK2 expression was not inhibited to a greater extent (Fig 2A). Compared to several classical ISGs such as CXCL10, IFIT1, and MX1, the transcriptional level of CMPK2 was still significantly up-regulated by PEDV infection even in the presence of high concentrations of GSK8612 (Fig 2B and S6A Fig). Likewise, bulk and phosphorylated STAT1 levels were strongly inhibited and the transcriptional levels of classical ISGs were also down-regulated after Fludarabine treatment (Fig 2C and S6B Fig). Contrarily, CMPK2 expression was still at least partially up-regulated by PEDV infection (Fig 2C and 2D). To further confirm that CMPK2 could be induced by PEDV infection partially independent of IFN-I pathway, IPEC-J2 cells were additionally treated with different concentrations of Ruxolitinib. CMPK2 was still significantly up-regulated by PEDV infection unlike classical ISGs with high concentrations of Ruxolitinib treatment (Fig 2E and 2F, and S6C Fig). These results suggested that CMPK2 up-regulated by PEDV infection takes place via IFN-I-dependent and independent pathways.

### IRF1 is crucial for the transcriptional activation of CMPK2

**Fig 3:**
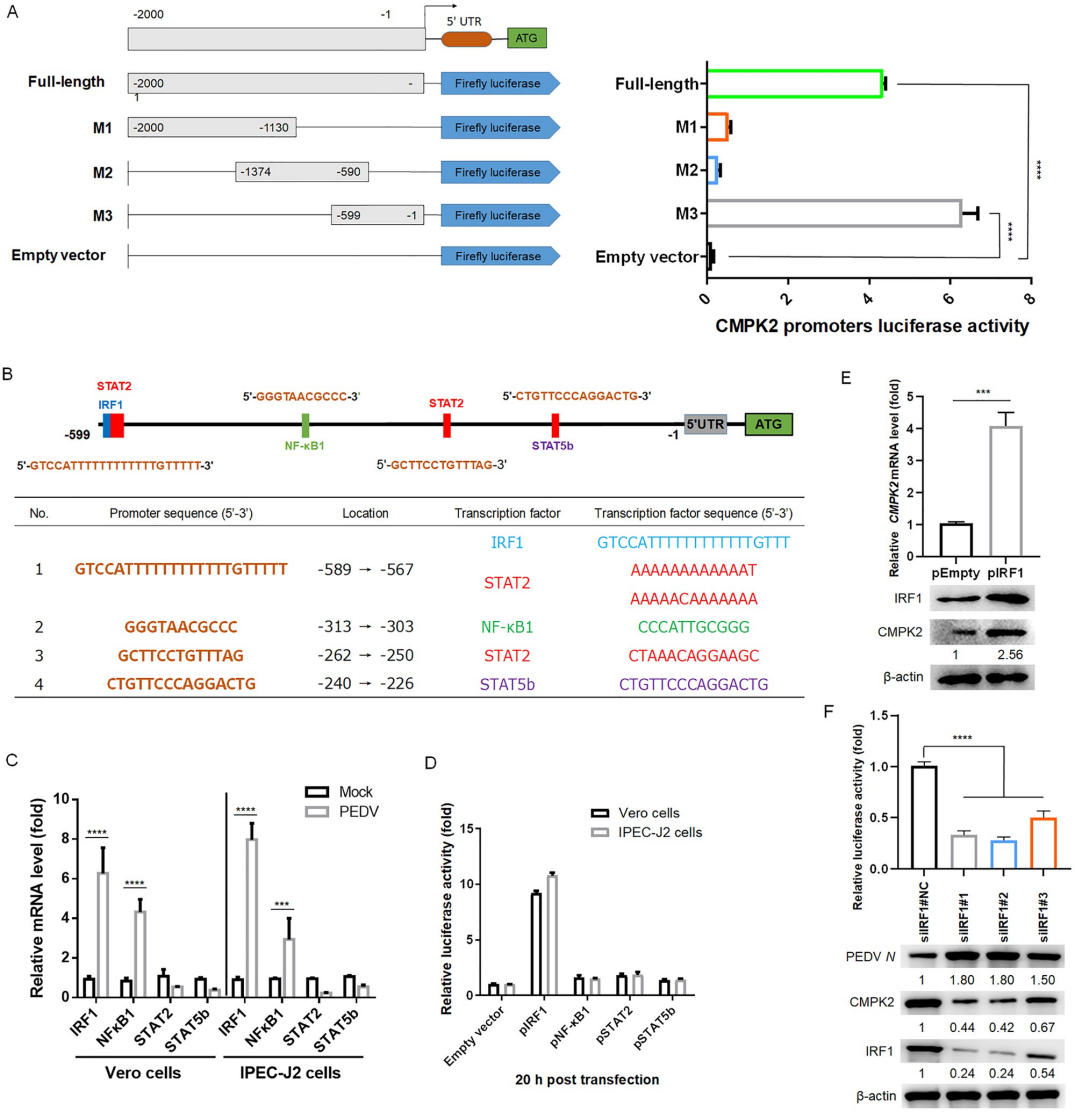
IRF1 directly controls the transcription of CMPK2. **(A)** Full-length *CMPK2* promoter (nucleotides −2,000 to −13) and a series of truncated *CMPK2* promoter mutants (M1 to M3) cloned into the pGL3-Basic vector. IPEC-J2 cells were cotransfected with the full-length or truncated promoter–reporter constructs, together with the Renilla luciferase reporter vector (pRL-TK-luc) as an internal control. Samples were collected at 20 h post-transfection and analyzed for dual luciferase activities. **(B)** Regulatory elements in the *CMPK2* promoter region were predicted with the JASPAR vertebrate database (http://jaspar.genereg.net/). Different colors indicate different putative regulatory elements. **(C)** Vero cells and IPEC-J2 cells were infected or mock-infected with PEDV (MOI = 1) and harvested at 20 hpi. The expression of putative transcription factors was analyzed by qRT-PCR. **(D)** IPEC-J2 cells were transfected with *CMPK2* promoter-driven luciferase vector, pRL-TK-luc vector, and plasmids encoding Flag-tagged putative transcription factors. Samples were collected at 20 h post-transfection and analyzed for dual luciferase activity. **(E and F)** IPEC-J2 cells were transfected with either plasmid encoding Flag-IRF1 (pIRF1) or siRNA#1 silencing IRF1 and then infected with PEDV at MOI of 1. qRT-PCR and western blotting were performed to measure the levels of CMPK2 expression and PEDV replication. Data are means ± SD of triplicate samples; statistical analysis was conducted using one-way ANOVA followed by Dunnett’s multiple comparison test or two-way ANOVA followed by Tukey’s multiple comparison test; only the *p*-value for the most relevant comparisons are shown for simplicity. ***p < 0.001, ****p < 0.0001. Data underlying this figure can be found in S1 Data and S1 Raw Images. CMPK2, cytidine/uridine monophosphate kinase 2; hpi, hours post-infection; IRF1, interferon regulatory factor 1; MOI, multiplicity of infection; PEDV, porcine epidemic diarrhea virus; qRT-PCR, quantitative real-time PCR.

Interferon regulatory factors (IRFs) 1/3/7 and NF-κB are activated during virus infection and induce the production of downstream antiviral genes [31,32]. To examine the identity of transcriptional factors in regulating CMPK2 expression, we generated a luciferase reporter under the control of 2,000 base pairs (bp) of the porcine *CMPK2* promoter sequences (S1 Table and Fig 3A). Subsequently, we further constructed 3 truncated promoters (designated M1 to M3) and tested the luciferase activity in HEK293T cells. PEDV infection induced similar levels of luciferase expression from the promoter deletion mutants containing nucleotides from −599 to −1 (M3) and the full-length promoter construct (Fig 3A). To confirm the IFN responsiveness of the promoter, we analyzed the transcription and expression levels of CMPK2 in IPEC-J2 cells after different concentrations of IFN-α1 supplementation and then detected the CMPK2 promoter luciferase activity. We found that CMPK2 was significantly up-regulated by IFN-α1 supplementation, corresponding to an activated CMPK2 promoter activity (S7A and S7B Fig). To analyze the transcriptional regulation of the CMPK2 gene, we examined the promoter for potential transcription factor binding sites (TFBS) using the JASPAR vertebrate database (http://jaspar.genereg.net/) [33]. We found that the *CMPK2* promoter region M3, spanning nucleotide positions −599 to −1, contains several TFBS, including IRF1, NF-κB1, STAT2, and STAT5b binding sites (Fig 3B). Meanwhile, the mRNA abundance of these transcriptional factors was detected in PEDV-infected cells. We found that IRF1 and NF-κB1 were both up-regulated during PEDV infection in Vero and IPEC-J2 cells (Fig 3C). To assess the effects of putative transcriptional factors that regulate CMPK2 expression, we ectopically expressed individual genes in HEK293T cells transfected with the *CMPK2* promoter-driven luciferase vector. Cells overexpressing IRF1, but not other transcription factors, showed significantly increased luciferase expression driven by the *CMPK2* promoter (Fig 3D). To further confirm that IRF1 is involved in the regulation of CMPK2 expression, we performed a chromatin immunoprecipitation (ChIP) assay and found that IRF1 directly bound to the *CMPK2* promoter region in IPEC-J2 cells (S8 Fig).

In addition to HEK293T cells, we examined the role of IRF1 in endogenous CMPK2 expression in IPEC-J2 cells. We found that elevated CMPK2 protein levels post-IRF1 overexpression (Fig 3E). Consistently, CMPK2 induction was suppressed in PEDV-infected IPEC-J2 cells transfected with specific small interfering RNAs (siRNAs) against IRF1 (Fig 3F). Collectively, these results demonstrate that IRF1 binds to the *CMPK2* promoter and activates CMPK2 expression.

### CMPK2 inhibits PEDV replication in Vero and IPEC-J2 cells

**Fig 4:**
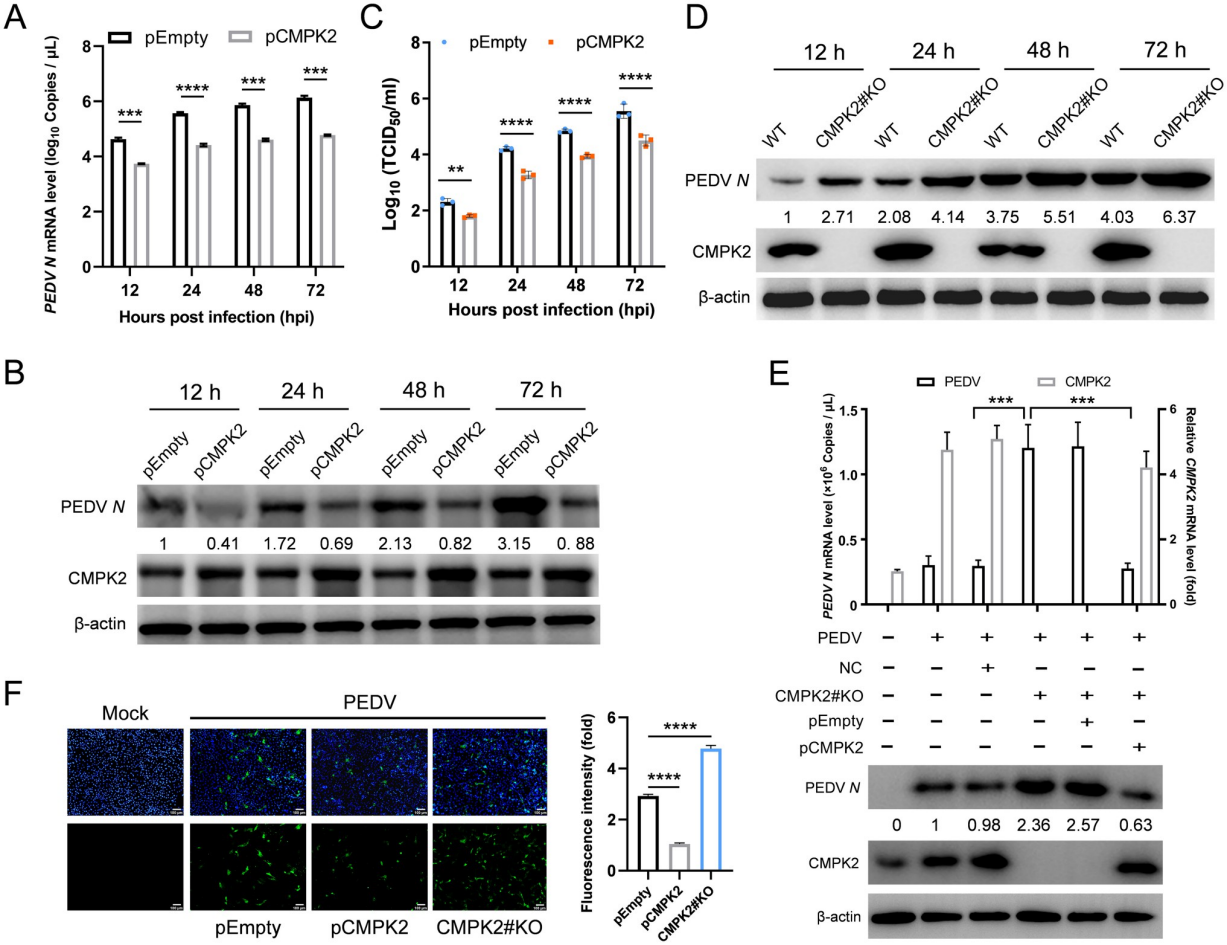
CMPK2 inhibits PEDV replication in IPEC-J2 cells. **(A-C)** IPEC-J2 cells were transfected with empty vector (pEmpty) and pCMPK2 for 24 h and then infected with PEDV at MOI of 1. The cells and culture supernatants were harvested at indicated time points. Total RNA of the cells was extracted and analyzed by qRT-PCR (**A**), and the cell lysates were analyzed by western blotting (**B**). PEDV titers in the culture supernatants were measured as TCID_50_ (**C**). **(D)** *CMPK2* knockout (*CMPK2#KO*) IPEC-J2 cells were constructed and then infected with PEDV as above. The cells were harvested at indicated time points, and the lysates were analyzed by western blotting. **(E)** *CMPK2* knockout IPEC-J2 cells were transfected with pEmpty and pCMPK2 for 24 h and then infected with PEDV as above. Total RNA of the cells was extracted and analyzed by qRT-PCR (upper panel), and the cell lysates were analyzed by western blotting (lower panel). **(F)** PEDV *N* antigen was detected in infected cells by indirect IFA. PEDV *N* (green), DAPI (blue). Scale bars, 100 μm. Data are means ± SD of triplicate samples; statistical analysis was conducted using one-way ANOVA followed by Dunnett’s multiple comparison test or two-way ANOVA followed by Tukey’s multiple comparison test; only the *p*-value for the most relevant comparisons are shown for simplicity. The intensities of bands were quantified by ImageJ. **p < 0.01, ***p < 0.001, ****p < 0.0001. Data underlying this figure can be found in S1 Data and S1 Raw Images. CMPK2, cytidine/uridine monophosphate kinase 2; IFA, immunofluorescent assay; MOI, multiplicity of infection; PEDV, porcine epidemic diarrhea virus; qRT-PCR, quantitative real-time PCR.

To investigate the potential antiviral activity of CMPK2 toward PEDV replication, we transfected Vero and IPEC-J2 cells with empty vector or CMPK2 encoding plasmids and then infected with PEDV at MOI of 1. Cells were harvested at indicated time points and analyzed by qRT-PCR and western blotting. We found that the transcript and protein levels of PEDV *N* were significantly lower in the IPEC-J2 cells overexpressing CMPK2 than those transfected with the empty vector (Fig 4A and 4B). Cell culture supernatants were also collected, and viral titers were determined by limiting serial dilutions. The amount of infectious PEDV in the supernatants of IPEC-J2 cells overexpressing CMPK2 was significantly lower than those in the cells transfected with empty vector (Fig 4C). Similar findings were validated in Vero cells that are traditionally known to be IFN-insensitive. The transcript and protein levels of PEDV *N* were significantly inhibited (S9A and S9B Fig), and the viral titers in the supernatants were decreased approximately 10-fold in Vero cells with CMPK2 overexpression (S9C Fig). We also sought to determine the physiological relevance of endogenous CMPK2. We transfected Vero cells with siRNAs targeting simian CMPK2 and infected with PEDV at MOI of 1 and harvested at 20 hpi. Total RNA of the cells was extracted and analyzed by qRT-PCR, and the cell lysates were analyzed by western blotting. We found that the transcript and protein levels of PEDV *N* were elevated in the Vero cells transfected with siRNA targeting CMPK2 than in cells transfected with siRNA-negative control (S9D and S9E Fig). Moreover, the viral titers in the supernatants were approximately 10-fold higher than in Vero cells with CMPK2 siRNA knockdown (S9F Fig).

We further confirmed the inhibition of PEDV replication by CMPK2 by generating single clonal *CMPK2* knockout IPEC-J2 cells via CRISPR-Cas9. The expression of PEDV *N* was significantly increased in *CMPK2* knockout IPEC-J2 cells and reversed after complementing with exogenous expression of wild-type full-length CMPK2 (Fig 4D and 4E). Indirect immunofluorescent assay (IFA) also confirmed that PEDV *N* expression was decreased with CMPK2 overexpression and increased in *CMPK2* knockout IPEC-J2 cells (Fig 4F). Taken together, these results indicate that CMPK2 is a potent host antiviral factor of PEDV replication.

### CMPK2 inhibits PEDV infection at a post-entry step

We next sought to define the step of the PEDV replication cycle that was restricted by CMPK2. IPEC-J2 cells with or without CMPK2 overexpression were incubated with PEDV at MOI of 5 (4 °C for 1 h), washed extensively with phosphate-buffered saline (PBS), and the genome copies of bound viruses was measured by qRT-PCR. In parallel, CMPK2 transfected IPEC-J2 cells were incubated with PEDV at MOI of 1 (4 °C for 1 h) and shifted to 37 °C for 1 h. The cells were then washed to remove all unbound and unendocytosed virus, and the intracellular viral RNA was quantified using qRT-PCR (S10A Fig). The results showed that there was no significant difference in the amount of PEDV bound or internalized between empty vector and CMPK2 transfected cells (S10B Fig), suggesting that overexpression of CMPK2 does not act to block the process of viral binding or entry. Kinetic studies were further performed to investigate the antiviral action of CMPK2 on PEDV replication. Transcriptional levels of PEDV in IPEC-J2 cells transfected with CMPK2 were analyzed by qRT-PCR at 1, 2, 3, and 4 hpi, respectively, and PEDV *N* expression was detected by western blotting. Despite no significant differences in intracellular viral RNA levels at 1 hpi (S10C Fig), the amount of viral RNA levels was readily reduced at later time points in CMPK2 expressing IPEC-J2 cells (S10C and S10D Fig), suggesting that CMPK2 likely restricts PEDV replication at the stage of transcription or genome replication.

### ddhCTP catalysis by Viperin is required for the antiviral activity of CMPK2

**Fig 5:**
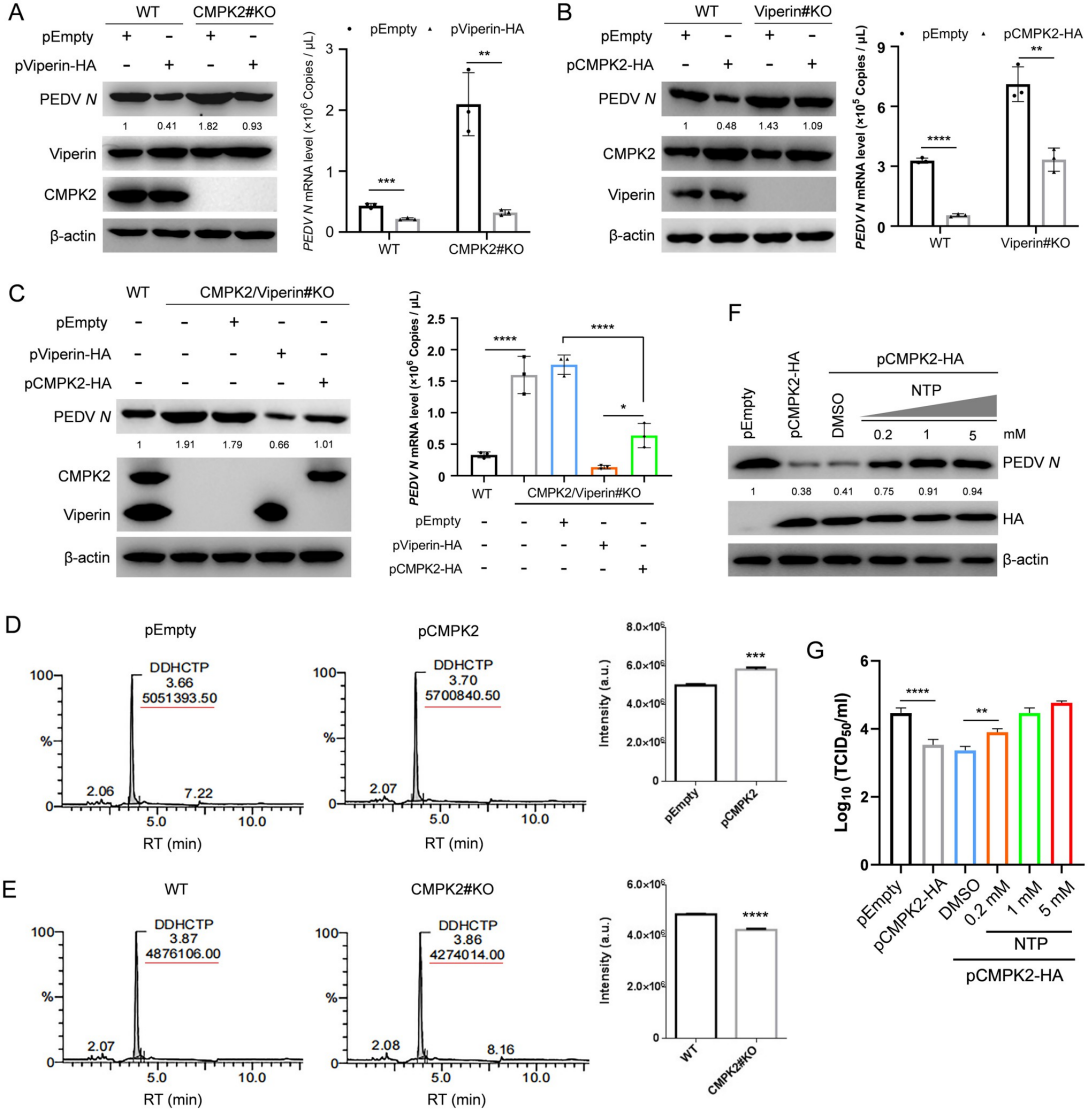
ddhCTP catalyzed by Viperin is crucial for CMPK2 inhibition of PEDV replication. **(A)** CMPK2#KO IPEC-J2 cells were transfected with pEmpty and pViperin-HA for 24 h and then infected with PEDV at MOI of 1. The cells were harvested at 20 hpi, and the lysates were analyzed by western blotting (left). Total RNA of the cells was extracted, and viral copy numbers were analyzed by qRT-PCR (right). **(B)** Viperin knockout (Viperin#KO) IPEC-J2 cells were constructed and then transfected with pEmpty and pCMPK2-HA for 24 h. Subsequently, Viperin#KO IPEC-J2 cells were infected with PEDV as above. The lysates were analyzed by western blotting (left). Total RNA of the cells was extracted, and viral copy numbers were analyzed by qRT-PCR (right). **(C)** CMPK2/Viperin double KO (*Viperin/CMPK2*#KO) IPEC-J2 cells were constructed and, respectively, transfected with pEmpty, pViperin-HA, and pCMPK2-HA for 24 h to rescue Viperin or CMPK2 expression and then infected with PEDV as above. The lysates were analyzed by western blotting (left). Total RNA of the cells was extracted, and viral copy numbers were analyzed by qRT-PCR (right). **(D)** ddhCTP production in IPEC-J2 cells with or without CMPK2 expression was determined by LC–MS. **(E)** The detection of ddhCTP production in CMPK2 knockout or wild-type IPEC-J2 cells by LC–MS. **(F and G)** IPEC-J2 cells were transfected with pCMPK2-HA and empty vector, and a range of concentrations of NTPs were added to compete with ddhCTP. The cell lysates were analyzed by western blotting (**F**), and PEDV titers in the culture supernatants were measured as TCID_50_ (**G**). Data are means ± SD of triplicate samples; statistical analysis was conducted using one-way ANOVA followed by Dunnett’s multiple comparison test or two-way ANOVA followed by Tukey’s multiple comparison test; only the *p*-value for the most relevant comparisons are shown for simplicity. The intensities of bands were quantified by ImageJ. *p < 0.05, **p < 0.01, ***p < 0.001, ****p < 0.0001. Data underlying this figure can be found in S1 Data and S1 Raw Images. CMPK2, cytidine/uridine monophosphate kinase 2; ddhCTP, 3′-deoxy-3′,4′-didehydro-cytidine triphosphate; hpi, hours post-infection; LC–MS, liquid chromatography followed by mass spectrometry; MOI, multiplicity of infection; NTP, nucleotide triphosphate; PEDV, porcine epidemic diarrhea virus; qRT-PCR, quantitative real-time PCR.

It has been reported that Viperin inhibits viral replication by converting CTP into ddhCTP and that CMPK2 provides enough CTP as a substrate in this process [25,34]. To investigate the molecular mechanisms by which CMPK2 suppresses PEDV replication, we tested the effect of siRNAs targeting porcine Viperin (siViperin) on PEDV infection. IPEC-J2 cells with or without CMPK2 overexpression were transfected with siRNA targeting Viperin or the negative control and then infected with PEDV at MOI of 1. The expression levels of PEDV *N* were significantly decreased in IPEC-J2 cells overexpressing CMPK2, and the reduction was partially prevented by Viperin knockdown (S11A and S11B Fig). To further tease apart the upstream–downstream relationship and investigate the molecular mechanisms by which CMPK2 suppresses PEDV replication, *Viperin* knockout and *Viperin/CMPK2* double knockout IPEC-J2 cells were constructed and changes in PEDV replication levels were analyzed after supplementing Viperin in *CMPK2* knockout cells or supplementing CMPK2 in *Viperin* knockout cells. We found that Viperin supplementation in *CMPK2* knockout cells had a more significant effect on inhibiting PEDV replication than the reciprocal add-back (Fig 5A and 5B). Meanwhile, we also complemented Viperin and CMPK2, respectively, in the double knockout cells. Consistently, the effect of Viperin on PEDV replication appears to be more direct (Fig 5C). Of note, Viperin and CMPK2 are not entirely dependent on each other. Previous studies showed an independent transcriptional regulation and that these 2 factors may restrict viral replication either together or individually in different cellular contexts [17,24]. Taken together, these results confirmed that CMPK2 is an important host factor upstream of Viperin.

We next determined production of ddhCTP, the end product of CMPK2 and Viperin, with liquid chromatography followed by mass spectrometry (LC–MS) in IPEC-J2 cells previously transfected with mock or Viperin targeting siRNAs as previously described [35]. Consistent with the previous reports [36], ddhCTP level was significantly decreased with the knockdown of Viperin (S11C Fig). To clarify the relationship between CMPK2 and ddhCTP, we also determined ddhCTP production by LC–MS in IPEC-J2 cells with CMPK2 overexpression or knockout. As expected, ddhCTP level was significantly increased in IPEC-J2 cells with CMPK2 overexpression and substantially decreased with CMPK2 knockout (Fig 5D and 5E). To further analyzed the effect of ddhCTP on CMPK2 antiviral activity, IPEC-J2 cells were transfected with CMPK2 and empty vector, and a range of concentrations of nucleotide triphosphates (NTPs) were added to compete with ddhCTP and complement the loss of CTP. Even in the presence of CMPK2, expression levels of PEDV *N* and viral titers were restored with exogenous NTP supplementation (Fig 5F and 5G). Collectively, these results suggested that ddhCTP catalyzed by Viperin is required for CMPK2 restriction of PEDV replication.

### Amino acids 259–266 and D330 are essential for the antiviral activity of CMPK2

**Fig 6:**
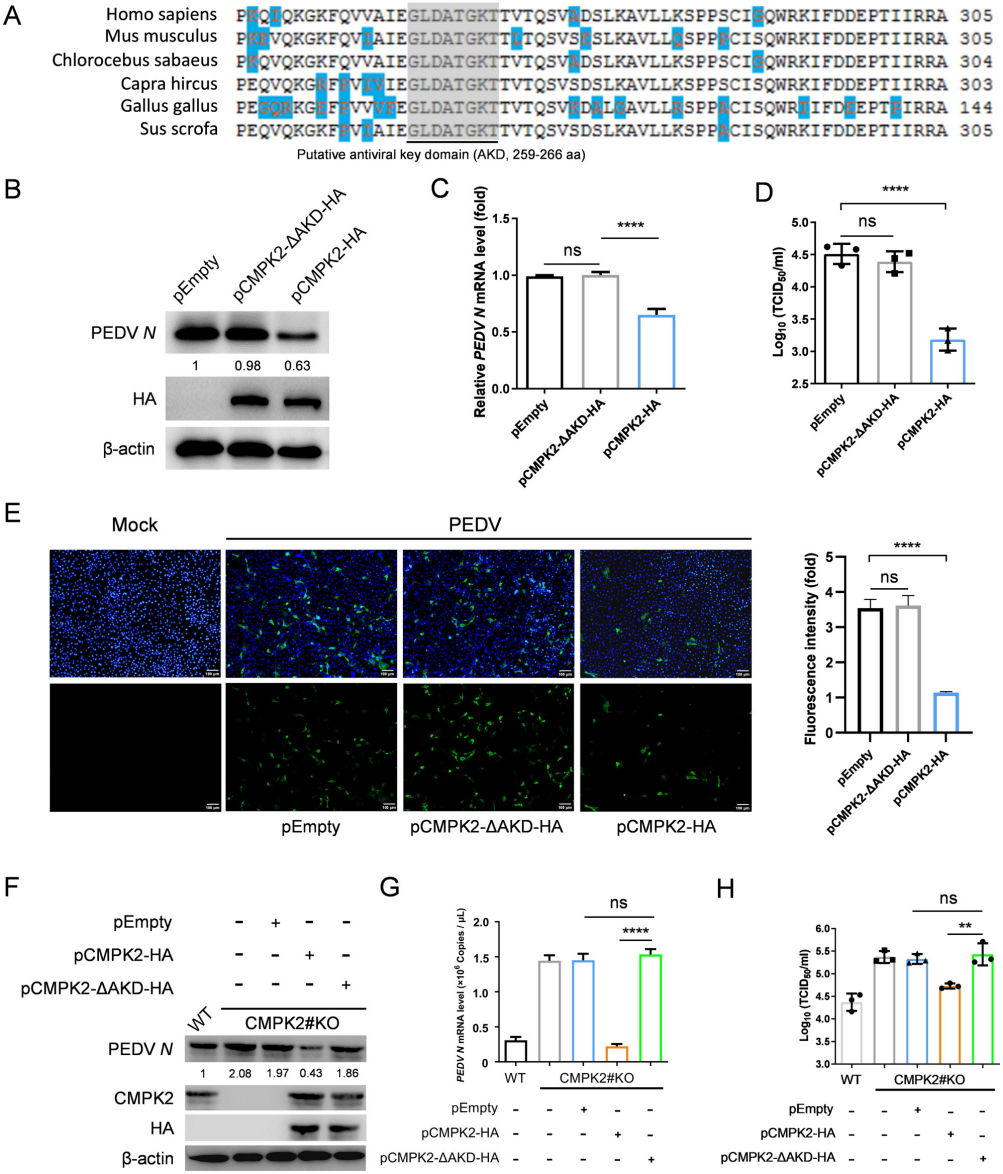
AKD domain is essential for CMPK2 exerting antiviral activity. **(A)** Alignment of *Homo sapiens* (NM_207315.4), *Mus musculus* (NM_020557.4), *Chlorocebus sabaeus* (XM_007971579.2), *Capra hircus* (XM_018055703.1), *Gallus gallus* (XM_015284945.4), and *Sus scrofa* (XM_021087907.1) CMPK2 amino acid sequences using the Megalign software, and the AKD was predicted using the UniProt database. Gray indicates sequence conservation. Blue indicates different amino acids among the above species. **(B-C)** IPEC-J2 cells were transfected with CMPK2-ΔAKD-HA, CMPK2-HA, and empty vector followed by PEDV infection for 20 hpi. The cell lysates were analyzed by western blotting (**B**), and total RNA of the cells were extracted and analyzed by qRT-PCR (**C**). **(D)** Culture supernatants were collected at 20 hpi, and PEDV titers were measured as TCID_50_. **(E)** PEDV in the cells overexpression CMPK2 with or without AKD deletion was detected by indirect IFA. PEDV *N* (green), DAPI (blue). Scale bars, 100 μm. **(F-G)** CMPK2 knockout IPEC-J2 cells were transfected with CMPK2-ΔAKD-HA and empty vector for 24 h and then infected PEDV infection at MOI of 1 for 20 h. The cell lysates were analyzed by western blotting (**F**), and total RNA of the cells were extracted and analyzed by qRT-PCR (**G**). **(H)** Culture supernatants were collected at 20 hpi, and PEDV titers were measured as TCID_50_. Data are means ± SD of triplicate samples; statistical analysis was conducted using one-way ANOVA followed by Dunnett’s multiple comparison; only the *p*-value for the most relevant comparisons are shown for simplicity. The intensities of bands were quantified by ImageJ. ns, no significance. **p < 0.01. ****p < 0.0001. Data underlying this figure can be found in S1 Data and S1 Raw Images. AKD, antiviral key domain; CMPK2, cytidine/uridine monophosphate kinase 2; hpi, hours post-infection; IFA, immunofluorescent assay; MOI, multiplicity of infection; PEDV, porcine epidemic diarrhea virus; qRT-PCR, quantitative real-time PCR.

Thus far, our data have implicated that CMPK2 inhibition of viral replication correlates with the cytidine monophosphate kinase activity, which provides CTP for Viperin to catalyze the production of ddhCTP. To pinpoint the region of CMPK2 required for the antiviral activity, the amino acid sequences of CMPK2 across different species were analyzed. We identified a stretch of amino acids -*GLDATGKT*- (259–266 aa) that is highly conserved in full-length CMPK2 across different species, implicating this region to be a predicted AKD for further examination (Fig 6A and S12A Fig). To verify the functional relevance of CMPK2 AKD to ddhCTP production, we constructed an AKD deletion mutant (pCMPK2-ΔAKD-HA), which we introduced into IPEC-J2 cells followed by LC–MS analysis. The results demonstrated that the AKD of CMPK2 is crucial for ddhCTP production (S13 Fig).

We next sought to determine the antiviral activities of CMPK2 truncation constructs. IPEC-J2 cells were transfected with pCMPK2-ΔAKD-HA, pCMPK2-HA, and empty vector followed by PEDV infection for 20 h. We found that the inhibitory effect of CMPK2 on PEDV replication was almost completely abolished in the absence of AKD (Fig 6B and 6C). The viral titers in the supernatants of the IPEC-J2 cells overexpressing CMPK2 were significantly lower than those in the cells transfected with pCMPK2-ΔAKD-HA (Fig 6D). Immunofluorescence staining also indicated that CMPK2 antiviral activity was abolished with the deletion of AKD (Fig 6E).

Since we have previously shown that PEDV replication was restricted by CMPK2 complementation of *CMPK2* knockout IPEC-J2 cells, we transfected *CMPK2* knockout IPEC-J2 cells with empty vector, pCMPK2-HA, and pCMPK2-ΔAKD-HA followed by PEDV infection for 20 h to further assess the influence of AKD on CMPK2 antiviral activity. As expected, unlike the wild-type construct, CMPK2 with AKD deletion did not inhibit PEDV replication (Fig 6F and 6G). Consistent with this, PEDV titers did not decrease by CMPK2 mutant expression in IPEC-J2 knockout cells (Fig 6H). Previous studies have identified a highly conserved aspartate (D) residue in its catalytic pocket that is important for CMPK2’s antiviral activity [12] (S12B Fig). The catalytic activity of CMPK2 was abrogated when aspartate (D) residue was replaced with alanine (A) [16,37]. Therefore, we constructed the reported catalytic mutant of CMPK2 (CMPK2-D330A-HA) as a control to further clarify whether the key domain has catalytic activity independent of the classical catalytic domain. The highly conserved aspartate (D) residue was crucial for CMPK2 catalytic activity and that both the classical catalytic domain and the identified AKD domain here combinatorially contributed to CMPK2 antiviral activity (S12C–S12E Fig). These results collectively demonstrated that the putative AKD, in addition to D330, is essential for CMPK2 inhibition of PEDV replication.

### CMPK2 inhibits multiple coronaviruses by reducing RdRp activity

**Fig 7:**
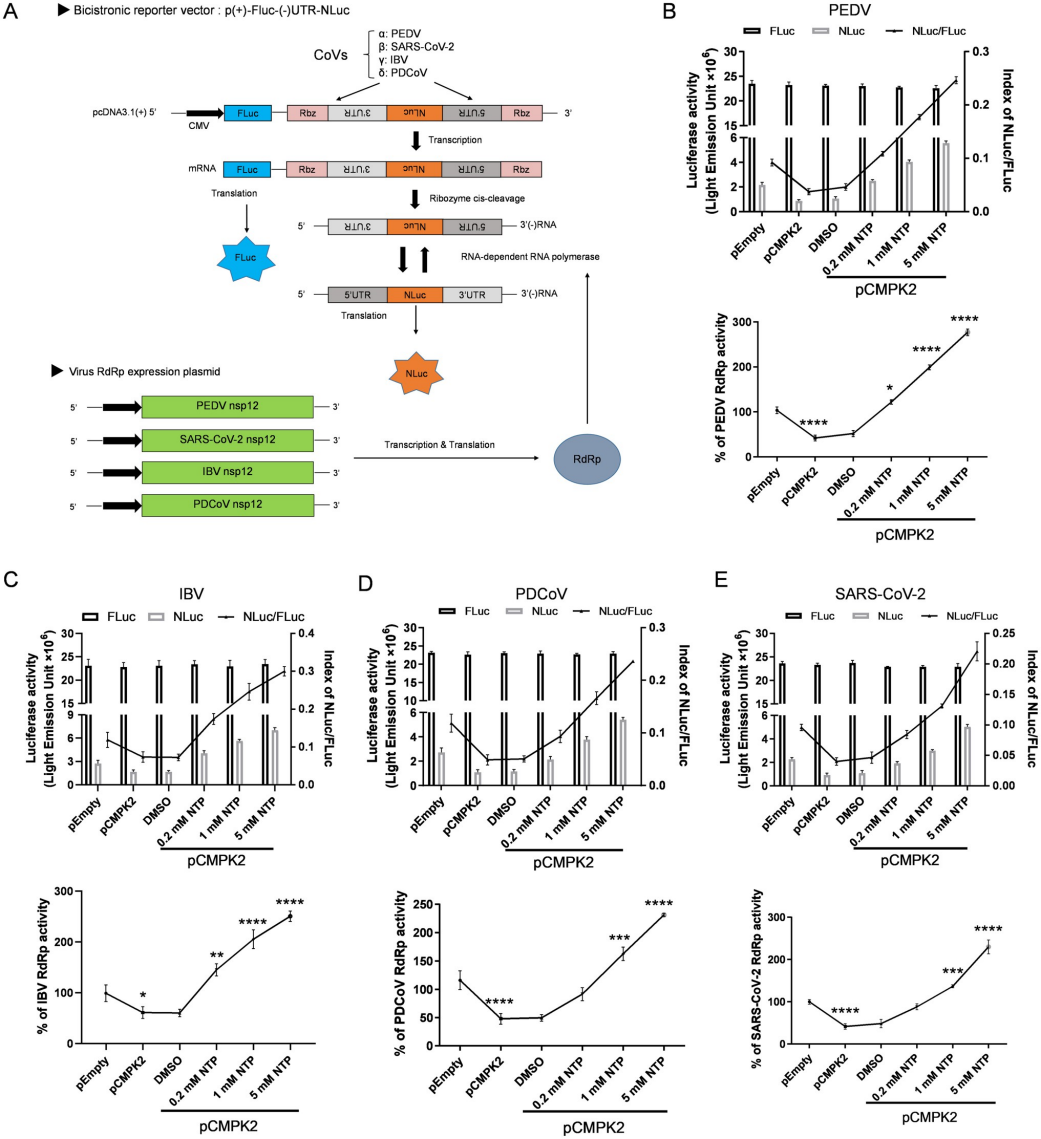
CMPK2 inhibits the RdRp activity of multiple CoVs. **(A)** Schematic diagram of the cell-based reporter assay system for CoV RdRp activity. **(B)** HEK293T cells were transfected with empty vector and pCMPK2 and then treated with different concentrations of NTP. Effects of CMPK2 on PEDV RdRp activity were detected by the cell-based reporter assay system. **(C-E)** HEK293T cells were handled like (**B**) and the effects of CMPK2 on IBV (**C**), PDCoV (**D**), and SARS-CoV-2 (**E)** RdRp activity were, respectively, detected by the cell-based reporter assay system. Data are means ± SD of triplicate samples; statistical analysis was conducted using two-tailed Student *t* test; only the *p*-value for the most relevant comparisons are shown for simplicity. *p < 0.05, **p < 0.01, ***p < 0.001, ****p < 0.0001. Data underlying this figure can be found in S1 Data. CMPK2, cytidine/uridine monophosphate kinase 2; CoV, coronavirus; IBV, infectious bronchitis virus; NTP, nucleotide triphosphate; PDCoV, porcine delta-coronavirus; PEDV, porcine epidemic diarrhea virus; RdRp, RNA-dependent RNA polymerase; SARS-CoV-2, Severe Acute Respiratory Syndrome Coronavirus 2.

Because CMPK2 mediates the production of ddhCTP, which is a chain terminator for RdRp [25,38], we set up an RdRp activity assay by constructing reporter plasmids based on PEDV, IBV, PDCoV, and SARS-CoV-2. The bicistronic reporter plasmid contains a sense-oriented firefly luciferase sequence [(+)FLuc] under the control of a CMV promoter to act as an internal control and an antisense-oriented Nano-Glo luciferase sequence [(−)NLuc] flanked by HDV ribozyme (Rbz) self-cleavage sequences and antisense 5′ UTR and 3′ UTR of CoVs. The full-length (+)FLuc-(−)UTR-NLuc RNA is transcribed by the host and then cleaved at the HDV ribozyme self-cleavage site. Subsequently, the cleaved antisense 3′ UTR-(−)NLuc-antisense 5′ UTR RNA sequences were replicated by CoV RdRps. Finally, the expression of NLuc was measured as an indicator of CoV RdRp activities (Fig 7A).

We first tested the PEDV RdRp activity using the above assay system. We found that the NLuc activity and the ratio of NLuc and FLuc, reflective of PEDV RdRp activities, were decreased by CMPK2 overexpression (Fig 7B). Importantly, a defect in the PEDV RdRp activity was rescued by exogenous NTP supplementation in a dose-dependent manner even with CMPK2 overexpression (Fig 7B). We further explored the potential inhibitory effects on a γ CoV (represented by IBV) and a δ CoV (represented by PDCoV) RdRp by CMPK2. Interestingly, compared with those of PEDV, IBV RdRp activities were only slightly inhibited by CMPK2 and rescued by exogenous NTP supplementation (Fig 7C). Similarly, PDCoV RdRp activities were significantly inhibited by CMPK2 and rescued by exogenous NTP supplementation (Fig 7D). Subsequently, we examined the potential inhibition of a β CoV RdRp by CMPK2, represented by SARS-CoV-2. As expected, SARS-CoV-2 RdRp activities were inhibited by CMPK2 in a similar manner as that of PEDV (Fig 7E). Consistent with the above results, inhibition of SARS-CoV-2 RdRp activities was reversed by NTP treatment in a dose-dependent manner in the presence of CMPK2 overexpression (Fig 7E). We validated our findings in HEK293T cells in a panel of more physiologically relevant cell types, including IPEC-J2, Vero, DF-1, and Lilly Laboratories cell porcine kidney 1 (LLC-PK1) cells. Mutant CMPK2 with AKD deletion was unable to inhibit the different CoV RdRp activities, suggesting that AKD is essential for CMPK2 inhibition of CoV RdRp activities (S14A–S14D Fig).

**Fig 8:**
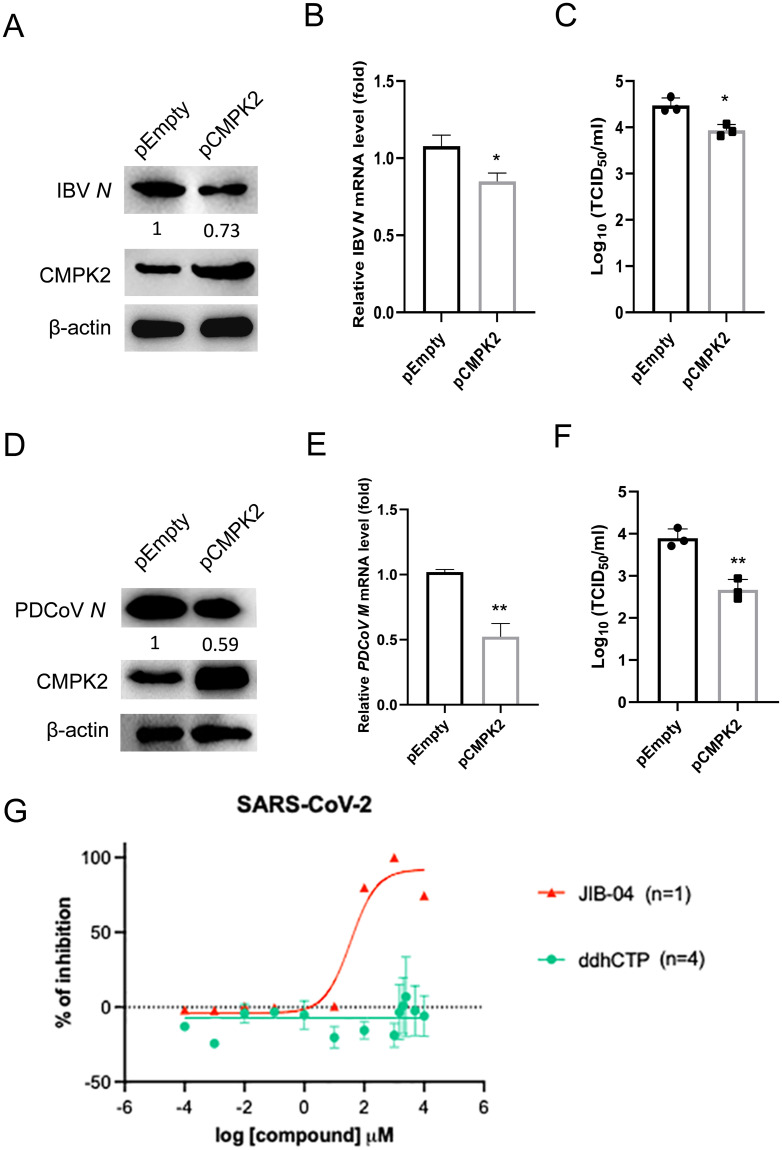
CMPK2 inhibits CoV replication with species differences. **(A-C)** DF-1 cells were transfected with empty vector and pCMPK2 for 24 h and then infected with IBV at MOI of 5. The cell lysates harvested at 20 hpi were analyzed by western blotting (**A**), total RNA of the cells was extracted and analyzed by qRT-PCR (**B**), and PDCoV titers in the culture supernatants were measured as TCID_50_ (**C**). **(D-F)** LLC-PK1 cells were transfected with empty vector and pCMPK2 for 24 h and then infected with PDCoV at MOI of 0.1. The cell lysates harvested at 20 hpi were analyzed by western blotting (**D**), total RNA of the cells was extracted and analyzed by qRT-PCR (**E**), and PDCoV titers in the culture supernatants were measured as TCID_50_ (**F**). **(G)** Dose–response curve of wild-type SARS-CoV-2 replication with JIB-04 or ddhCTP treatment. MA104 cells were treated with indicated compound for 1 h and infected with a clinical isolate of SARS-CoV-2 (MOI = 1). N mRNA levels were quantified at 24 hpi by qRT-PCR and plotted as percentage of inhibition. Data are means ± SD of triplicate samples; statistical analysis was conducted using one-way ANOVA followed by Dunnett’s multiple comparison or two-tailed Student *t* test; only the *p*-value for the most relevant comparisons are shown for simplicity. The intensities of bands were quantified by ImageJ. *p < 0.05, **p < 0.01. Data underlying this figure can be found in S1 Data and S1 Raw Images. CMPK2, cytidine/uridine monophosphate kinase 2; CoV, coronavirus; hpi, hours post-infection; IBV, infectious bronchitis virus; LLC-PK1, Lilly Laboratories cell porcine kidney 1; MOI, multiplicity of infection; PDCoV, porcine delta-coronavirus; qRT-PCR, quantitative real-time PCR; SARS-CoV-2, Severe Acute Respiratory Syndrome Coronavirus 2.

The inhibition of RdRp activities does not necessarily equate to suppression of viral replication. To that end, we investigated the possible antiviral effects of CMPK2 on the replication of other CoVs besides PDEV. DF-1 cells were transfected with empty vector or CMPK2 and then infected with IBV. We found that the transcript and protein levels of IBV *N* were modestly decreased by CMPK2 overexpression (Fig 8A and 8B). The viral titers in the supernatants of the DF-1 cells overexpressing CMPK2 were significantly lower than those in the cells transfected with empty vector (Fig 8C). Similarly, we examined the possible inhibitory effect of CMPK2 on PDCoV replication. LLC-PK1 cells were transfected with empty vector or CMPK2 and then infected with PDCoV. We found that the transcript levels of PDCoV *M* and protein levels of PDCoV *N* were significantly decreased by CMPK2 overexpression (Fig 8D and 8E). The viral titers in the supernatants of the LLC-PK1 cells overexpressing CMPK2 were also significantly lower than those in the cells transfected with empty vector (Fig 8F). Of note, unlike the positive control antiviral molecule JIB-04 [39], even at the highest concentrations tested, ddhCTP did not block SARS-CoV-2 replication (Fig 8G). Collectively, these results demonstrate that CMPK2 is a novel host restriction factor. While the inhibitory effect on viral replication was strain-specific, CMPK2 interferes with CoV RdRp activities in ddhCTP-dependent manner.

## Discussion

**Fig 9:**
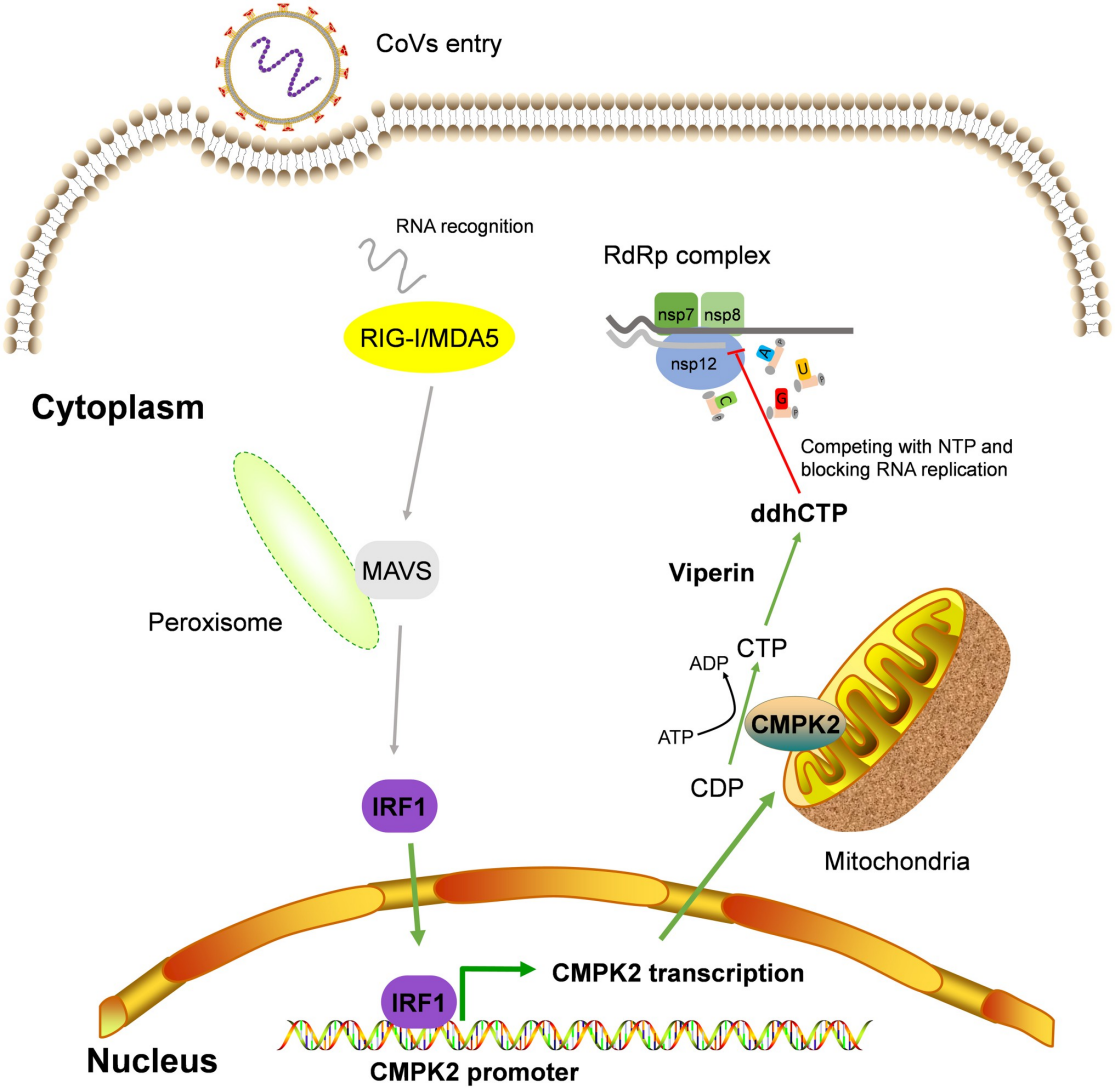
The graphical depiction of CMPK2 inhibition of CoVs replication by interfering with their RdRp activities. Upon CoV infection, IRF1 directly induces CMPK2 expression independently of the IFN-I pathway. CMPK2 localizes in mitochondria and provides sufficient substrate for Viperin-catalyzed production of ddhCTP through catalyzing the phosphorylation of CDP to produce CTP. Subsequently, ddhCTP directly stalls the progression of viral polymerase by competing with natural ribonucleotides. CMPK2, cytidine/uridine monophosphate kinase 2; CoV, coronavirus; ddhCTP, 3′-deoxy-3′,4′-didehydro-cytidine triphosphate; IFN-I, type I interferon; IRF1, interferon regulatory factor 1; RdRp, RNA-dependent RNA polymerase.

In a virus–host arms race, CoVs such as PEDV have evolved various strategies to evade the host antiviral innate immunity [21,40]. However, the host is still equipped with unique cell-intrinsic antiviral mechanisms, such as BST-2, which can be directly up-regulated by IRF1, and inhibits viral replication by tethering enveloped virions to the cell surface to restrict viral release [33]. Although African Green Monkey Vero cells naturally lack IFN-I gene [22], CMPK2 can be induced in porcine IPEC-J2 cells through both IFN-I-dependent and independent pathways, which may be the reason for the inconsistent expression levels of CMPK2 in these 2 cells. Previous report showed that CMPK2-mediated antiviral activity occurred in IFN-dependent and IFN-independent manners [18]; however, both the regulatory mechanism of CMPK2 expression and the effect of CMPK2 on CoV replication remain unclear. In the present study, we revealed that PEDV infection directly induces CMPK2 expression via activating IRF1 in partially independent of IFN-I manners and CMPK2 exerted antiviral activities toward multiple CoVs. Therefore, we propose a working model in which CMPK2 broadly represses CoV replication. More evidence suggested that IRF1 can be activated by MAVS upon RIG-I and MDA5 recognition of viral RNA [41,42]. In our study, we focused on the effecter part of the IFN pathway and did not examine further the upstream sensors. Based on the published studies on CoV sensing [43–45], we would expect that the MDA5-MAVS is responsible for the majority of TBK1 activation and IFN induction. Although CoVs like PEDV inhibit IFN-I production through multiple mechanisms [20,21], IRF1 directly induces CMPK2 expression partially independently of IFN-I pathway. Subsequently, CMPK2 localizes to the mitochondria and provides sufficient substrate for Viperin-catalyzed production of ddhCTP through catalyzing the phosphorylation of CDP to produce CTP. ddhCTP, as a chain terminator for RdRp, directly prevents the replication of viruses by competing with natural ribonucleotides (Fig 9). These results collectively reveal an important defense mechanism for the host to successful defend against RNA virus infections.

ddhCTP has been shown to be catalyzed by Viperin and to inhibit viral RNA replication by directly acting on RdRp [25], the essential component for RNA virus replication. Here, we demonstrated that CMPK2 restricted CoVs including PEDV, IBV, and PDCoV replication by promoting ddhCTP production, which also explains why CMPK2 inhibits PEDV replication at the viral genome replication stage. Since ddhCTP is not cell permeable, we used a ddhC prodrug called HLB-0532247 to yield ddhCTP in cells. HLB-0532247 can be metabolized to ddhCTP in Huh7 cells, but it has not been experimentally tested in other cell lines. Consistent with recent reports from Seifer and colleagues and Moeller and colleagues, we also found that CMPK2 and Viperin’s end product ddhCTP could not inhibit SARS-CoV-2 replication in MA104 cells even at high concentrations (5 mM). Therefore, the lack of antiviral activity of against SARS-CoV-2 could be due to (1) a lack of metabolism to ddhCTP in MA104 cells or (2) ddhCTP simply inactive against SARS-CoV-2. Although SARS-CoV-2 polymerase is sensitive to ddhCTP inhibition, the exonuclease nsp14 is able to excise the natural antiviral chain-terminating nucleotide [10,46] and mediates resistance to CMPK2 activity. In addition, the CoV polymerase complex consists of the nsp12 (RdRp) catalytic subunit and nsp7-nsp8 cofactors, and the relationship between each subunit and CMPK2 is still not clear [7]. Although C-terminal including AKD is crucial for CMPK2 antiviral activity, the role of N-terminal in CMPK2 inhibiting CoVs replication process remains to be further explored.

Of note, PEDV infection induced high levels of CMPK2 expression in mitochondria, which provides the unique condition for CMPK2 catalyzing the phosphorylation of CDP to produce CTP. Mitochondria acts as a central hub in mediating host immune responses against foreign pathogen invasion [29]. Several studies suggested that CMPK2 is essential to mtDNA synthesis, which is crucial for NLRP3 inflammasome activation [16,47,48]. Furthermore, recent studies showed that metformin not only repressed NLRP3 activation by inhibiting ox-mtDNA production to relieve the inflammatory response, but also reduced the levels of OXPHOS and cellular energy supply via blocking mtDNA synthesis to inhibit HIV replication [49,50]. Therefore, versatile mechanisms of CMPK2 in innate immunity present itself as an effective antiviral target for future therapeutics development.

In summary, our present studies demonstrate that CMPK2 is directly induced by IRF1 after PEDV infection and exhibits significant antiviral activity to PEDV replication. ddhCTP catalysis by Viperin is essential for CMPK2 inhibiting PEDV replication, and the AKD of CMPK2 plays an important role in this process. CMPK2 also suppresses PDCoV replication through inhibiting the RdRp activity. Our findings collectively demonstrated a novel potential target for host antiviral immune response against CoV infections and provided mechanistic insight into the CoV–host interactions.

## Materials and methods

### Cell culture

Vero, HEK293T, DF-1, and LLC-PK1 cells were maintained in our laboratory and cultured in Dulbecco’s Modified Eagle Medium (DMEM; Gibco, Life Technologies, USA) supplemented with 10% heat-inactivated fetal bovine serum (FBS, Invitrogen, USA), whereas IPEC-J2 cells originally obtained from Creative Bioarray (New York, USA) and CMPK2#KO IPEC-J2 cells constructed by Jujiao (Nanchang, China) were cultured in complete advanced DMEM/F12 medium supplemented with 10% heat-inactivated FBS (Invitrogen, USA). MA104 cells were cultured in Medium 199 (Gibco, Life Technologies, USA) supplemented with 10% heat-inactivated FBS (Biological Industries, Israel). All media were supplemented with antibiotics (100 units/ml of penicillin, 100 μg /ml of streptomycin, and 0.25 μg /mL of amphotericin B; Thermo Fisher Scientific) and cultured in an incubator at 37 °C under an atmosphere with 5% CO_2_.

### Viruses and virus infection

PEDV strain AH2012/12 (GenBank: KU646831), PEDV strain JS2008 (GenBank: KC109141), IBV strain (CZ1603, maintained in our laboratory), and PDCoV strain CZ2020 (GenBank: OK546242) isolated and maintained in our laboratory. Vero or IPEC-J2 cells used to propagate PEDV, DF-1 cells used to propagate IBV, and LLC-PK1 cells used to propagate PDCoV were maintained in DMEM containing 7.5 μg/mL trypsin, respectively. When obviously cytopathic effects were observed, the infected cell cultures were freeze-thawed, and cell debris was removed by centrifugation at 4,000 × *g* for 5 min at 4 °C. The supernatant was collected and stored at −80 °C. Viral TCID_50_ were determined by limiting dilution when used.

Piglets were inoculated with PEDV (AH2012/12, 10^5^ TCID_50_/mL) and dissected at 48 hpi followed by extraction of tissues RNA. Vero, IPEC-J2, DF-1, and LLC-PK1 cells were seeded on 12-well plates (5 × 10^5^ cells per well) and infected with PEDV, IBV, or PDCoV for 1 h respectively, in the present of 7.5 μg/mL trypsin when grown to 100% confluence.

### Antibodies and reagents

Anti-CMPK2 ployclonal antibody, anti-Viperin, anti-PEDV *N*, anti-IBV *N*, and anti-PDCoV *N* monoclonal antibodies were prepared in our laboratory. Antibodies to anti-β-actin (ab8226), anti-pTKB1 (ab109272), anti-TBK1 (ab227182), anti-JAK1 (ab125051), and anti-JAK2 (ab245303) were obtained from Abcam. Anti-pSTAT1 (AF5935) and anti-STAT1 (AF0288) antibodies were purchased from Beyotime. Rabbit Control IgG (AC005) and anti-IRF1 antibody (A7692) were purchased from ABclonal. Goat Anti-Chicken IgG/FITC was purchased from Bioss. FITC Conjugated AffiniPure Goat Anti-mouse IgG (H+L) (BA1101) was purchased from BOSTER. Antibodies to anti-flag (80010-1-RR) and anti-HA (51064-2-AP) were purchased from Proteintech. Specific inhibitors of TBK1 (GSK8612, HY-111941), STAT1 (Fludarabine, HYB0069), and JAK1/2 (Ruxolitinib, HY50856) were obtained from MCE. NTPs (R0481) were purchased from Sangon Biotech (Shanghai, China). siRNAs to CMPK2, Viperin, and IRF1 were generated by Ribobio (Guangzhou, China), and the sequences were listed in S2 Table.

### Cell viability assay

A cell viability assay was performed using the Cell Counting Kit-8 (CCK-8) (Beyotime) according to the manufacturer’s instructions. Results were expressed relative to those for control cells, defined as 100% viability.

### Plasmids and transfection

The plasmids used in the present study were constructed as follows. The HA-tagged full-length CMPK2, CMPK2 mutation plasmid (CMPK2-ΔAKD and CMPK2-D330A), as well as flag-tagged full-length IRF1, NF-κB1, STAT2, and STAT5b, were constructed into pcDNA3.1 (+)-HA and pcDNA3.1 (+)-flag eukaryotic expression vectors, respectively. The full-length CMPK2 promoter, CMPK2 promoter truncation plasmids (M1, M2, and M3) were constructed into pGL3-Basic vector, respectively. Transient cell transfection was performed using the Lipofectamine 3000 reagent.

### Luciferase reporter assay

HEK293T cells were seeded on 12-well culture plates and transfected with plasmids using Lipofectamine 3000, according to the manufacturer’s protocol. Cells were cotransfected with a luciferase reporter plasmid (encoding firefly luciferase) containing the full CMPK2 promoter or a truncated promoter, together with the pRL-TK plasmid (encoding Renilla luciferase, as the internal reference control) (Fig 3A). To identify the CMPK2 activators (Fig 3D), cells were cotransfected with plasmid encoding IRF1, NF-κB1, STAT2, or STAT5b, the luciferase reporter plasmid (containing the CMPK2 promoter), and the pRL-TK plasmid. The cells were collected in 24 h after transfection, and their fluorescence was measured with the Dual Luciferase Reporter Assay System (Promega, E1910), according to the manufacturers’ protocol.

### Western blot

Cells were lysed with RIPA buffer (150 mM NaCl, 1% NP-40, 0.5% sodium deoxycholate, 0.1% SDS, 25 mM Tris with protease inhibitor cocktail (Roche)) and denatured for 5 min in 5 × SDS-PAGE loading buffer. Concentrations of the proteins were determined using the Bradford assay (Bio-Rad) and separated with SDS-PAGE and then transferred to nitrocellulose western blotting membranes (GE Healthcare, 10600001). The membranes were blocked in 5% skim milk powder in Tris-buffered saline-Tween (TBS-T) for 1.5 h at 37 °C, washed with PBS containing 1% Tween-20 (PBS-T), and then incubated with the primary antibody overnight at 4 °C. After washing, the membranes were incubated with the corresponding secondary antibody (conjugated with HRP) at room temperature for 1.5 h and detected with enhanced chemiluminescence (ECL) (Thermo Fisher Scientific, 34580).

### qRT-PCR

Total RNA was extracted from treated cells with the FastPure Cell/Tissue Total RNA Isolation Kit (Vazyme, RC112, Nanjing, China) and was reverse transcribed to cDNA using HiScript II Q RT SuperMix (Vazyme, R223-01) according to the manufacturer’s instructions. ChamQ Universal SYBR qPCR Master Mix (Vazyme, Q711-02) was used for real-time PCR. The reactions were run on QuantStudio 6 Flex machine (ABI, Thermo Fisher, USA) with the following steps: (i) 30 s at 95 °C and (ii) 40 cycles of 5 s at 95 °C and 34 s at 60 °C, followed by melting curve analysis. The primers used for the qPCR analysis are listed in S2 Table. Levels of mRNA were normalized to GAPDH using the formula 2^CT(GAPDH)-2 CT(mRNA ×)^ [51].

### Detection of ddhCTP in cell lysates

IPEC-J2 cells transfected with plasmids or corresponding siRNAs were collected and disrupted mechanically using a FastPrep-24 bead-beater device (MP) (2 cycles of 40 s, 6 m s^−1^, at 4 °C). Cell lysates were then centrifuged at 12,000*g* for 12 min at 4 °C, and the supernatant was loaded onto a 3-kDa filter Amicon Ultra-0.5 centrifugal filter unit (Merck) and centrifuged at 14,000*g* for 20 min at 4 °C. The resulting flow-through, containing substances smaller than 3 kDa, was used as the lysate sample for evaluating the presence of ddhCTP by LC–MS. LC–MS measurements were performed with a Waters Xevo TQ-s mass spectrometry system equipped with a ACQUITY UPLC H-Class system. Prior analysis, 10 μL of sample from enzymatic assays were mixed with 40 μL of acetonitrile: methanol organic mixture (5:3 v/v ratio). The mixtures were vortexed, centrifuged at 17,000*g* for 2 min and 3 μL of supernatant were injected onto an (XBridge Hilic 3.5 μm ploymeric 2.1 × 150 mm) HPLC column. The mobile phase was composed of 0.1% formic acid water (solvent A) and 100% acetonitrile (solvent B). Samples were separated using a constant flow rate of 0.3 mL/min: 95% solvent B was held for 10 min, followed by a gradient from 95% to 50% of solvent B for 5 min, before immediately returning to 95% solvent B for equilibration for 10 min. Instrument control, data processing, and data analysis were performed using the MassLynx V4.2 software.

### Cell-based virus RdRp activity assay

HEK293T cells seeded on 24-well plates were transfected with corresponding plasmids and treated with or without gradient concentrations of NTP and then transfected with the CoVs RdRp activity assay system. After 20 h, the FLuc and NLuc values of these cells were measured using the Nano-Glo Dual Luciferase Reporter Assay System (Promega, E1910), and the NLuc values were normalized with FLuc.

### SARS-CoV-2 infection

MA104 cells were seeded in 96-well plates. When reaching 80% to 90% confluency, cells were pretreated with JIB-04 or ddhC (cell-permeable precursor of ddhCTP; [27]) at desired concentrations for 1 h, followed by 24-h infection of a clinical isolate of SARS-CoV-2 (2019-nCoV/USA-WA1/2020 strain) at MOI of 1 in BSL3 conditions. Intracellular RNA was harvested for qRT-PCR analysis to examine SARS-CoV-2 N mRNA levels as relative to GAPDH as previously described [52].

### Statistical analyses

Statistical analysis was performed using the GraphPad Prism 5 software (GraphPad Software, USA). All results are representative of 3 independent experiments. Data are presented as means ± standard deviations (SDs) and were analyzed with the two-tailed Student *t* test or one/two-way analysis of variance (ANOVA), followed by multiple-comparison correction. *P* values of <0.05 were considered statistically significant.

### Ethics

Animal experiments were performed with the approval of the Jiangsu Academy of Agricultural Sciences Experimental Animal Ethics Committee (**No.NKYVET 2015-DWLL-20210126**). Efforts were made to minimize animal suffering and reduce the number of animals used.

## Supporting information

S1 Fig Transcriptomics analysis of IPEC-J2 cells infected by PEDV. (**A**) A volcano plot of RNA-sequencing data from IPEC-J2 cells infected with PEDV infection (MOI = 1) at 20 hpi. (**B**) A heatmap with the abundance of mitochondria-related genes based on RNA-sequencing data. Data underlying this figure can be found in S1 Data. hpi, hours post-infection; MOI, multiplicity of infection; PEDV, porcine epidemic diarrhea virus.
(TIF)

S2 Fig The standard curve of PEDV quantitative detection. Standard curves were established using serially diluted target plasmids based on the purified PEDV genomic RNAs. Data underlying this figure can be found in S1 Data.
(TIF)

S3 Fig Both the virulent strain AH2012/12 and the attenuated strain JS2008 of PEDV could induce CMPK2 expression. IPEC-J2 cells were infected with AH2012/12 or JS2008 at MOI = 1 for 20 h, and the expression of CMPK2 were detected by qRT-PCR (left) and western blot (right), respectively. Data are means ± SD of triplicate samples; an ordinary one-way ANOVA was performed followed by Dunnett’s multiple comparison test; only the *p*-value for the most relevant comparisons are shown for simplicity. The intensities of bands were quantified by ImageJ. ***p < 0.001. ns, no significance. Data underlying this figure can be found in S1 Data and S1 Raw Images. CMPK2, cytidine/uridine monophosphate kinase 2; MOI, multiplicity of infection; PEDV, porcine epidemic diarrhea virus; qRT-PCR, quantitative real-time PCR.
(TIF)

S4 Fig PEDV replication is essential to induce CMPK2 expression. PEDV (AH2012/12) with or without UV inactivation infected IPEC-J2 cells at MOI = 1 for 20 h, and the expression of CMPK2 were detected by qRT-PCR (left) and western blot (right), respectively. Data are means ± SD of triplicate samples; an ordinary one-way ANOVA was performed followed by Dunnett’s multiple comparison test; only the *p*-value for the most relevant comparisons are shown for simplicity. The intensities of bands were quantified by ImageJ. ****p < 0.0001. Data underlying this figure can be found in S1 Data and S1 Raw Images. CMPK2, cytidine/uridine monophosphate kinase 2; MOI, multiplicity of infection; PEDV, porcine epidemic diarrhea virus; qRT-PCR, quantitative real-time PCR; UV, ultraviolet.
(TIF)

S5 Fig Effects of different concentrations of GSK8612, Fludarabine, and Ruxolitinib on cell viability. IPEC-J2 cells were treated with DMSO or different concentrations of GSK8612 (**A**), Fludarabine (**B**), and Ruxolitinib (**C**) as indicated. Cell viability was determined by CCK-8 assay at 1 h, 4 h, and 16 h after treatment according to the manufacturer’s protocol. *n* = 3 with 3 technical repeats each time. Data underlying this figure can be found in S1 Data.
(TIF)

S6 Fig GSK8612, Fludarabine, and Ruxolitinib are highly effective inhibitors of IFN-I pathway. qRT-PCR analysis of classical ISGs such as CXCL10, IFIT1, and MX1 mRNA levels in IPEC-J2 cells infected with PEDV (MOI = 1) followed by treatment with GSK8612 (**A**, 2 μM), Fludarabine (**B**, 7 μM), or Ruxolitinib (**C**, 9 μM). Data are means ± SD of triplicate samples; statistical analysis was conducted using two-way ANOVA followed by Tukey’s multiple comparison test; only the *p*-value for the most relevant comparisons are shown for simplicity. ****p < 0.0001. ns, no significance. Data underlying this figure can be found in S1 Data. IFN-I, type I interferon; ISG, interferon-stimulated gene; MOI, multiplicity of infection; PEDV, porcine epidemic diarrhea virus; qRT-PCR, quantitative real-time PCR.
(TIF)

S7 Fig The CMPK2 promoter showed excellent IFN responsiveness. (**A**) IPEC-J2 cells were treated with DMSO or different concentrations of IFN-α1 as indicated. The expression of CMPK2 were detected by qRT-PCR (up) and western blot (down), respectively. The intensities of bands were quantified by ImageJ. (**B**) IPEC-J2 cells were transfected with CMPK2 promoter-driven luciferase vector, pRL-TK-luc vector, and treated with different concentrations of IFN-α1 as indicated. Samples were collected at 20 h post-transfection and analyzed for dual luciferase activity. Data are means ± SD of triplicate samples; statistical analysis was conducted using one-way ANOVA followed by Dunnett’s multiple comparison test or two-way ANOVA followed by Tukey’s multiple comparison test; only the *p*-value for the most relevant comparisons are shown for simplicity. *p < 0.05, ***p < 0.001, ****p < 0.0001. Data underlying this figure can be found in S1 Data and S1 Raw Images. CMPK2, cytidine/uridine monophosphate kinase 2; IFN, interferon; qRT-PCR, quantitative real-time PCR.
(TIF)

S8 Fig IRF1 interacts with the CMPK2 promoter. IPEC-J2 cells were transfected with Flag-IRF1-encoding plasmid or empty vector, and the cells were harvested at 20 h post-transfection and processed for Co-IP and ChIP analysis. Chromatin-bound IRF1 was precipitated with an anti-Flag antibody or normal rabbit IgG. The CMPK2 promoter sequences were detected by qRT-PCR. Data are means ± SD of triplicate samples; statistical analysis was conducted using two-way ANOVA followed by Tukey’s multiple comparison test. ****p < 0.0001. Data underlying this figure can be found in S1 Data and S1 Raw Images. ChIP, chromatin immunoprecipitation; CMPK2, cytidine/uridine monophosphate kinase 2; Co-IP, co-immunoprecipitation; IRF1, interferon regulatory factor 1; qRT-PCR, quantitative real-time PCR; WCL, whole cell lysate.
(TIF)

S9 Fig CMPK2 inhibits PEDV replication in Vero cells. (**A** and **B**) Vero cells were transfected with empty vector (pEmpty) and pCMPK2 for 24 h and then infected with PEDV (MOI = 1). Total RNA of the cells harvested at 20 hpi were extracted and analyzed by qRT-PCR (**A**), and the cell lysates were analyzed by western blotting (**B**). (**C**) Culture supernatants were collected at 8 hpi and 20 hpi, respectively. PEDV titers in the culture supernatants were measured as TCID_50_. (**D** and **E**) Vero cells were transfected with CMPK2 siRNA (#1, #2, and #3) and negative control (siCMPK2#NC) for 12 h and then infected with PEDV (MOI = 1). Total RNA of the cells harvested at 20 hpi were extracted and analyzed by qRT-PCR, and the cell lysates were analyzed by western blotting. (**F**) Culture supernatants were collected at 8 hpi and 20 hpi, respectively. PEDV titers in the culture supernatants were measured as TCID_50_. Data are means ± SD of triplicate samples; statistical analysis was conducted using one-way ANOVA followed by Dunnett’s multiple comparison test or two-way ANOVA followed by Tukey’s multiple comparison test; only the *p*-value for the most relevant comparisons are shown for simplicity. The intensities of bands were quantified by ImageJ. **p < 0.01, ***p < 0.001, ****p < 0.0001. Data underlying this figure can be found in S1 Data and S1 Raw Images. CMPK2, cytidine/uridine monophosphate kinase 2; hpi, hours post-infection; MOI, multiplicity of infection; PEDV, porcine epidemic diarrhea virus; qRT-PCR, quantitative real-time PCR; siRNA, small interfering RNA.
(TIF)

S10 Fig CMPK2 inhibits PEDV replication intracellularly. (**A**) Overview of the experimental design to examine virus binding and entry. (**B** and **C**) IPEC-J2 cells were transfected with empty vector (pEmpty) and pCMPK2 for 24 h and then infected with PEDV at MOI of 5. The cells were harvested at indicate time according to A. Total RNA of the cells was extracted, and viral copy numbers were analyzed by qRT-PCR. (D) The cell lysates harvested as above were analyzed by western blotting. Data are means ± SD of triplicate samples; statistical analysis was conducted using two-way ANOVA followed by Tukey’s multiple comparison test; only the *p*-value for the most relevant comparisons are shown for simplicity. The intensities of bands were quantified by ImageJ. ***p < 0.001. ns, no significance. Data underlying this figure can be found in S1 Data and S1 Raw Images. CMPK2, cytidine/uridine monophosphate kinase 2; MOI, multiplicity of infection; PEDV, porcine epidemic diarrhea virus; qRT-PCR, quantitative real-time PCR.
(TIF)

S11 Fig Knockdown of Viperin reduced ddhCTP production and decreased CMPK2 antiviral activity. (**A**) The knockdown efficiency of Viperin. IPEC-J2 cells were transfected with Viperin siRNA (#1, #2, and #3) or negative control (siViperin#NC) for 12 h. Viperin expression level was analyzed by qRT-PCR and western blotting. β-actin was used as the sample loading control. (**B**) IPEC-J2 cells with or without CMPK2 expression were transfected with siViperin#1 as well as the siViperin#NC and then infected with PEDV at MOI of 1. The cells were harvested at 20 hpi, and the lysates were analyzed by western blotting. (**C**) IPEC-J2 cells were transfected with siViperin#1 and siViperin#NC for 12 h, and ddhCTP production was detected by LC–MS. Data are means ± SD of triplicate samples; statistical analysis was conducted using one-way ANOVA followed by Dunnett’s multiple comparison test; only the *p*-value for the most relevant comparisons are shown for simplicity. The intensities of bands were quantified by ImageJ. *p < 0.05. Data underlying this figure can be found in S1 Data and S1 Raw Images. CMPK2, cytidine/uridine monophosphate kinase 2; ddhCTP, 3′-deoxy-3′,4′-didehydro-cytidine triphosphate; hpi, hours post-infection; LC–MS, liquid chromatography followed by mass spectrometry; MOI, multiplicity of infection; PEDV, porcine epidemic diarrhea virus; qRT-PCR, quantitative real-time PCR; siRNA, small interfering RNA.
(TIF)

S12 Fig The CMPK2 catalytic site identified before is not highly conserved. (**A**) Phylogenetic analysis of full-length CMPK2 was shown with cladogram. (**B**) The comparison of CMPK2 catalytic site sequences between different species. Gray indicates the comparison of identified poultry CMPK2 catalytic sites across multiple species. Blue indicates different amino acids of CMPK2 among the above species. The highly conserved aspartate (D) residue are labeled in red triangle. (**C**-**D**) The actual catalytic mutant of CMPK2 (CMPK2-D330A-HA) was constructed, and IPEC-J2 cells were transfected with CMPK2-D330A-HA, CMPK2-HA, and empty vector respectively followed by PEDV infection for 20 hpi. The cell lysates were analyzed by western blotting (**C**), and total RNA of the cells were extracted and analyzed by qRT-PCR (**D**). (**E**) Culture supernatants were collected at 20 hpi, and PEDV titers were measured as TCID_50_. Data are means ± SD of triplicate samples; statistical analysis was conducted using one-way ANOVA followed by Dunnett’s multiple comparison; only the *p*-value for the most relevant comparisons are shown for simplicity. The intensities of bands were quantified by ImageJ. **p < 0.01. ****p < 0.0001. Data underlying this figure can be found in S1 Data and S1 Raw Images. CMPK2, cytidine/uridine monophosphate kinase 2; hpi, hours post-infection; PEDV, porcine epidemic diarrhea virus; qRT-PCR, quantitative real-time PCR.
(TIF)

S13 Fig AKD of CMPK2 is crucial for ddhCTP production. IPEC-J2 cells were transfected with AKD deletion mutant (pCMPK2-ΔAKD-HA) and pCMPK2-HA, respectively, and then ddhCTP production was detected by LC–MS. Data are means ± SD of triplicate samples; statistical analysis was conducted using one-way ANOVA followed by Dunnett’s multiple comparison; only the *p*-value for the most relevant comparisons is shown for simplicity. ns, no significance. Data underlying this figure can be found in S1 Data and S1 Raw Images. AKD, antiviral key domain; CMPK2, cytidine/uridine monophosphate kinase 2; ddhCTP, 3′-deoxy-3′,4′-didehydro-cytidine triphosphate; LC–MS, liquid chromatography followed by mass spectrometry.
(TIF)

S14 Fig AKD is the essential domain of CMPK2 affecting CoV RdRp activity. HEK293T cells were transfected with pCMPK2-ΔAKD, pCMPK2, and empty vector, respectively, and then RdRp activity of PEDV (**A**), IBV (**B**), PDCoV (**C**), and SARS-CoV-2 (**D**) were detected by the cell-based reporter assay system, respectively. Data are means ± SD of triplicate samples; statistical analysis was conducted using one-way ANOVA followed by Dunnett’s multiple comparison; only the *p*-value for the most relevant comparisons are shown for simplicity. ***p < 0.001. Data underlying this figure can be found in S1 Data. AKD, antiviral key domain; CMPK2, cytidine/uridine monophosphate kinase 2; CoV, coronavirus; IBV, infectious bronchitis virus; PDCoV, porcine delta-coronavirus; PEDV, porcine epidemic diarrhea virus; RdRp, RNA-dependent RNA polymerase; SARS-CoV-2, Severe Acute Respiratory Syndrome Coronavirus 2.
(TIF)

S1 Table Nucleotide sequence of the porcine CMPK2 promoter. (DOCX)

S2 Table Primer, siRNA, and sgRNA sequences used in this study. (DOCX)

S1 Data Excel spreadsheet containing, in separate sheets, the underlying numerical data for Figs 1A–1D, 2A–2E, 3A, 3C–3F, 4A, 4C, 4E, 4F, 5A–5E, 5G, 6C–6E, 6G, 6H, 7B–7E, 8B, 8C and 8E–8G and S1A, S1B, S2, S3, S4, S5A–S5C, S6A–S6C, S7A, S7B, S8, S9A, S9C, S9D, S9F, S10B, S10C, S11A, S11C, S12D, S12E, S13 and S14A–S14D. (XLSX)

S1 Raw images Raw western blot data. (PDF)

## Abbreviations

AKD: antiviral key domain
ChIP: chromatin immunoprecipitation
CMPK2: cytidine/uridine monophosphate kinase 2
CoV: coronavirus
ddhCTP: 3′-deoxy-3′,4′-didehydro-cytidine triphosphate
DENV: dengue virus
DMEM: Dulbecco’s Modified Eagle Medium
ECL: enhanced chemiluminescence
FBS: fetal bovine serum
HIV: human immunodeficiency virus
hpi: hours post-infection
IBV: infectious bronchitis virus
IFA: immunofluorescent assay
IFN-I: type I interferon
IRF: interferon regulatory factor
IRF1: interferon regulatory factor 1
ISG: interferon-stimulated gene
LC–MS: liquid chromatography followed by mass spectrometry
LLC-PK1: Lilly Laboratories cell porcine kidney 1
MERS-CoV: Middle East Respiratory Syndrome Coronavirus
MOI: multiplicity of infection
mtDNA: mitochondrial DNA
NTP: nucleotide triphosphate
PBS: phosphate-buffered saline
PDCoV: porcine delta-coronavirus
PEDV: porcine epidemic diarrhea virus
qRT-PCR: quantitative real-time PCR
RdRp: RNA-dependent RNA polymerase
SARS-CoV: Severe Acute Respiratory Syndrome Coronavirus
siRNA: small interfering RNA
SVCV: spring viremia of carp virus
TFBS: transcription factor binding site
