# Supplementary figures and images for: CMPK2 is a host restriction factor that inhibits infection of multiple coronaviruses in a cell-intrinsic manner
Source: PLoS Biol. 2023 Mar 17;21(3):e3002039. doi: 10.1371/journal.pbio.3002039 (PMC10058120; doi:10.1371/journal.pbio.3002039)

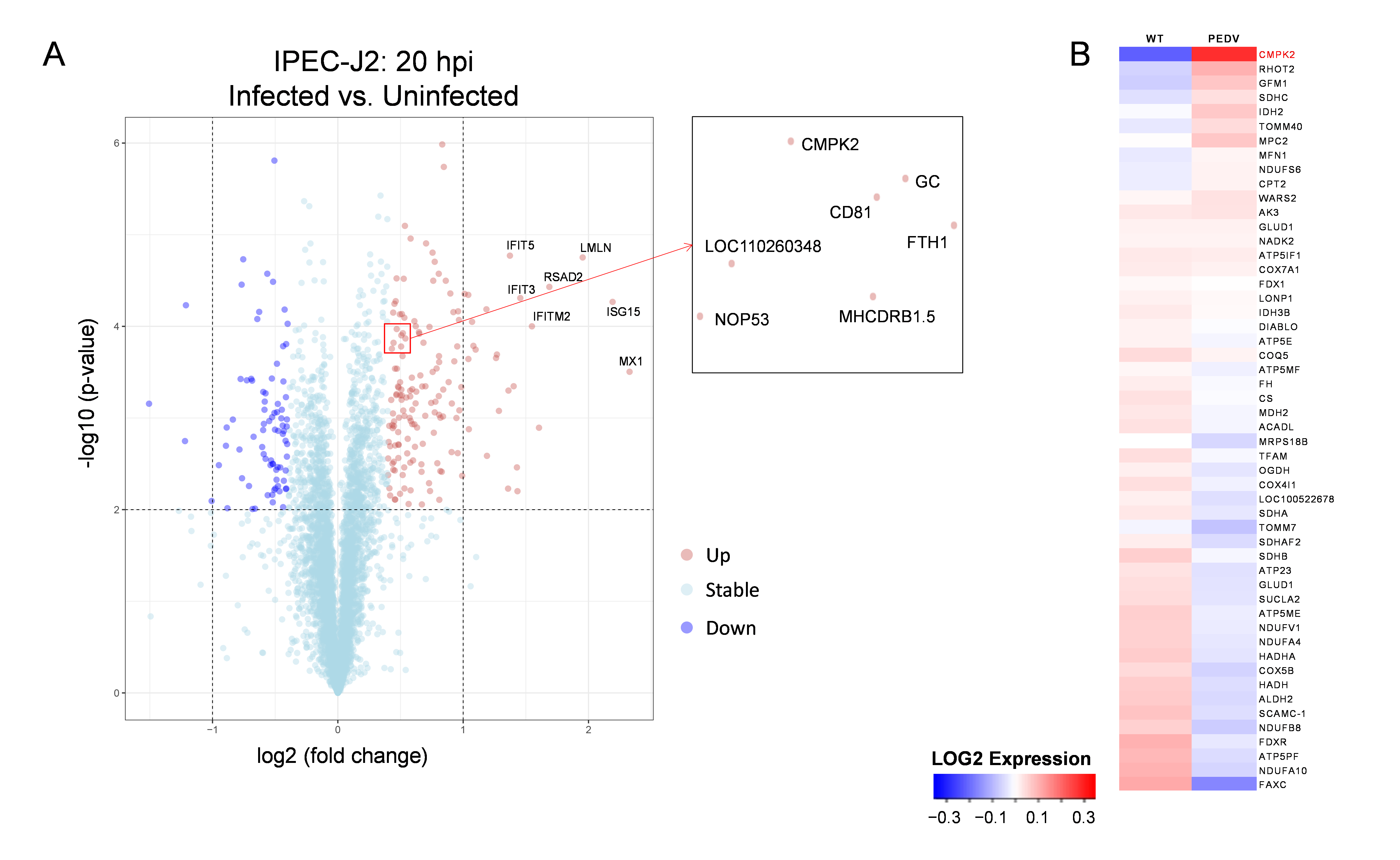

Supplement: S1 Fig — (A) A volcano plot of RNA-sequencing data from IPEC-J2 cells infected with PEDV infection (MOI = 1) at 20 hpi. (B) A heatmap with the abundance of mitochondria-related genes based on RNA-sequencing data. Data underlying this figure can be found in S1 Data. hpi, hours post-infection; MOI, multiplicity of infection; PEDV, porcine epidemic diarrhea virus. (TIF) [file pbio.3002039.s001.tif]

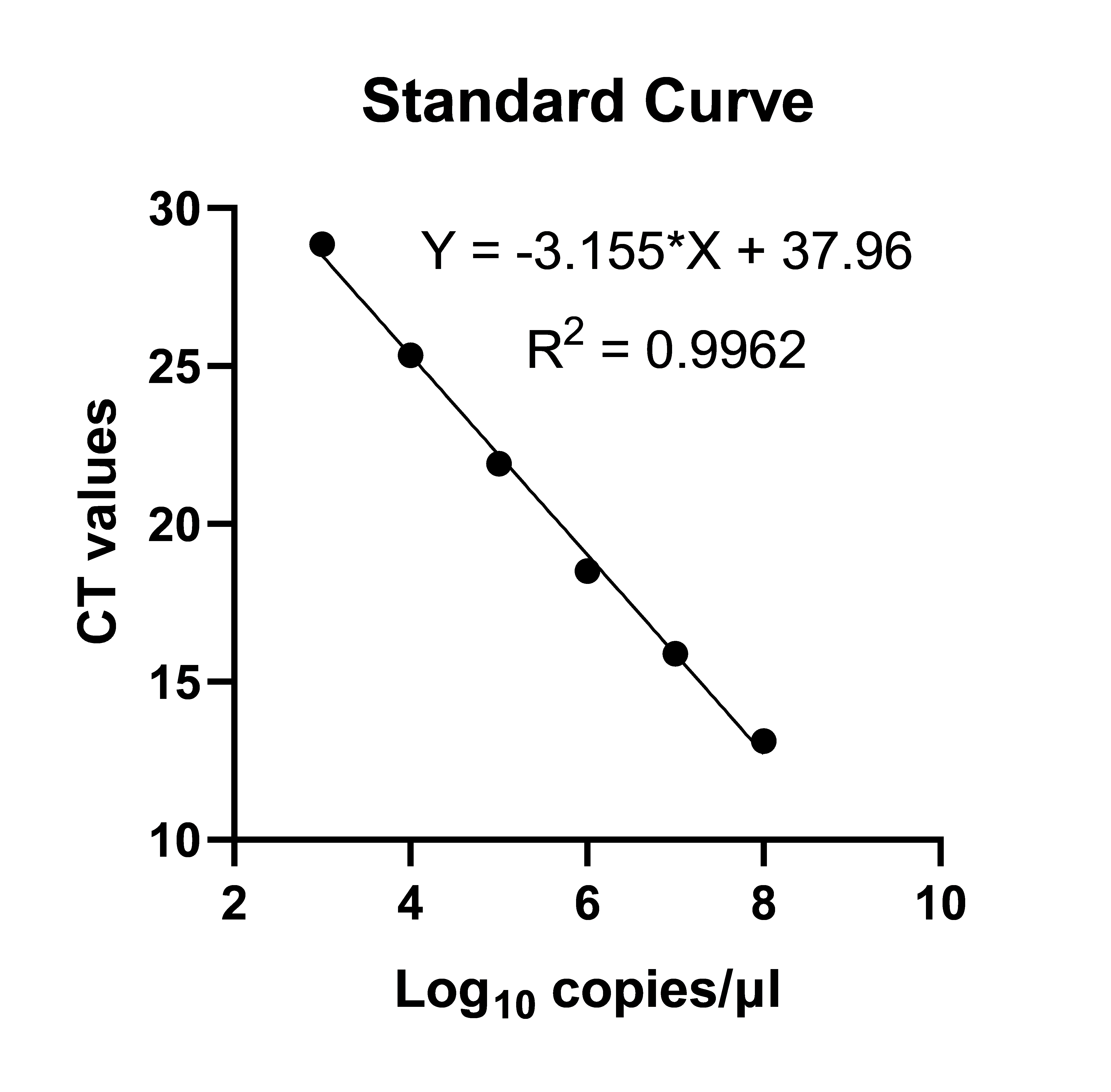

Supplement: S2 Fig — Standard curves were established using serially diluted target plasmids based on the purified PEDV genomic RNAs. Data underlying this figure can be found in S1 Data. (TIF) [file pbio.3002039.s002.tif]

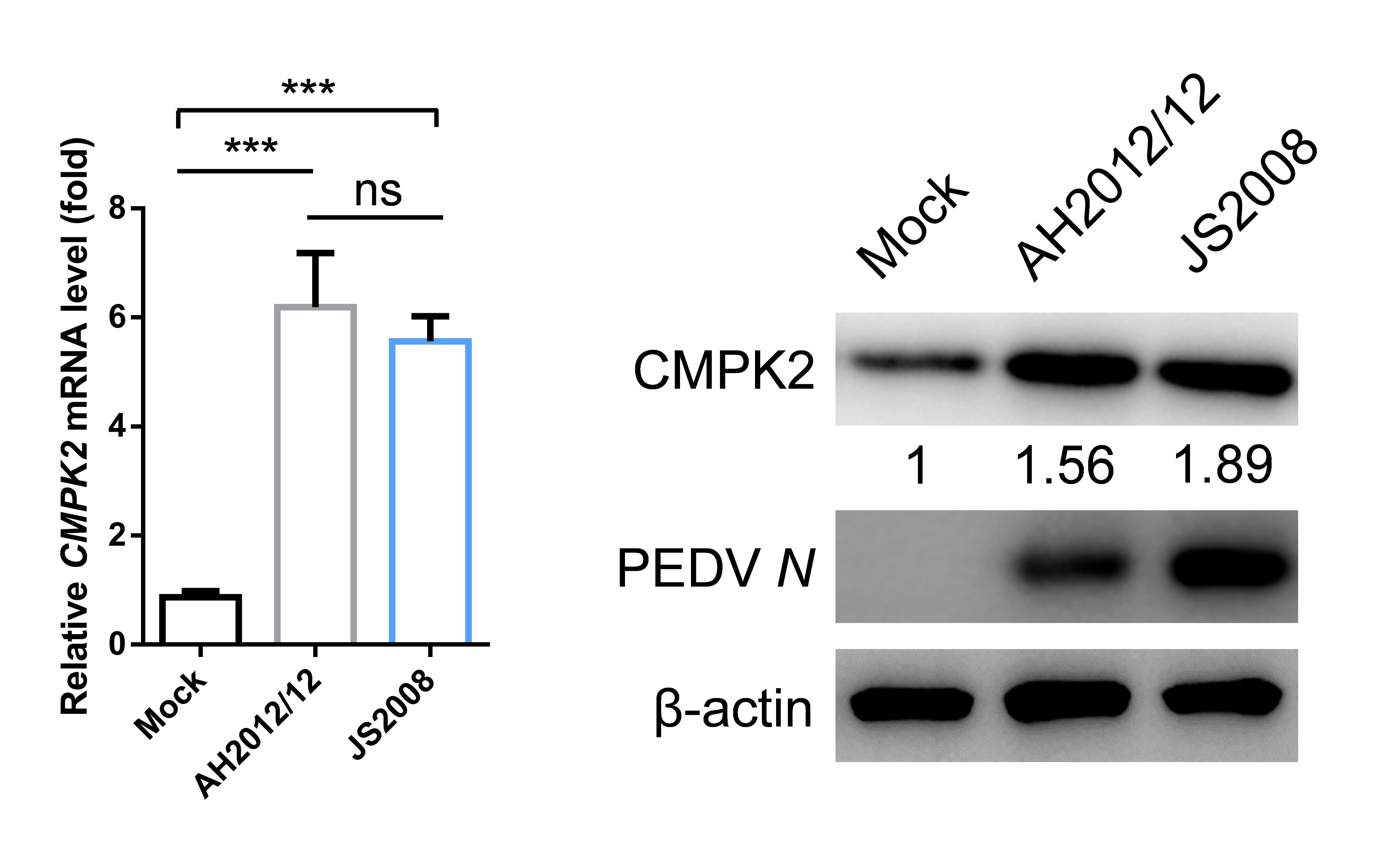

Supplement: S3 Fig — IPEC-J2 cells were infected with AH2012/12 or JS2008 at MOI = 1 for 20 h, and the expression of CMPK2 were detected by qRT-PCR (left) and western blot (right), respectively. Data are means ± SD of triplicate samples; an ordinary one-way ANOVA was performed followed by Dunnett’s multiple comparison test; only the p-value for the most relevant comparisons are shown for simplicity. The intensities of bands were quantified by ImageJ. ***p < 0.001. ns, no significance. Data underlying this figure can be found in S1 Data and S1 Raw Images. CMPK2, cytidine/uridine monophosphate kinase 2; MOI, multiplicity of infection; PEDV, porcine epidemic diarrhea virus; qRT-PCR, quantitative real-time PCR. (TIF) [file pbio.3002039.s003.tif]

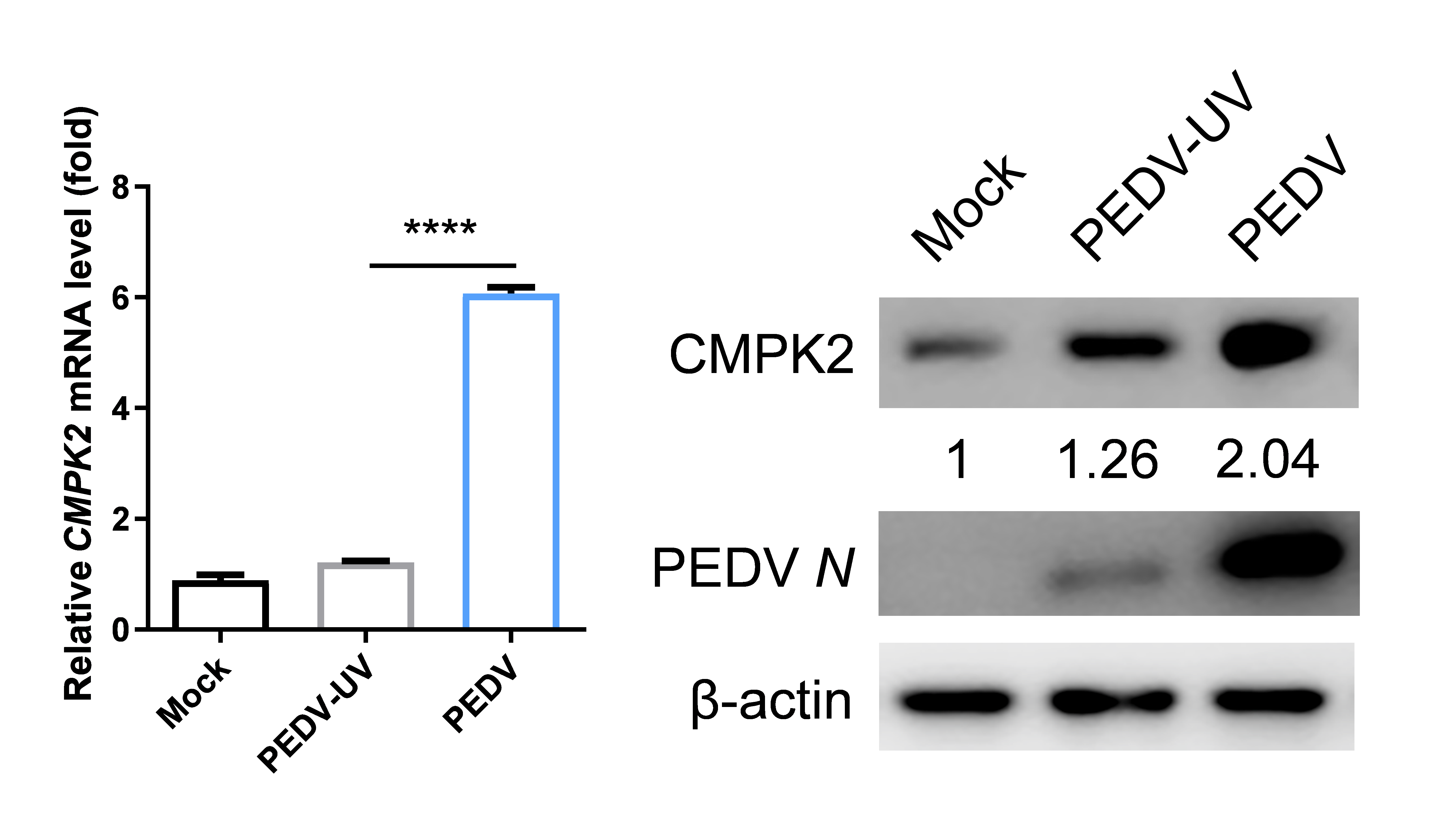

Supplement: S4 Fig — PEDV (AH2012/12) with or without UV inactivation infected IPEC-J2 cells at MOI = 1 for 20 h, and the expression of CMPK2 were detected by qRT-PCR (left) and western blot (right), respectively. Data are means ± SD of triplicate samples; an ordinary one-way ANOVA was performed followed by Dunnett’s multiple comparison test; only the p-value for the most relevant comparisons are shown for simplicity. The intensities of bands were quantified by ImageJ. ****p < 0.0001. Data underlying this figure can be found in S1 Data and S1 Raw Images. CMPK2, cytidine/uridine monophosphate kinase 2; MOI, multiplicity of infection; PEDV, porcine epidemic diarrhea virus; qRT-PCR, quantitative real-time PCR; UV, ultraviolet. (TIF) [file pbio.3002039.s004.tif]

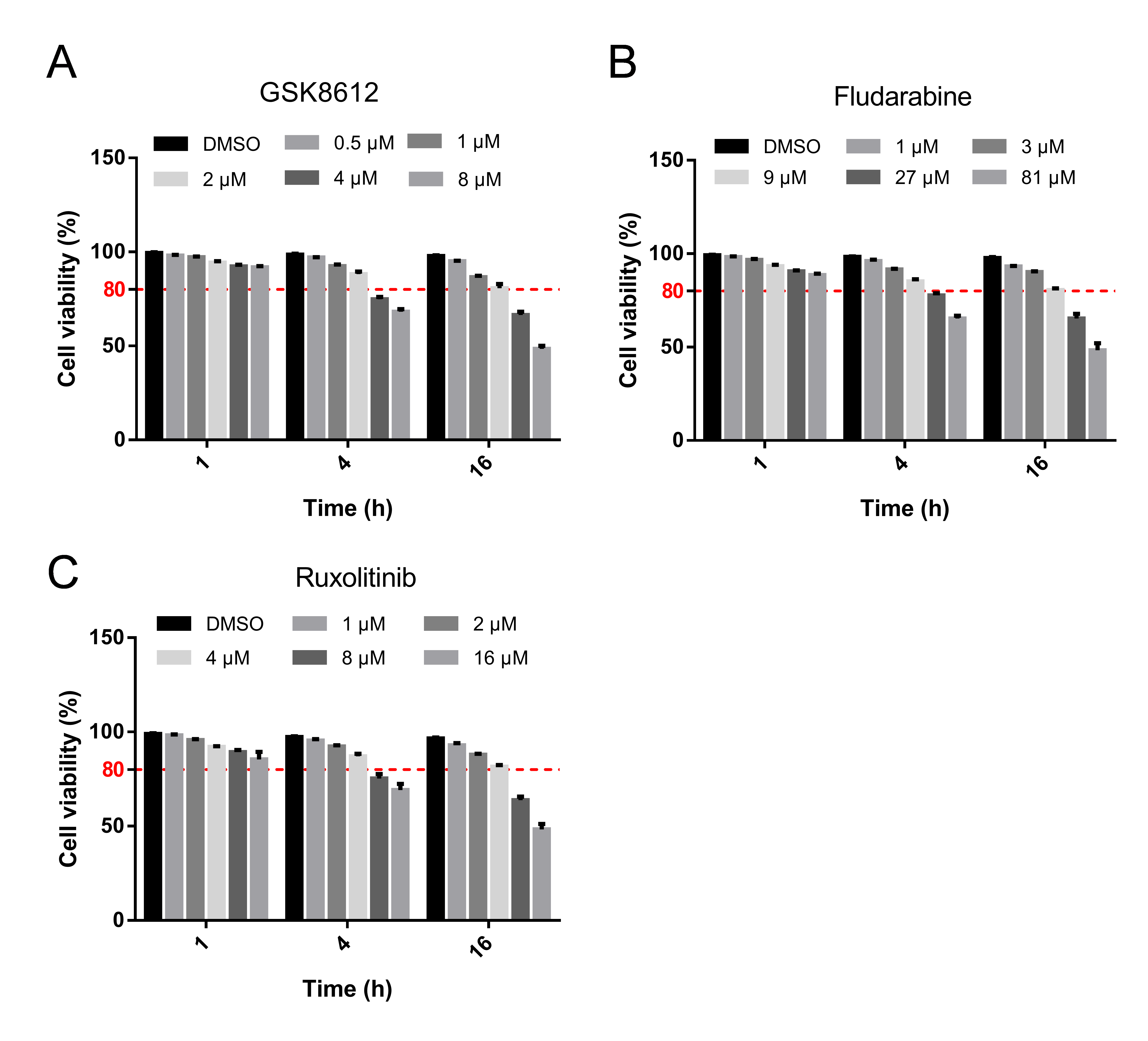

Supplement: S5 Fig — IPEC-J2 cells were treated with DMSO or different concentrations of GSK8612 (A), Fludarabine (B), and Ruxolitinib (C) as indicated. Cell viability was determined by CCK-8 assay at 1 h, 4 h, and 16 h after treatment according to the manufacturer’s protocol. n = 3 with 3 technical repeats each time. Data underlying this figure can be found in S1 Data. (TIF) [file pbio.3002039.s005.tif]

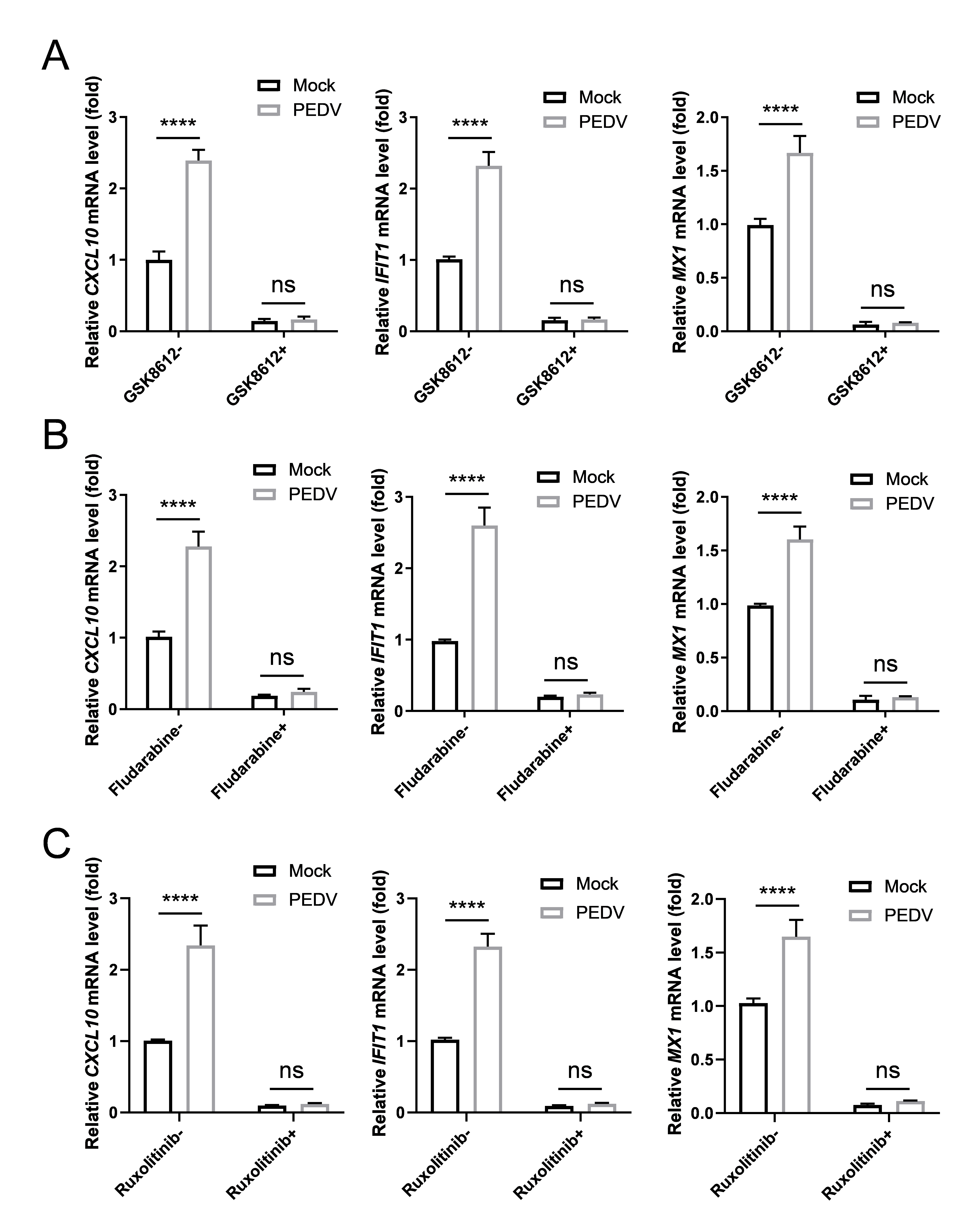

Supplement: S6 Fig — qRT-PCR analysis of classical ISGs such as CXCL10, IFIT1, and MX1 mRNA levels in IPEC-J2 cells infected with PEDV (MOI = 1) followed by treatment with GSK8612 (A, 2 μM), Fludarabine (B, 7 μM), or Ruxolitinib (C, 9 μM). Data are means ± SD of triplicate samples; statistical analysis was conducted using two-way ANOVA followed by Tukey’s multiple comparison test; only the p-value for the most relevant comparisons are shown for simplicity. ****p < 0.0001. ns, no significance. Data underlying this figure can be found in S1 Data. IFN-I, type I interferon; ISG, interferon-stimulated gene; MOI, multiplicity of infection; PEDV, porcine epidemic diarrhea virus; qRT-PCR, quantitative real-time PCR. (TIF) [file pbio.3002039.s006.tif]

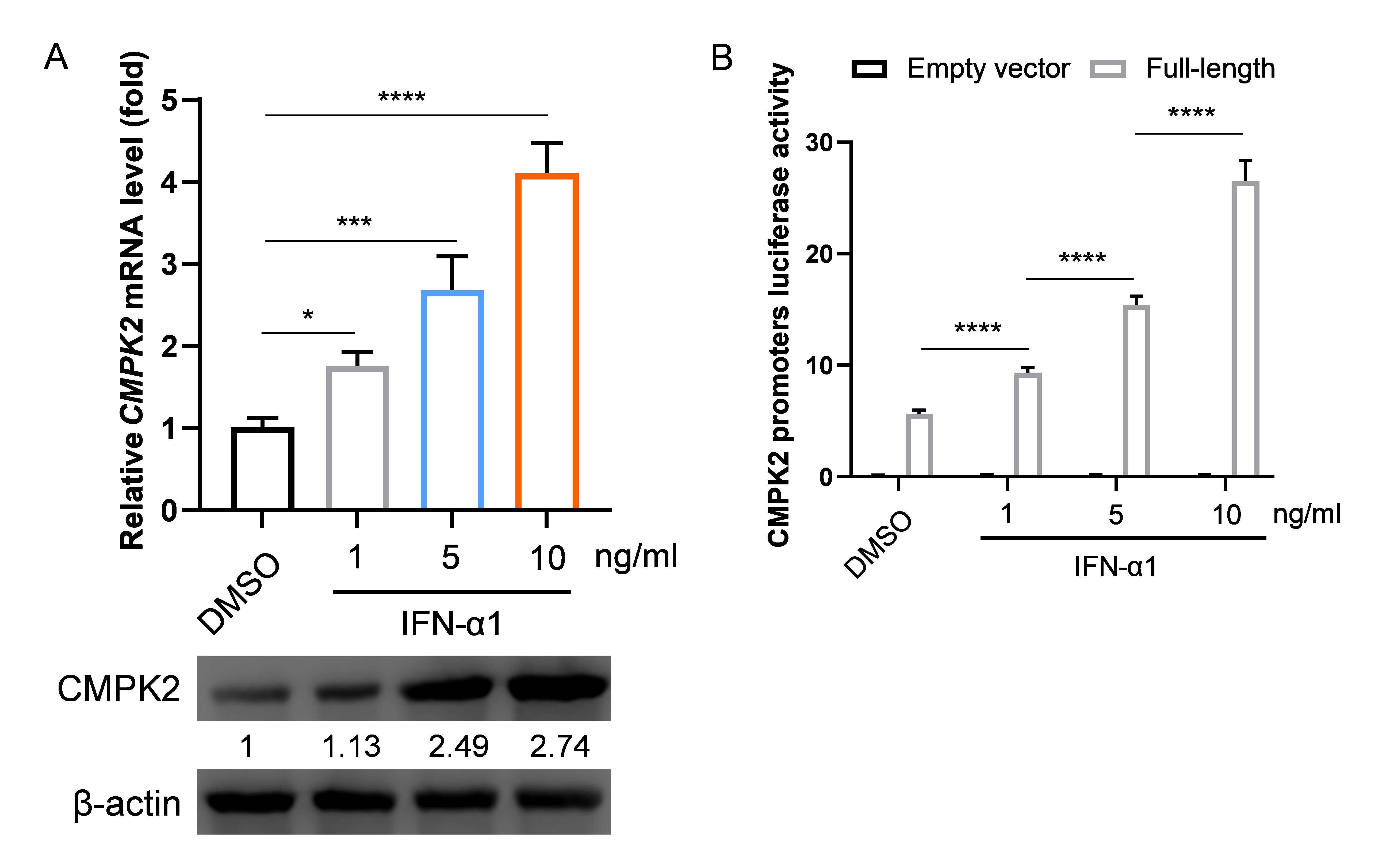

Supplement: S7 Fig — (A) IPEC-J2 cells were treated with DMSO or different concentrations of IFN-α1 as indicated. The expression of CMPK2 were detected by qRT-PCR (up) and western blot (down), respectively. The intensities of bands were quantified by ImageJ. (B) IPEC-J2 cells were transfected with CMPK2 promoter-driven luciferase vector, pRL-TK-luc vector, and treated with different concentrations of IFN-α1 as indicated. Samples were collected at 20 h post-transfection and analyzed for dual luciferase activity. Data are means ± SD of triplicate samples; statistical analysis was conducted using one-way ANOVA followed by Dunnett’s multiple comparison test or two-way ANOVA followed by Tukey’s multiple comparison test; only the p-value for the most relevant comparisons are shown for simplicity. *p < 0.05, ***p < 0.001, ****p < 0.0001. Data underlying this figure can be found in S1 Data and S1 Raw Images. CMPK2, cytidine/uridine monophosphate kinase 2; IFN, interferon; qRT-PCR, quantitative real-time PCR. (TIF) [file pbio.3002039.s007.tif]

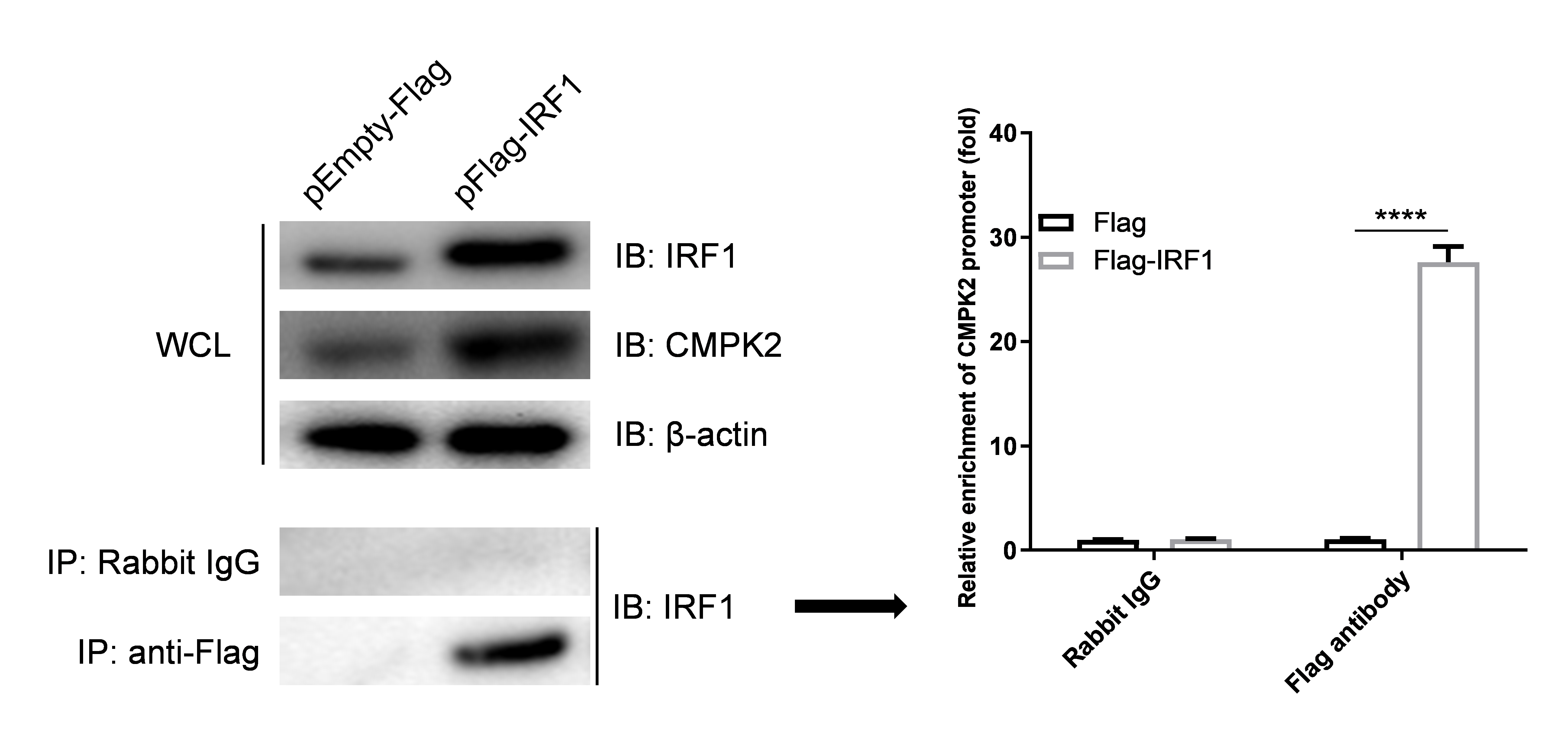

Supplement: S8 Fig — IPEC-J2 cells were transfected with Flag-IRF1-encoding plasmid or empty vector, and the cells were harvested at 20 h post-transfection and processed for Co-IP and ChIP analysis. Chromatin-bound IRF1 was precipitated with an anti-Flag antibody or normal rabbit IgG. The CMPK2 promoter sequences were detected by qRT-PCR. Data are means ± SD of triplicate samples; statistical analysis was conducted using two-way ANOVA followed by Tukey’s multiple comparison test. ****p < 0.0001. Data underlying this figure can be found in S1 Data and S1 Raw Images. ChIP, chromatin immunoprecipitation; CMPK2, cytidine/uridine monophosphate kinase 2; Co-IP, co-immunoprecipitation; IRF1, interferon regulatory factor 1; qRT-PCR, quantitative real-time PCR; WCL, whole cell lysate. (TIF) [file pbio.3002039.s008.tif]

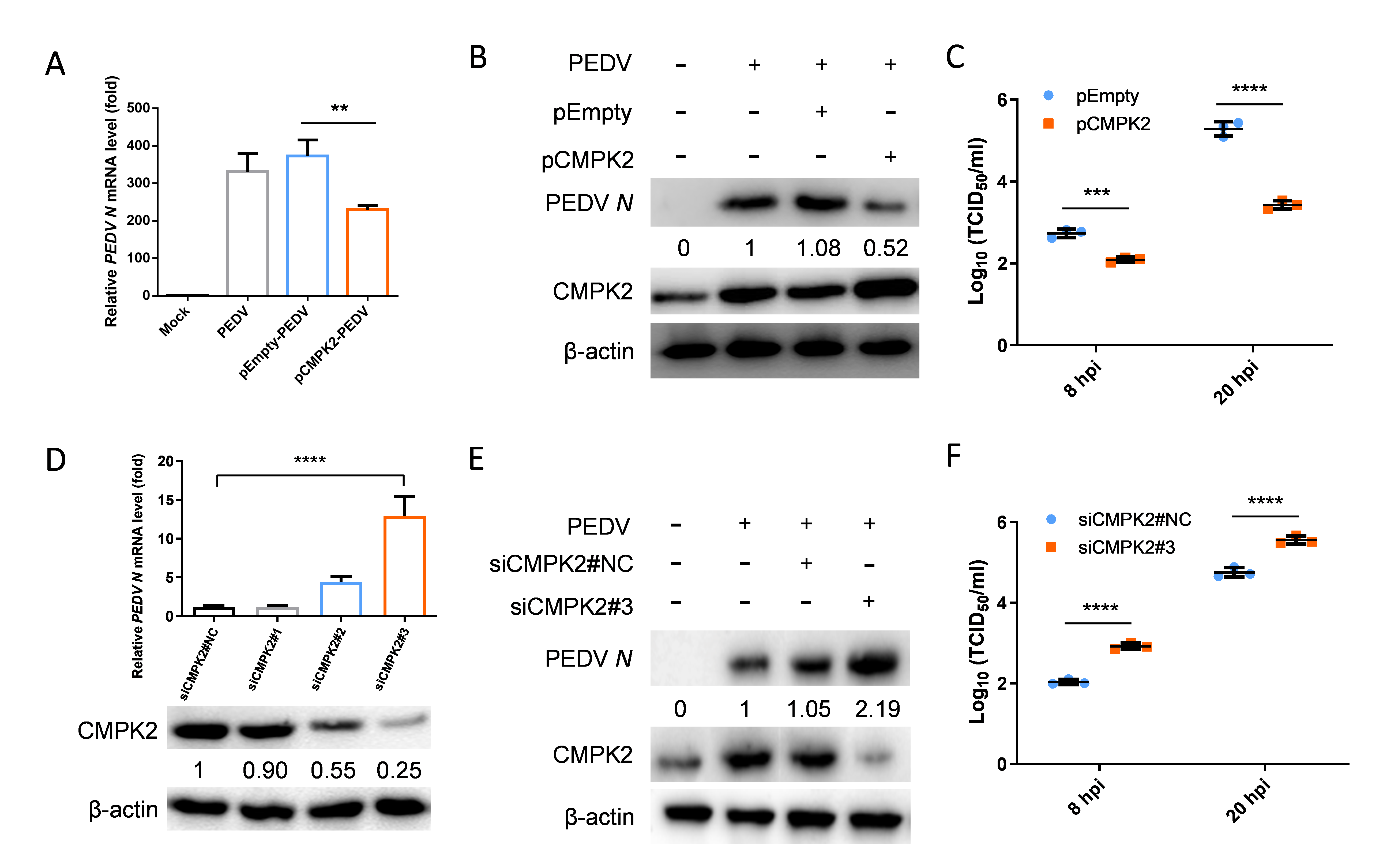

Supplement: S9 Fig — (A and B) Vero cells were transfected with empty vector (pEmpty) and pCMPK2 for 24 h and then infected with PEDV (MOI = 1). Total RNA of the cells harvested at 20 hpi were extracted and analyzed by qRT-PCR (A), and the cell lysates were analyzed by western blotting (B). (C) Culture supernatants were collected at 8 hpi and 20 hpi, respectively. PEDV titers in the culture supernatants were measured as TCID50. (D and E) Vero cells were transfected with CMPK2 siRNA (#1, #2, and #3) and negative control (siCMPK2#NC) for 12 h and then infected with PEDV (MOI = 1). Total RNA of the cells harvested at 20 hpi were extracted and analyzed by qRT-PCR, and the cell lysates were analyzed by western blotting. (F) Culture supernatants were collected at 8 hpi and 20 hpi, respectively. PEDV titers in the culture supernatants were measured as TCID50. Data are means ± SD of triplicate samples; statistical analysis was conducted using one-way ANOVA followed by Dunnett’s multiple comparison test or two-way ANOVA followed by Tukey’s multiple comparison test; only the p-value for the most relevant comparisons are shown for simplicity. The intensities of bands were quantified by ImageJ. **p < 0.01, ***p < 0.001, ****p < 0.0001. Data underlying this figure can be found in S1 Data and S1 Raw Images. CMPK2, cytidine/uridine monophosphate kinase 2; hpi, hours post-infection; MOI, multiplicity of infection; PEDV, porcine epidemic diarrhea virus; qRT-PCR, quantitative real-time PCR; siRNA, small interfering RNA. (TIF) [file pbio.3002039.s009.tif]

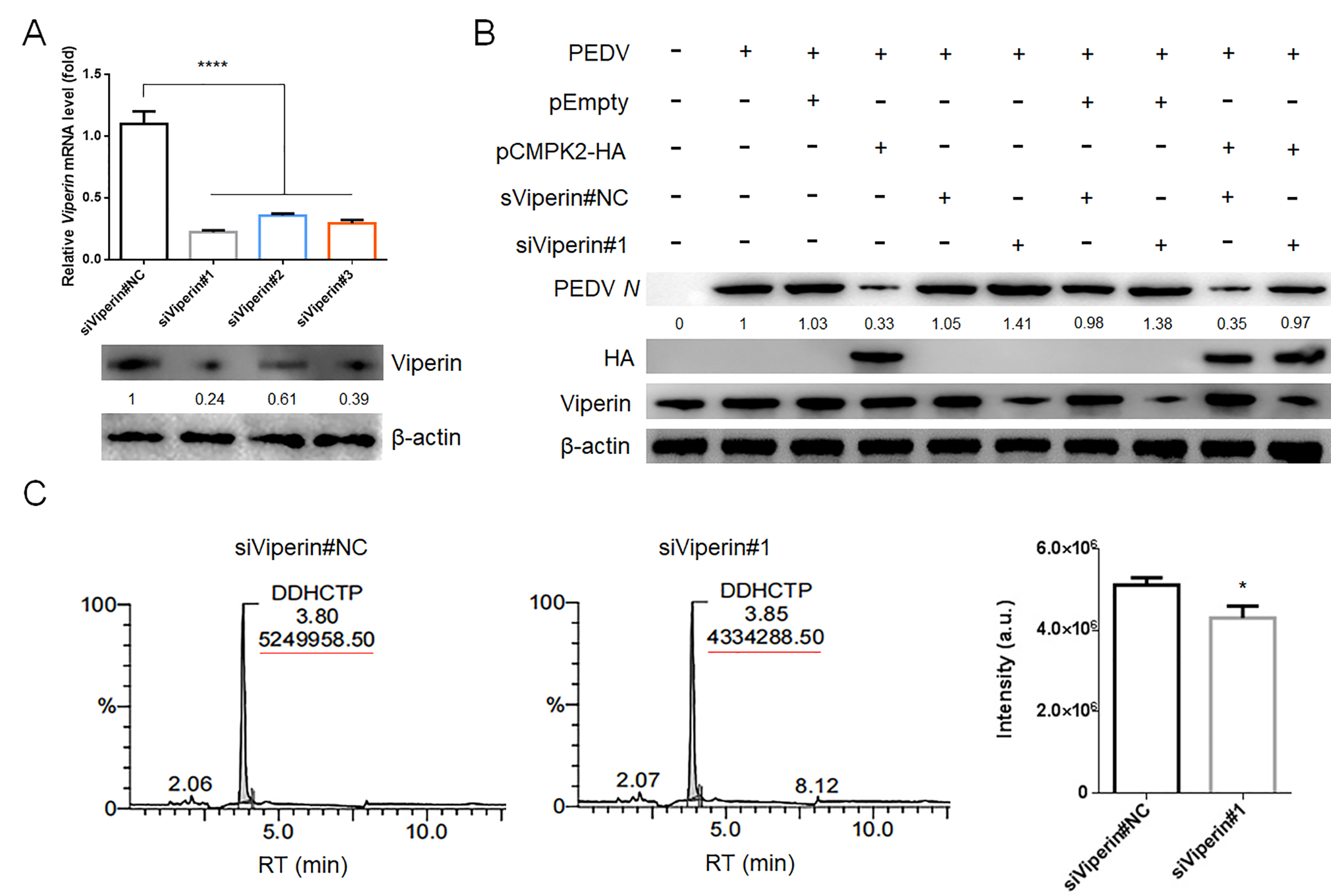

Supplement: S11 Fig — (A) The knockdown efficiency of Viperin. IPEC-J2 cells were transfected with Viperin siRNA (#1, #2, and #3) or negative control (siViperin#NC) for 12 h. Viperin expression level was analyzed by qRT-PCR and western blotting. β-actin was used as the sample loading control. (B) IPEC-J2 cells with or without CMPK2 expression were transfected with siViperin#1 as well as the siViperin#NC and then infected with PEDV at MOI of 1. The cells were harvested at 20 hpi, and the lysates were analyzed by western blotting. (C) IPEC-J2 cells were transfected with siViperin#1 and siViperin#NC for 12 h, and ddhCTP production was detected by LC–MS. Data are means ± SD of triplicate samples; statistical analysis was conducted using one-way ANOVA followed by Dunnett’s multiple comparison test; only the p-value for the most relevant comparisons are shown for simplicity. The intensities of bands were quantified by ImageJ. *p < 0.05. Data underlying this figure can be found in S1 Data and S1 Raw Images. CMPK2, cytidine/uridine monophosphate kinase 2; ddhCTP, 3′-deoxy-3′,4′-didehydro-cytidine triphosphate; hpi, hours post-infection; LC–MS, liquid chromatography followed by mass spectrometry; MOI, multiplicity of infection; PEDV, porcine epidemic diarrhea virus; qRT-PCR, quantitative real-time PCR; siRNA, small interfering RNA. (TIF) [file pbio.3002039.s011.tif]

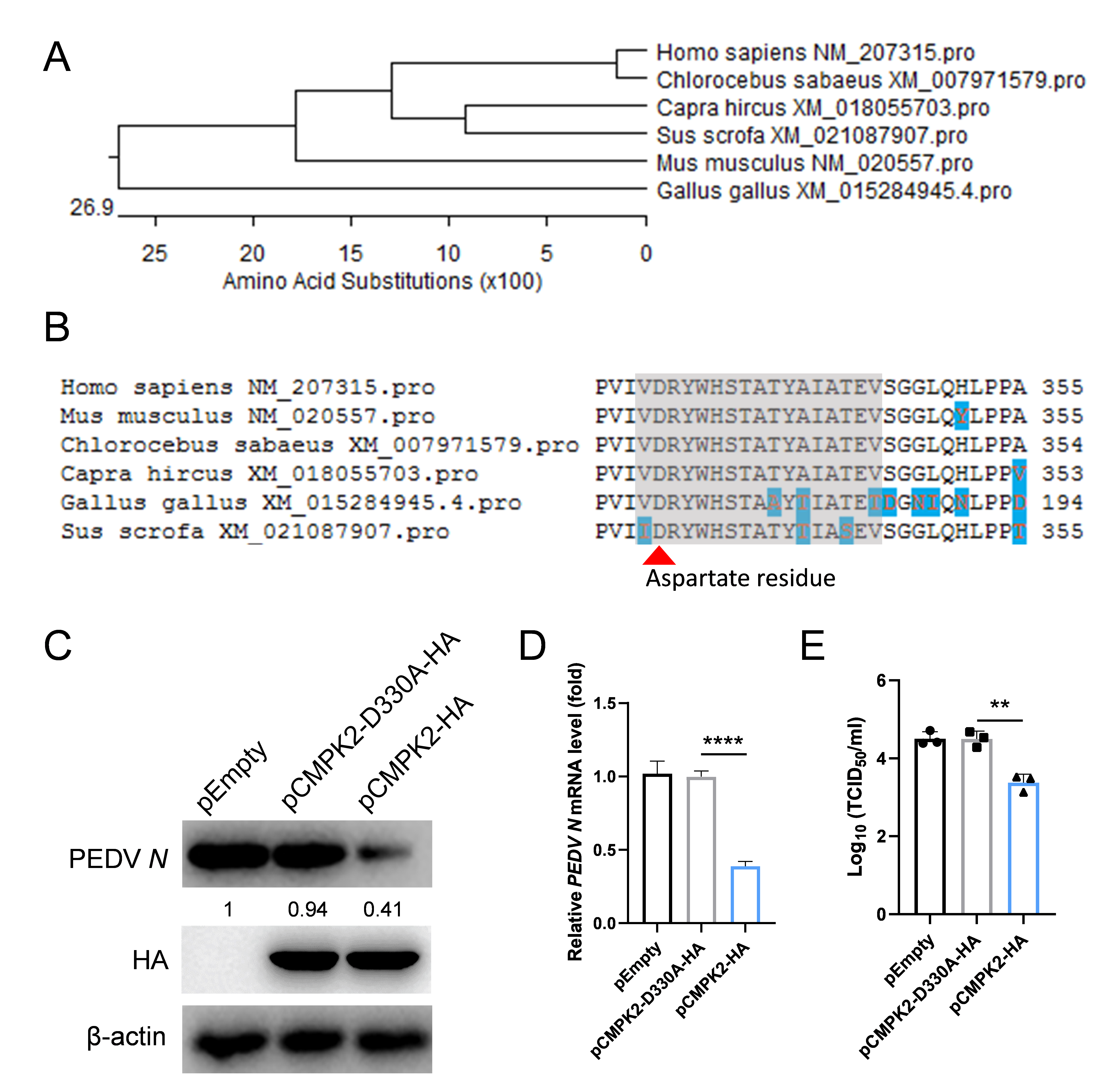

Supplement: S12 Fig — (A) Phylogenetic analysis of full-length CMPK2 was shown with cladogram. (B) The comparison of CMPK2 catalytic site sequences between different species. Gray indicates the comparison of identified poultry CMPK2 catalytic sites across multiple species. Blue indicates different amino acids of CMPK2 among the above species. The highly conserved aspartate (D) residue are labeled in red triangle. (C-D) The actual catalytic mutant of CMPK2 (CMPK2-D330A-HA) was constructed, and IPEC-J2 cells were transfected with CMPK2-D330A-HA, CMPK2-HA, and empty vector respectively followed by PEDV infection for 20 hpi. The cell lysates were analyzed by western blotting (C), and total RNA of the cells were extracted and analyzed by qRT-PCR (D). (E) Culture supernatants were collected at 20 hpi, and PEDV titers were measured as TCID50. Data are means ± SD of triplicate samples; statistical analysis was conducted using one-way ANOVA followed by Dunnett’s multiple comparison; only the p-value for the most relevant comparisons are shown for simplicity. The intensities of bands were quantified by ImageJ. **p < 0.01. ****p < 0.0001. Data underlying this figure can be found in S1 Data and S1 Raw Images. CMPK2, cytidine/uridine monophosphate kinase 2; hpi, hours post-infection; PEDV, porcine epidemic diarrhea virus; qRT-PCR, quantitative real-time PCR. (TIF) [file pbio.3002039.s012.tif]

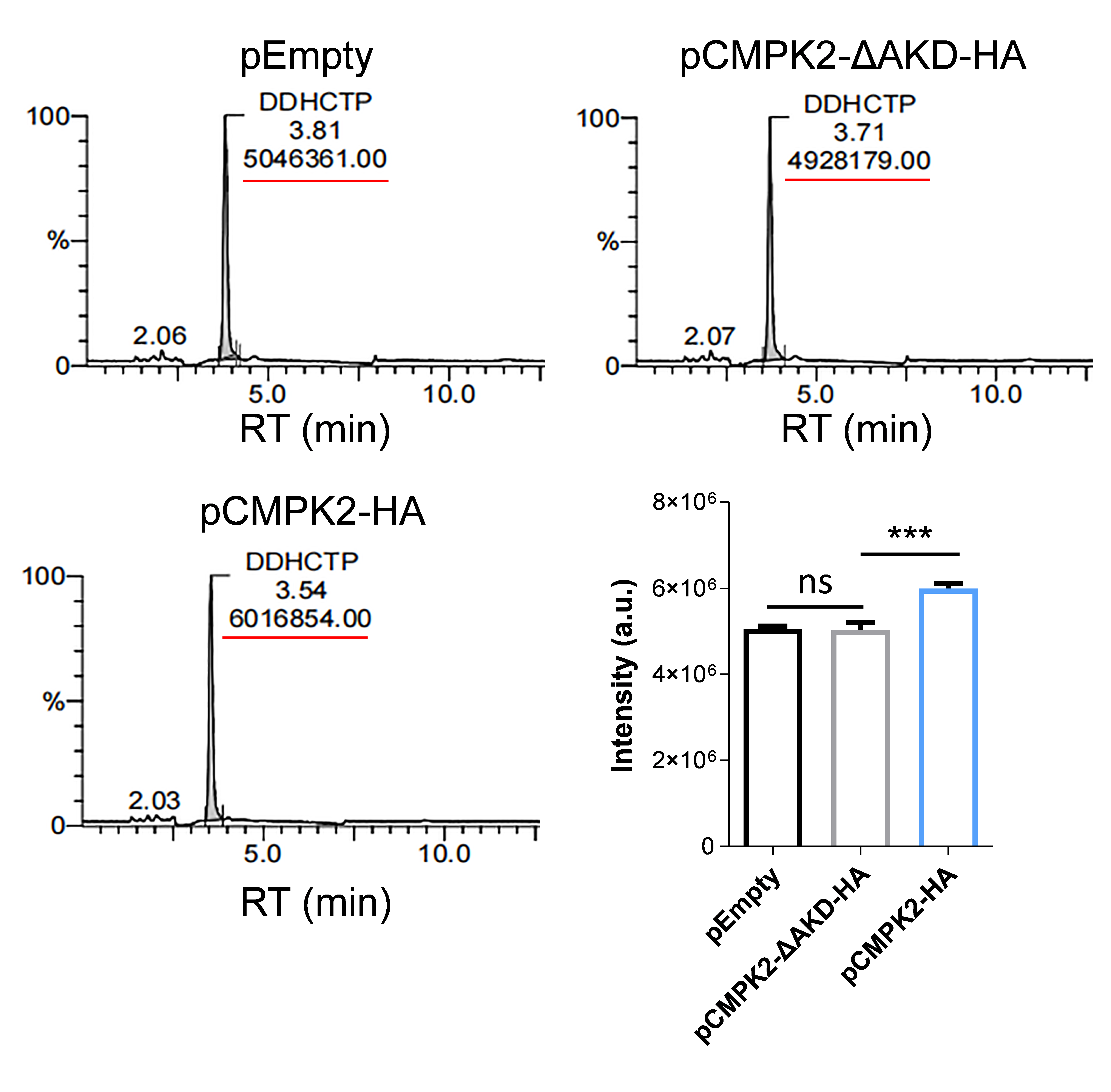

Supplement: S13 Fig — IPEC-J2 cells were transfected with AKD deletion mutant (pCMPK2-ΔAKD-HA) and pCMPK2-HA, respectively, and then ddhCTP production was detected by LC–MS. Data are means ± SD of triplicate samples; statistical analysis was conducted using one-way ANOVA followed by Dunnett’s multiple comparison; only the p-value for the most relevant comparisons is shown for simplicity. ns, no significance. Data underlying this figure can be found in S1 Data and S1 Raw Images. AKD, antiviral key domain; CMPK2, cytidine/uridine monophosphate kinase 2; ddhCTP, 3′-deoxy-3′,4′-didehydro-cytidine triphosphate; LC–MS, liquid chromatography followed by mass spectrometry. (TIF) [file pbio.3002039.s013.tif]

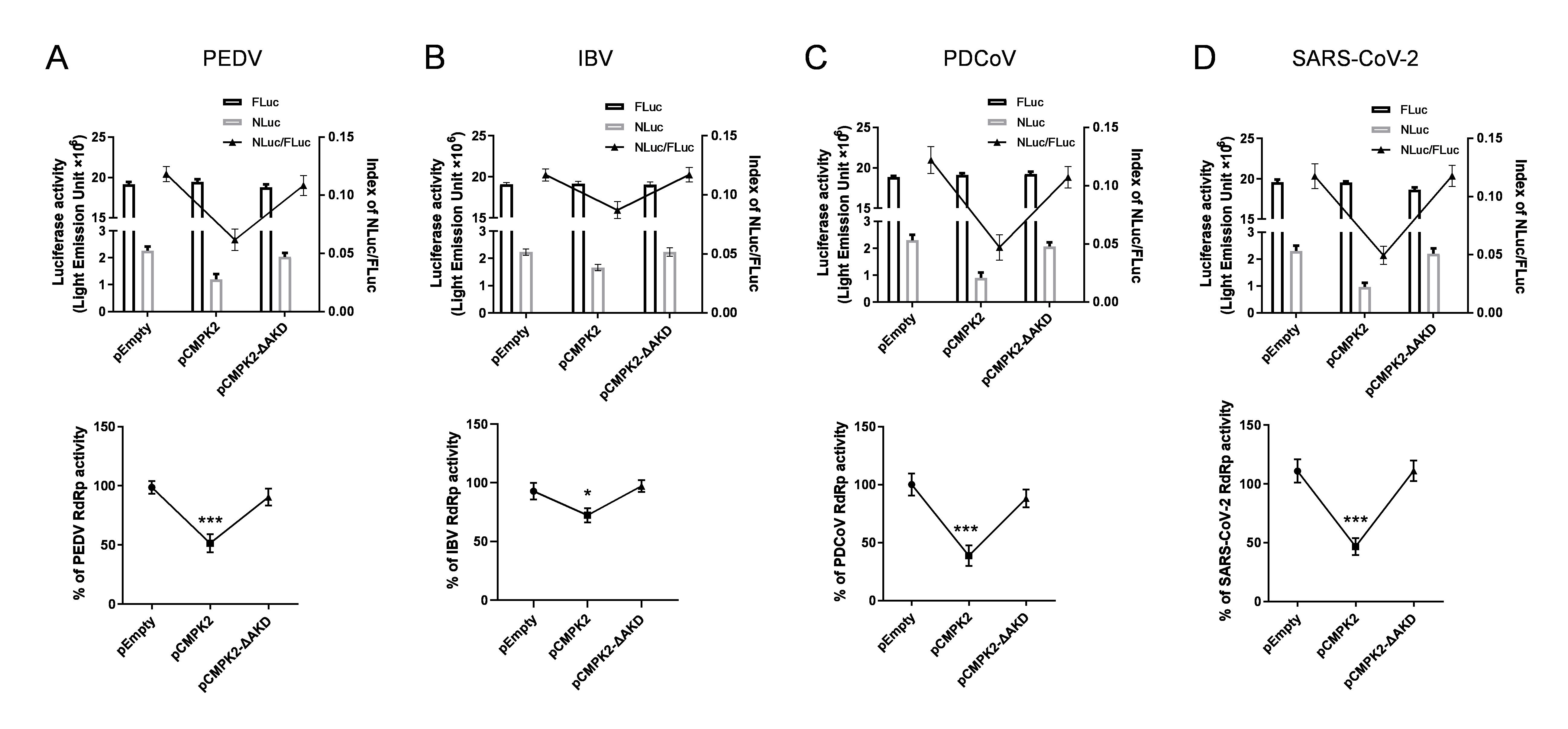

Supplement: S14 Fig — HEK293T cells were transfected with pCMPK2-ΔAKD, pCMPK2, and empty vector, respectively, and then RdRp activity of PEDV (A), IBV (B), PDCoV (C), and SARS-CoV-2 (D) were detected by the cell-based reporter assay system, respectively. Data are means ± SD of triplicate samples; statistical analysis was conducted using one-way ANOVA followed by Dunnett’s multiple comparison; only the p-value for the most relevant comparisons are shown for simplicity. ***p < 0.001. Data underlying this figure can be found in S1 Data. AKD, antiviral key domain; CMPK2, cytidine/uridine monophosphate kinase 2; CoV, coronavirus; IBV, infectious bronchitis virus; PDCoV, porcine delta-coronavirus; PEDV, porcine epidemic diarrhea virus; RdRp, RNA-dependent RNA polymerase; SARS-CoV-2, Severe Acute Respiratory Syndrome Coronavirus 2. (TIF) [file pbio.3002039.s014.tif]
