# Supplementary material for: CMPK2 is a host restriction factor that inhibits infection of multiple coronaviruses in a cell-intrinsic manner
Source: PLoS Biol. 2023 Mar 17;21(3):e3002039. doi: 10.1371/journal.pbio.3002039 (PMC10058120; doi:10.1371/journal.pbio.3002039)
Supplement: S1 Table — (DOCX) [file pbio.3002039.s015.docx]

**S1 table.** Nucleotide sequence of the porcine CMPK2 promoter.

| Purpose | Sequence (5’-3’) |
| --- | --- |
| Porcine promoter CMPK2 sequence | GTGTGTGTGTGCTGGGTTGTGCTTCAGGACTCTCAAGACCACCCACATGCTTGCTGATTGGCTGCAAGCACTTGTAGGACTAGGAGCAGGTCTTACCACAGGTGTGGTTTACTCCAAGCCAAGGATGCAGGGCAAGAACAACAAAGCAACAAAGAAACGAGGAACATCAGGAGTCTGGAGAGGTGGGCACAGCTTCTAGATCCTTCACCATGTGGGTTGCAGAACTAGATGTGCTTTCTCTCTGGCATCAGGCCCCCCAGACAGGTCTGCCTTATGTGCTGGGCTCCACCTGTAGGCACATCCTCTGTGCCATCTTTGGTCACCTAATCGCAGGACCCCCATCTGTGAATGAGGCACCTGTCCCCATTTAGAGTTATTGGTGTAAAATTCTATGTAATAGTCTCTCTTTTTGTGTCCTTTGTAATCTGTGGTTCTTCCCTCTCTTGACTATAATAGTAACACAATGCTGATGCATAATTTCATTTTCTTTTTATCCACCTAAATGATCGACCTACAAGCATATCTGTTAAACAGATTTTCAAAAACAAGCTCCTTTTCAAAATCTCATTTTTAATTTTCCTATTAACTACGCATTTGTATTAGTTAATTCTCCTTTTCCTTTTTCCTAGAATGACTAGACTATTTCACATTTCCCTGATGGATGCTCTAAGTGTTGAAAATGCTGCTTTTTAAATGAATGCATCCCTGAAATTCATTCATTCAAGCACCTCTTTGAAGTTTCTTTCCACCTCCCCAGCTTCTCGCCTCCACACCCACTGGGTGGGTTAATACAAACAAGTTAATTCAACCTCAATACTCACCTAAGGGGAGTCCATCAGATTCTCTGTCCTAGGCATTTGAAATTGAAGCTCAAAAATATGACAGAGAATTTCTCCTTTATGAATGAAGATAGTGTATCTTCCATATTCAATATGTAGTTAACATTGACTGAGCCCAGATCCTGCTGCATCATTAATTTCACAGCATCCTGGTAGGCAGGAGGGGGGTGAAAATGAGGAGGAAAACTTTAAGAGTGGGTATGAAGCGGGGCAGTGGGGGGGGGGTCCTGGAAAAAAGCTCAAGAAAGGCTCCTGGGAGGATGCCGTCCTCTACTAGAGAACAAAAAGCAGTTTGCCCTTTTTCTCTTTTCCTGTGGGTCACACTTGCTCCCTAAATGTCCCAAAGTCCAATTGTCCATAGAGAATAAAAATGATGCTGTTCTCAAAGAGTGAAAACATCTTAACCTCAGAATTATCCCTTGACTATAAGCTGATACTTCAACCACACCACGTTTATCAAAAAGGGTTGGTCTTTCAGGGTTAAACTGGATCCCATGCTAAAGGCACATTGATCAAATCTAATCCTTTGTTTTCAGTTTCAGTTTCCCTAATGTTTTCCGTCTTTGTCTCAAGTCCATTTTTTTTTTTTGTTTTTTGGTCTGCTTGACAATTACTTTCAAAGATATGTTAGGTATAAAAACAAAATAAAATATCCGAATAATCTTGCAAATGTGGCTTTACTATACAAAGCCAGCAATACCTCTGCTGCTGGCTGCCAAAGGCTCTGTCAGGCCAGCATGCTCAAGTCTCCTTGGACCTGTGCGCCCGACCTCAATGTGGCATTTGAAGGTAATTTAGAGGTGCCAATCTGGTGACACTTCTCAGGTTGAGAGTCAGCAGAAGGAGGTGGGTAACGCCCAAAGAGGGACGAGAATGTGCAAAGCAAAATGAATGAAAGCGCTTCCTGTTTAGGCAACAGAGCTGTTCCCAGGACTGGAGTCAGGGCCTAGGCTGGGAGAGGCGGTCGAGCTTAGGGCTGAGGTCGGGGAGCGGCTGTGGGGTCAGCGGGCCCGCTAGGCGAGCCCAGGTGAGGCGTCATCGGTGCCAGAGGGCAATGAGGAGCTGGTGAGGGTGGTGGGGAAGGGGAGAGACCTCGATCTAAAAAGCACACACACATGCCCCGTGCACTGGGCATAAGCAACACAGGAGGAATTATTTAAA |
