# Supplementary material for: CMPK2 is a host restriction factor that inhibits infection of multiple coronaviruses in a cell-intrinsic manner
Source: PLoS Biol. 2023 Mar 17;21(3):e3002039. doi: 10.1371/journal.pbio.3002039 (PMC10058120; doi:10.1371/journal.pbio.3002039)
Supplement: S2 Table — (DOCX) [file pbio.3002039.s016.docx]

**S2 table.** Primer, siRNA and sgRNA sequences used in this study.

| Purpose | Names | Sequence (5’-3’) |
| --- | --- | --- |
| Real-time PCR Primers | *GAPDH* forward | ACATGGCCTCCAAGGAGTAAGA |
|  | *GAPDH* reverse | GATCGAGTTGGGGCTGTGACT |
|  | PEDV *N* forward | GTCTGAAAAGCCAATCATTC |
|  | PEDV *N* reverse | TTGCCTCTGTTGTTACTC |
|  | IBV *N* forward | CAAGCTAGGTTTAAGCCAGGT |
|  | IBV *N* reverse | TCTGAAAACCGTAGCGGATAT |
|  | PDCoV *M* forward | ATCGACCACATGGCTCCAA |
|  | PDCoV *M* reverse | CAGCTCTTGCCCATGTAGCTT |
|  | *CMPK2* forward | CATTCCCGAAGCCCAAGC |
|  | *CMPK2* reverse | TGGCGACTTTAGGAGAACAGC |
|  | **CMPK2* forward | CCTGCTTAAACCTGACCTCATC |
|  | **CMPK2* reverse | CCATCCGCTGGTAGGACATT |
|  | *CMPK2* promoter forward | AATGTCCCAAAGTCCAAT |
|  | *CMPK2* promoter reverse | AACCCTGAAAGACCAACC |
|  | *CXCL10* forward | CACCATACATCAAGCCCTAA |
|  | *CXCL10* reverse | CAGGACTTGGCACATTCA |
|  | *IFIT1* forward | TCTTGGAGGAGATTGAGT |
|  | *IFIT1* reverse | ATACAGCCAGGCATAGTT |
|  | *MX1* forward | GAGTGTCGGCTGTTTACC |
|  | *MX1* reverse | TGGCGTCATAACCATTTT |
|  | *IRF1* forward | CAGCCGAGATGCTAAGTG |
|  | *IRF1* reverse | TGACAGTGCTGGAGTAAGG |
|  | *NF-κB1* forward | GGATTTCGTTTCCGTTATGT |
|  | *NF-κB1* reverse | CTGAGGGTAGGACTTCTTG |
|  | *STAT2* froward | CTGAGGGTAGGACTTCTTG |
|  | *STAT2* reverse | CAGTGTCAGATAGCCGAAG |
|  | *STAT5b* forward | TCAAGCCTCATTGGAACG |
|  | *STAT5b* reverse | TGAACCTCAGCAGGAAAGTC |
|  | *Viperin* forward | CTTGGTGCCCGAGTCTAAC |
|  | *Viperin* reverse | CTTCAGGTCCGCCTTGCT |
| siRNA target sequences | si-*IRF1* | CCAGATCCCATGGAAGCAT |
|  | si-*CMPK2* | AGAGGATGAAGAGGATCGA |
|  | si-*Viperin* | AGAGATTCCTACCTTATTC |
| sgRNA target sequences | sg-*CMPK2* | TGCTGCTCGCGCGCCTCTCG |
|  | sg-*Viperin* | GTAATTGACGCTAGTGGGGG |

* Chlorocebus sabaeus.
