# Supplementary material for: CMPK2 is a host restriction factor that inhibits infection of multiple coronaviruses in a cell-intrinsic manner
Source: PLoS Biol. 2023 Mar 17;21(3):e3002039. doi: 10.1371/journal.pbio.3002039 (PMC10058120; doi:10.1371/journal.pbio.3002039)

CMPK2 is a host restriction factor that inhibits infection of multiple coronaviruses in a cell-intrinsic manner

Mingjun Zhu, Jiahuang Lv, Wei Wang, Rongli Guo, Chunyan Zhong, Avan Antia, Qiru Zeng, Jizong Li, Qingtao Liu, Jinzhu Zhou, Xuejiao Zhu, Baochao Fan, Siyuan Ding, Bin Li

Supporting Information Raw Images: Uncropped and minimally adjusted images for all relevant figures in this article. Areas of interest used in figures are outlined with a red box. Experimental approach, antibody (where relevant), detection strategy and equipment are indicated for each image. Sizes are shown corresponding to molecular weight markers for proteins. Lanes not used in the figure are marked with “X”.

Fig. 1C (CMPK2)

CMPK2, western blot, anti-CMPK2 antibody, chemiluminescence, Tanon 5200

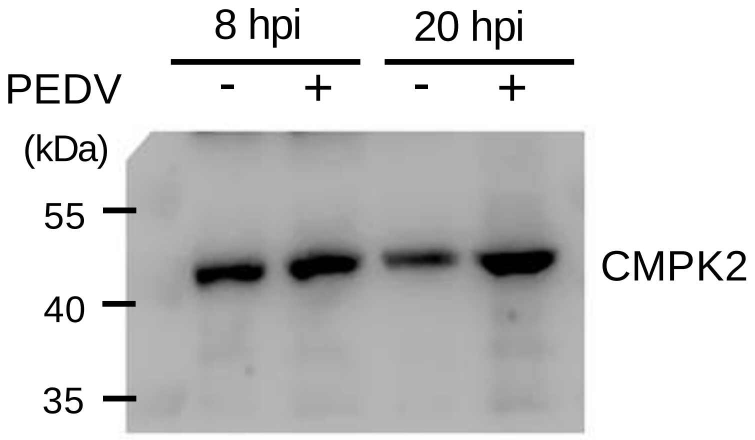

Fig. 1C (PEDV *N*)

PEDV *N*, western blot, anti-N protein antibody, chemiluminescence, Tanon 5200

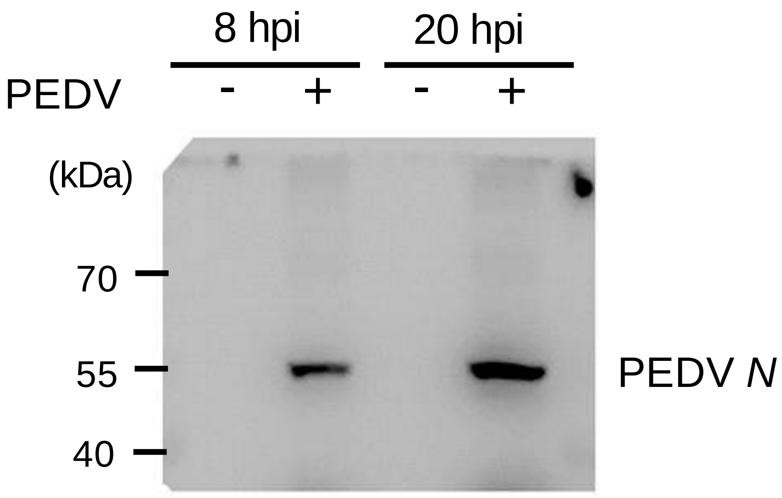

Fig. 1C (β-actin)

β-actin, western blot, anti-β-actin antibody, chemiluminescence, Tanon 5200

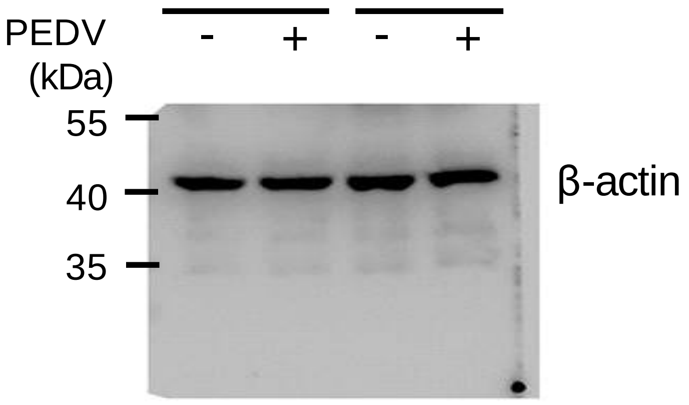

Fig. 1D (CMPK2)

CMPK2, western blot, anti-CMPK2 antibody, chemiluminescence, Tanon 5200

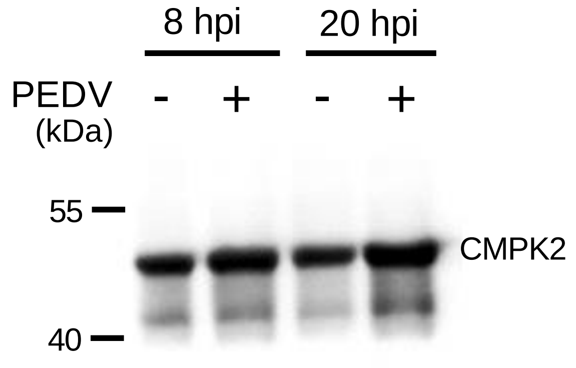

Fig. 1D (PEDV *N*)

PEDV *N*, western blot, anti-N protein antibody, chemiluminescence, Tanon 5200

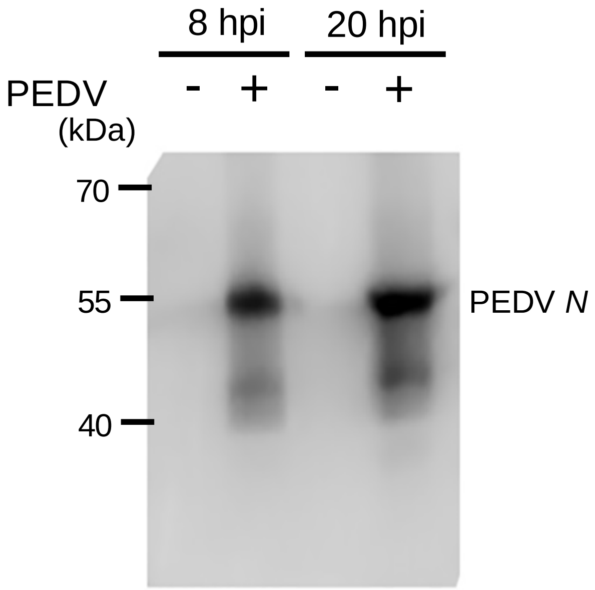

Fig. 1D (β-actin)

β-actin, western blot, anti-β-actin antibody, chemiluminescence, Tanon 5200

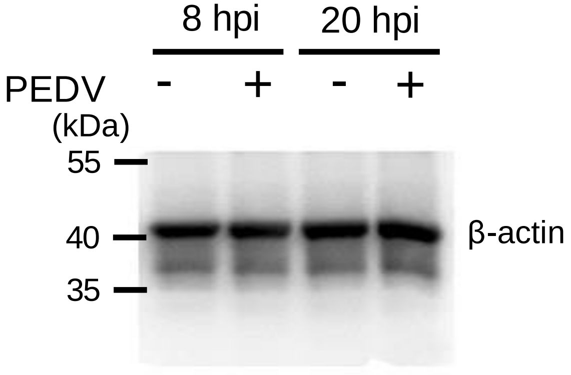

S3 Fig (CMPK2)

CMPK2, western blot, anti-CMPK2 antibody, chemiluminescence, Tanon 5200

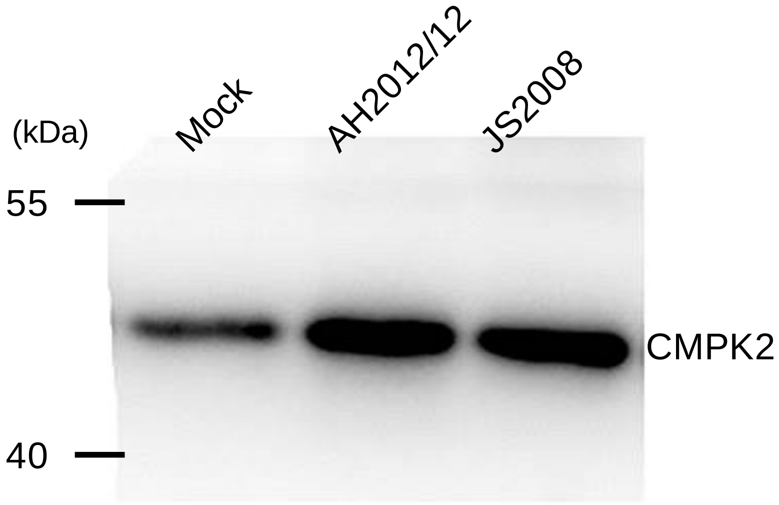

S3 Fig (PEDV *N*)

PEDV *N*, western blot, anti-N protein antibody, chemiluminescence, Tanon 5200

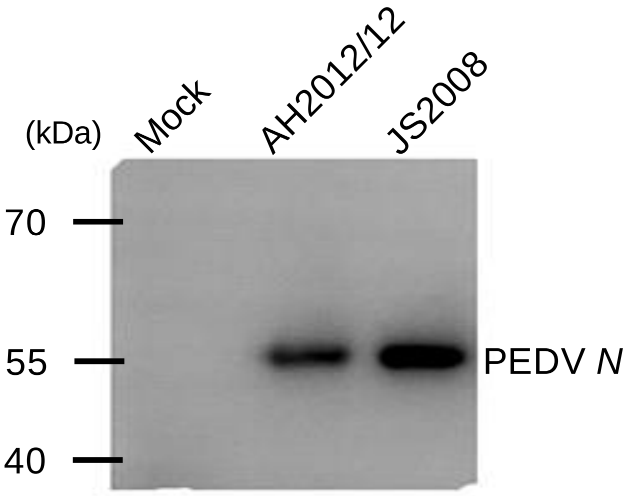

S3 Fig (β-actin)

β-actin, western blot, anti-β-actin antibody, chemiluminescence, Tanon 5200

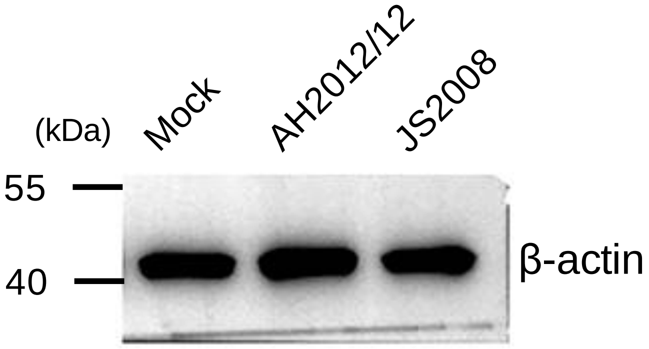

S4 Fig (CMPK2)

CMPK2, western blot, anti-CMPK2 antibody, chemiluminescence, Tanon 5200

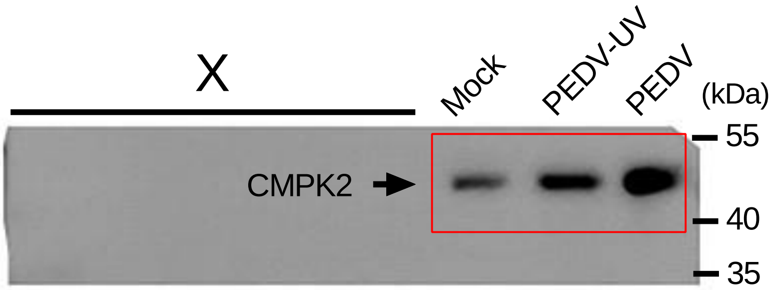

S4 Fig (PEDV *N*)

PEDV *N*, western blot, anti-N protein antibody, chemiluminescence, Tanon 5200

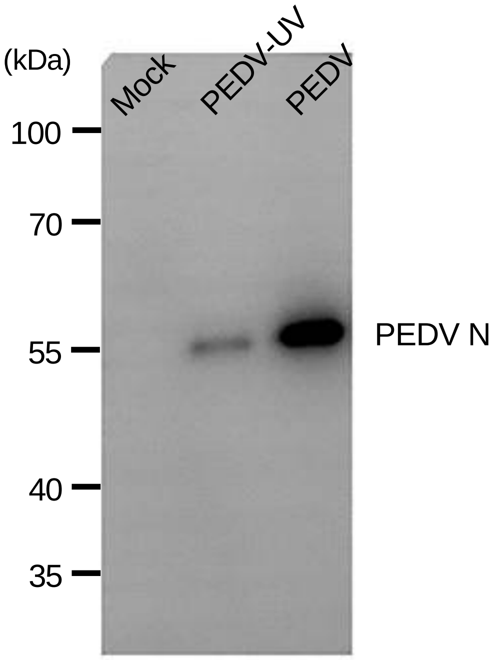

S4 Fig (β-actin)

β-actin, western blot, anti-β-actin antibody, chemiluminescence, Tanon 5200

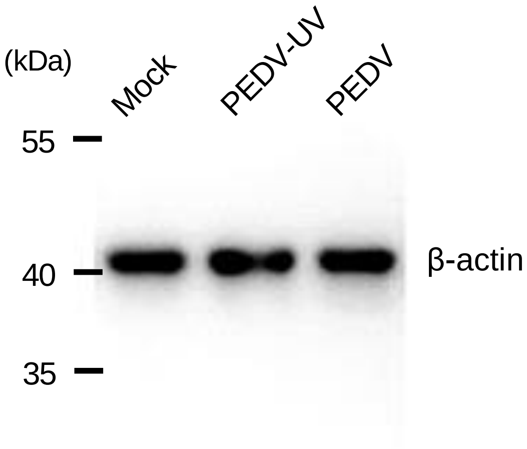

Fig. 2A (CMPK2)

CMPK2, western blot, anti-CMPK2 antibody, chemiluminescence, Tanon 5200

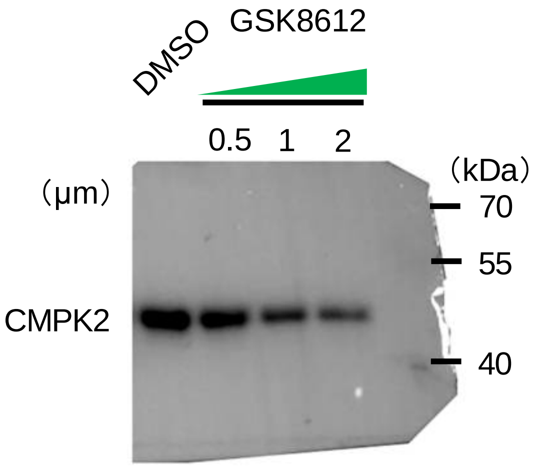

Fig. 2A (pTBK1)

pTBK1, western blot, anti-pTBK1 antibody, chemiluminescence, Tanon 5200

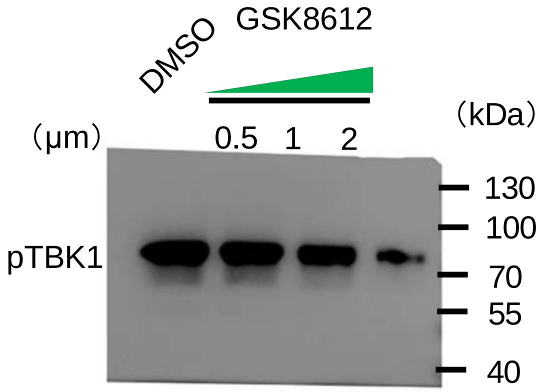

Fig. 2A (TBK1)

TBK1, western blot, anti-TBK1 antibody, chemiluminescence, Tanon 5200

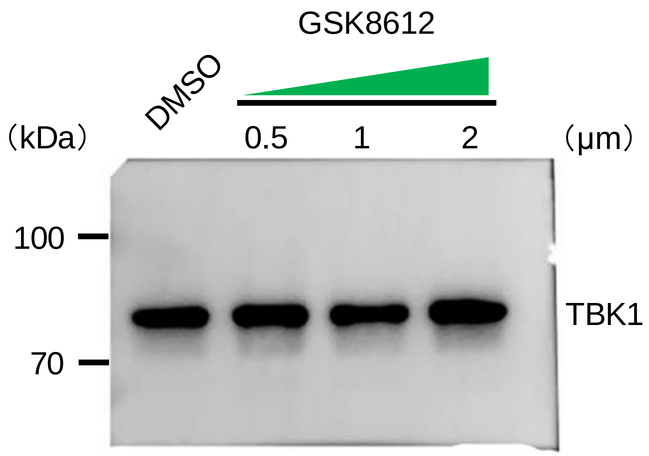

Fig. 2A (β-actin)

β-actin, western blot, anti-β-actin antibody, chemiluminescence, Tanon 5200

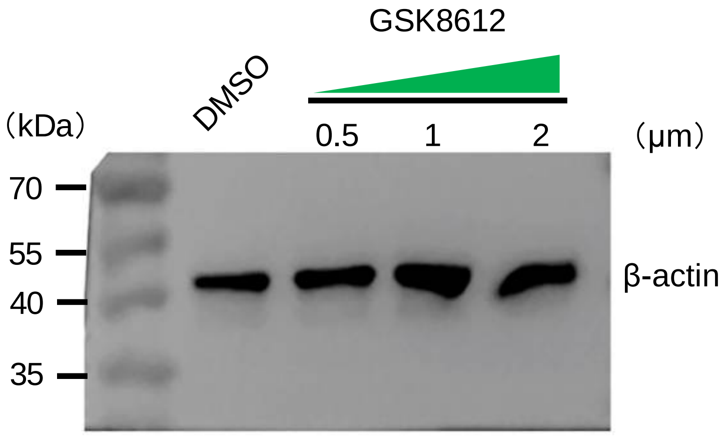

Fig. 2C (CMPK2)

CMPK2, western blot, anti-CMPK2 antibody, chemiluminescence, Tanon 5200

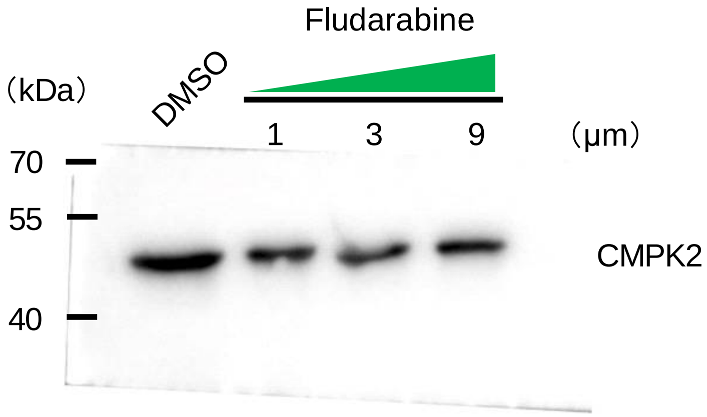

Fig. 2C (pSTAT1)

pSTAT1, western blot, anti-pSTAT1 antibody, chemiluminescence, Tanon 5200

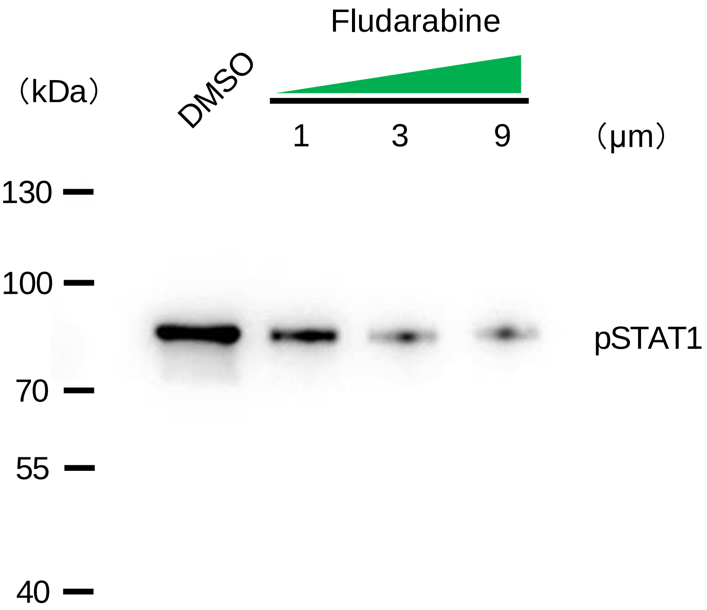

Fig. 2C (STAT1)

STAT1, western blot, anti-STAT1 antibody, chemiluminescence, Tanon 5200

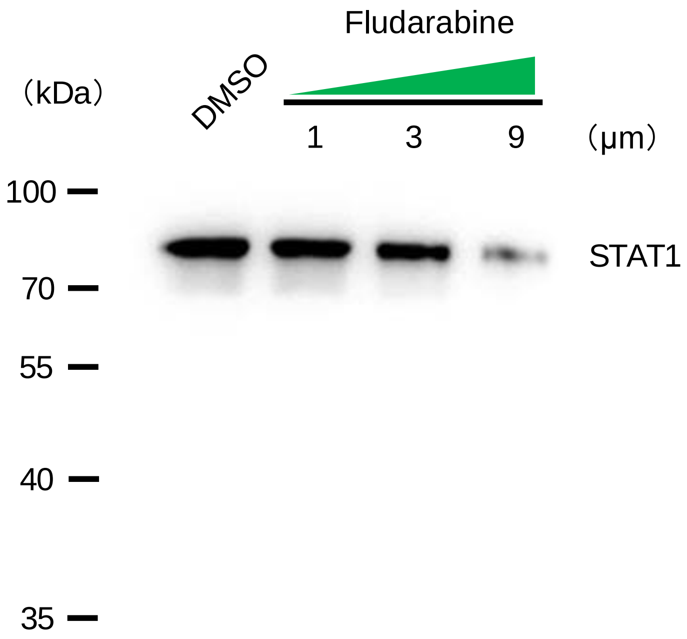

Fig. 2C (β-actin)

β-actin, western blot, anti-β-actin antibody, chemiluminescence, Tanon 5200

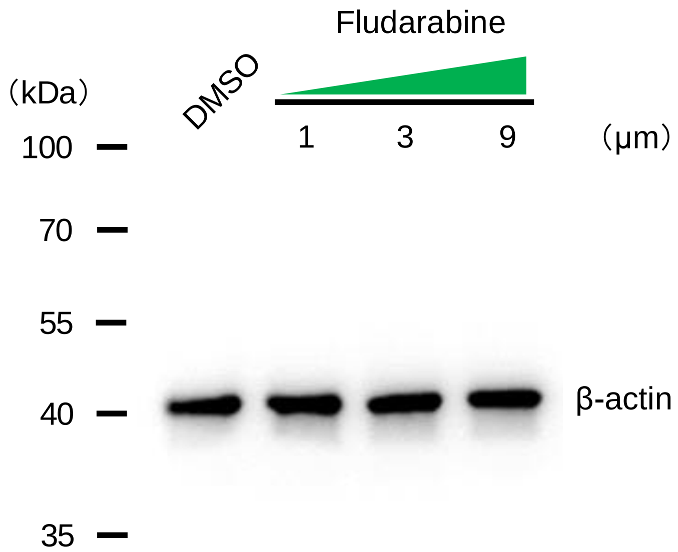

Fig. 2F (CMPK2)

CMPK2, western blot, anti-CMPK2 antibody, chemiluminescence, Tanon 5200

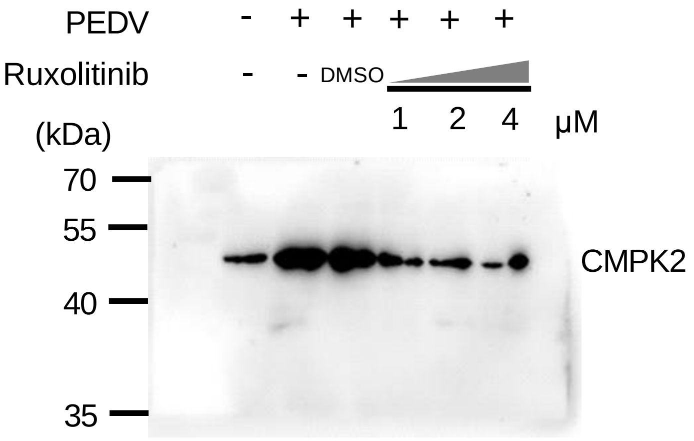

Fig. 2F (JAK2)

JAK2, western blot, anti-JAK2 antibody, chemiluminescence, Tanon 5200

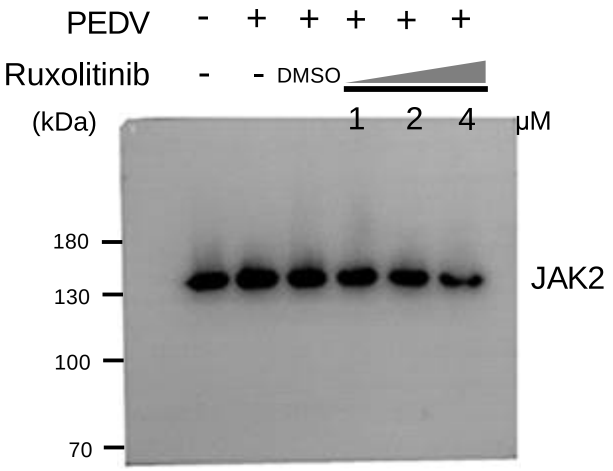

Fig. 2F (STAT1)

STAT1, western blot, anti-STAT1 antibody, chemiluminescence, Tanon 5200

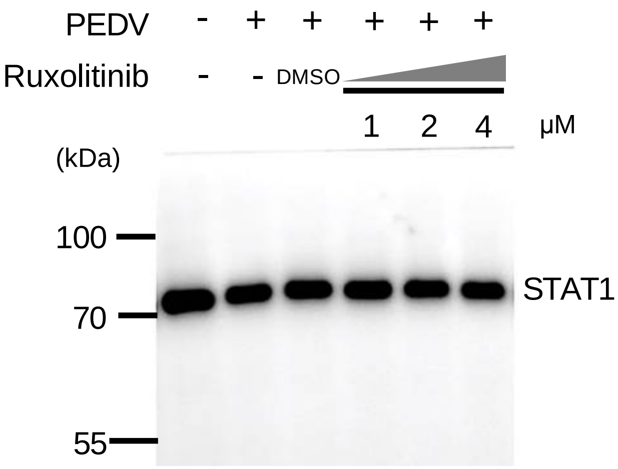

Fig. 2F (JAK1)

JAK1, western blot, anti-JAK1 antibody, chemiluminescence, Tanon 5200

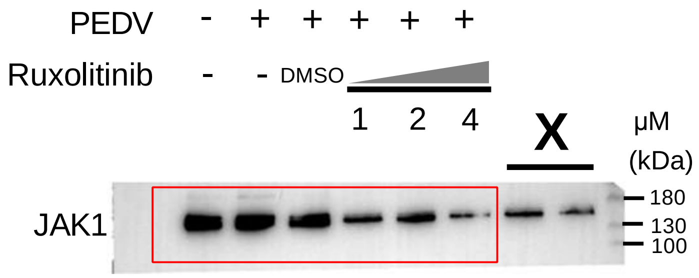

Fig. 2F (pSTAT1)

pSTAT1, western blot, anti-pSTAT1 antibody, chemiluminescence, Tanon 5200

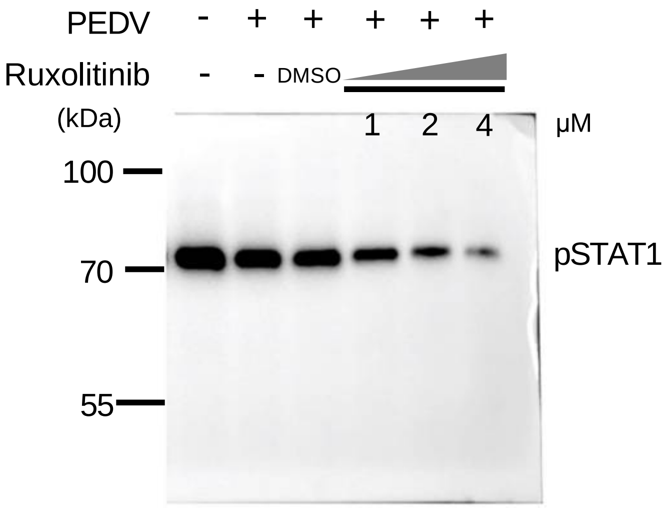

Fig. 2F (β-actin)

β-actin, western blot, anti-β-actin antibody, chemiluminescence, Tanon 5200

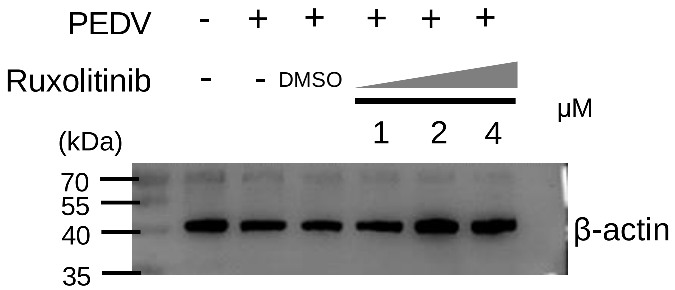

Fig. 3E (IRF1)

IRF1, western blot, anti-IRF1 antibody, chemiluminescence, Tanon 5200

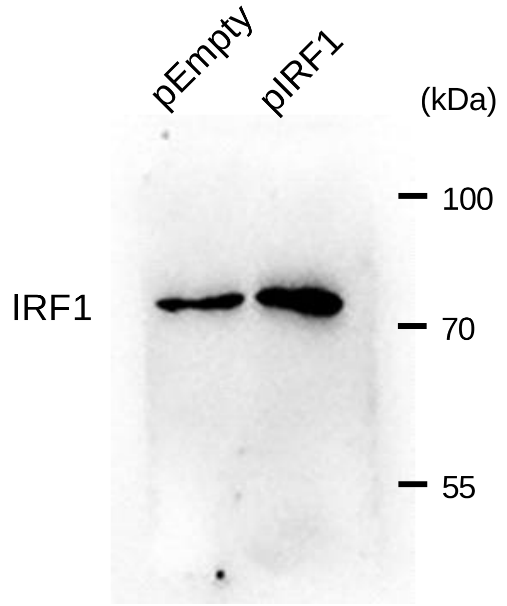

Fig. 3E (CMPK2)

CMPK2, western blot, anti-CMPK2 antibody, chemiluminescence, Tanon 5200

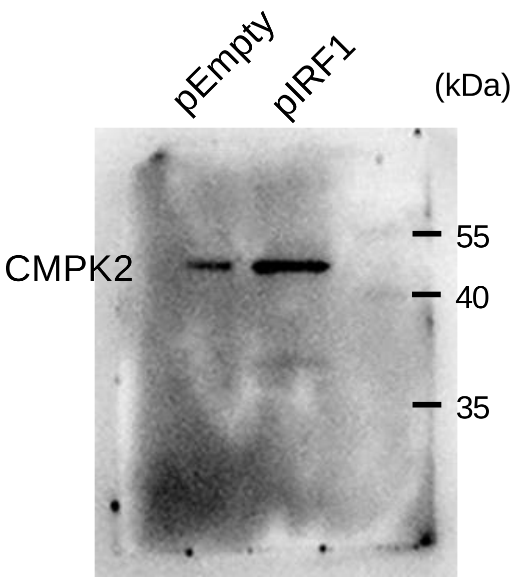

Fig. 3E (β-actin)

β-actin, western blot, anti-β-actin antibody, chemiluminescence, Tanon 5200

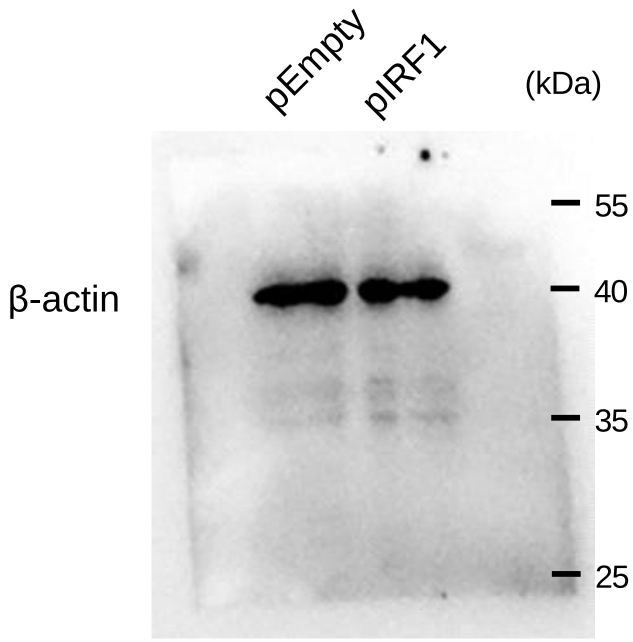

Fig. 3F (PEDV *N*)

PEDV *N*, western blot, anti-N protein antibody, chemiluminescence, Tanon 5200

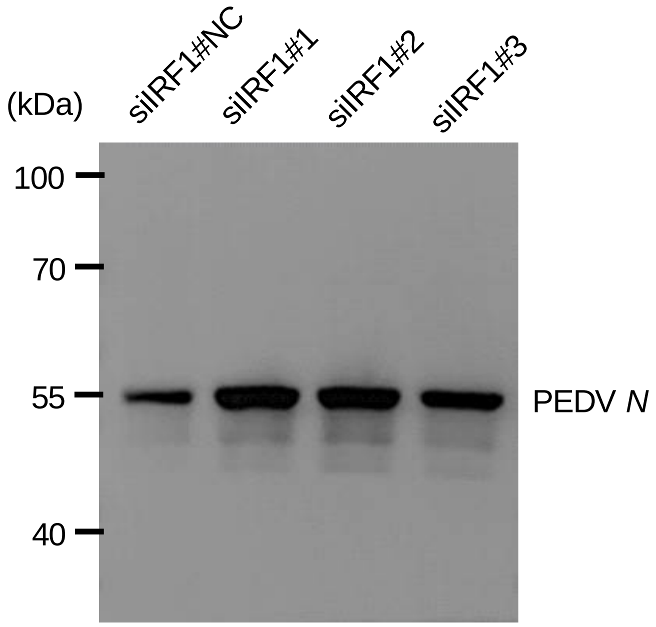

Fig. 3F (CMPK2)

CMPK2, western blot, anti-CMPK2 antibody, chemiluminescence, Tanon 5200

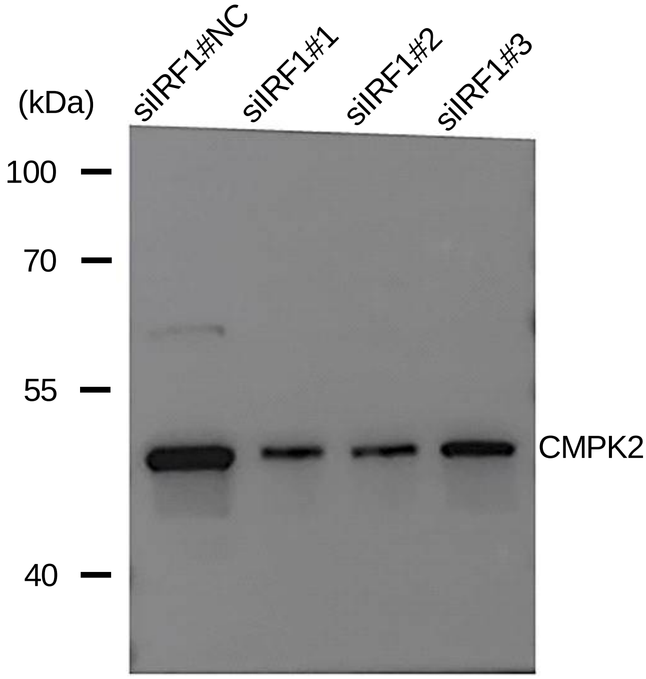

Fig. 3F (IRF1)

IRF1, western blot, anti-IRF1 antibody, chemiluminescence, Tanon 5200

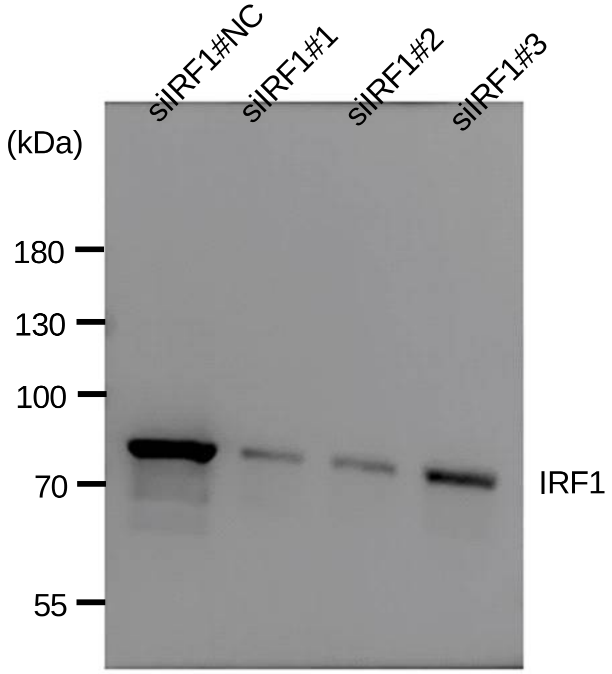

Fig. 3F (β-actin)

β-actin, western blot, anti-β-actin antibody, chemiluminescence, Tanon 5200

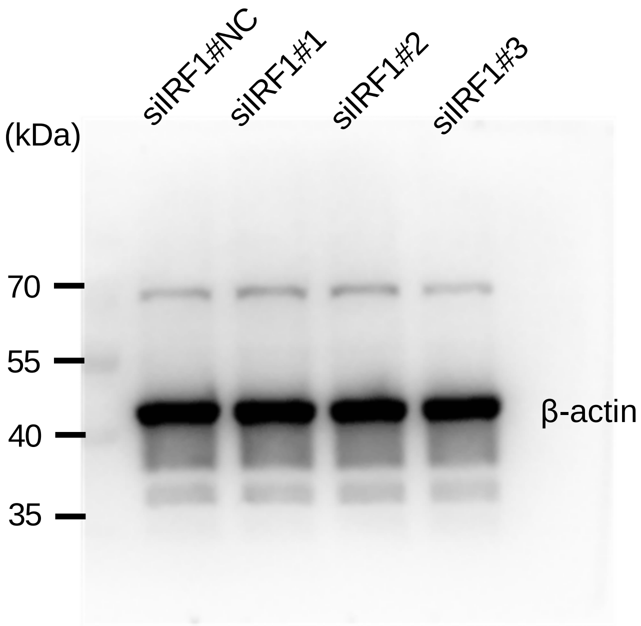

S7A Fig (CMPK2)

CMPK2, western blot, anti-CMPK2 antibody, chemiluminescence, Tanon 5200

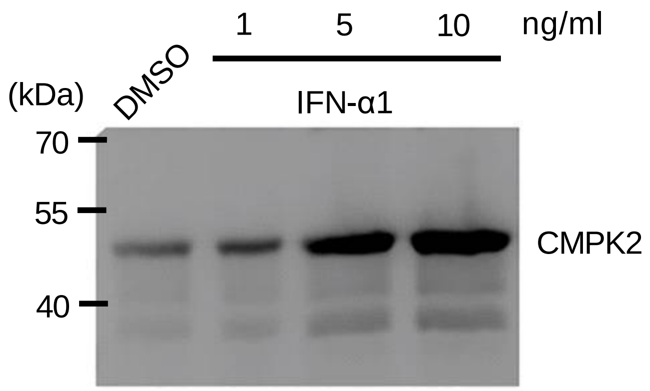

S7A Fig (β-actin)

β-actin, western blot, anti-β-actin antibody, chemiluminescence, Tanon 5200

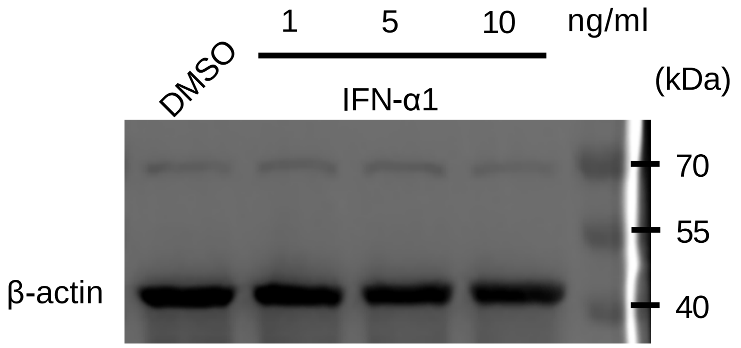

S8 Fig (left)

Whole cell lysates (WCL), western blot, anti-IRF1, CMPK2 and β-actin antibody, chemiluminescence, Tanon 5200

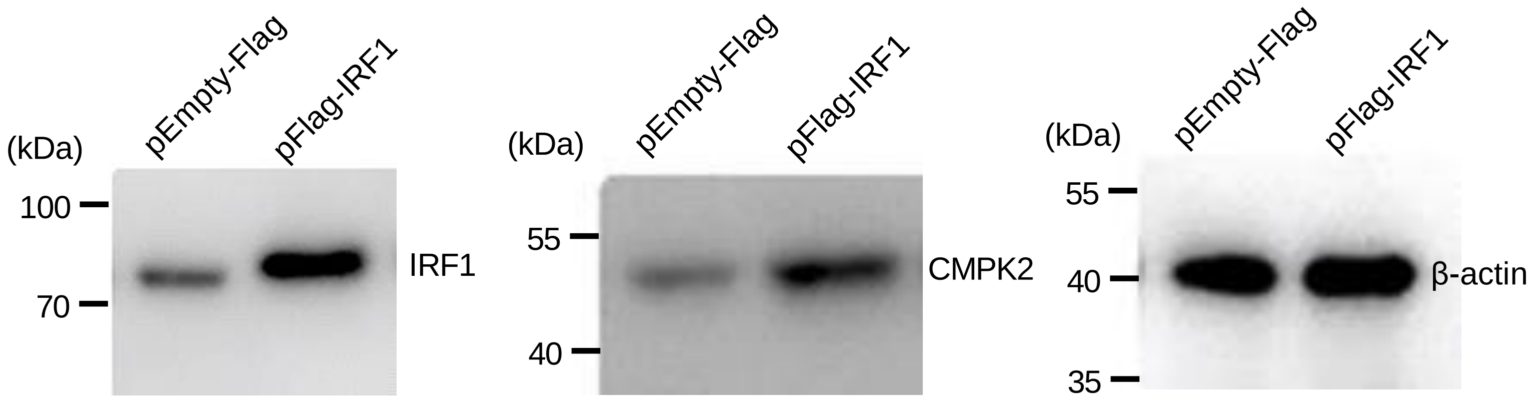

IP: IgG/Flag, western blot, anti-IRF1 antibody, chemiluminescence, Tanon 5200

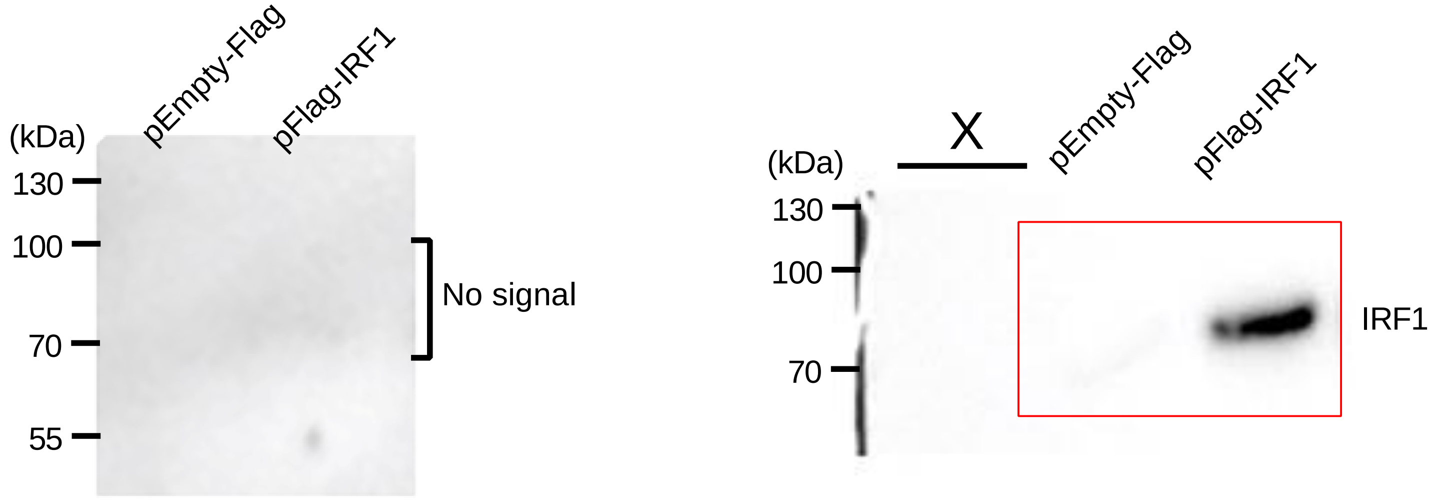

Fig. 4B (PEDV *N*)

PEDV *N*, western blot, anti-N protein antibody, chemiluminescence, Tanon 5200

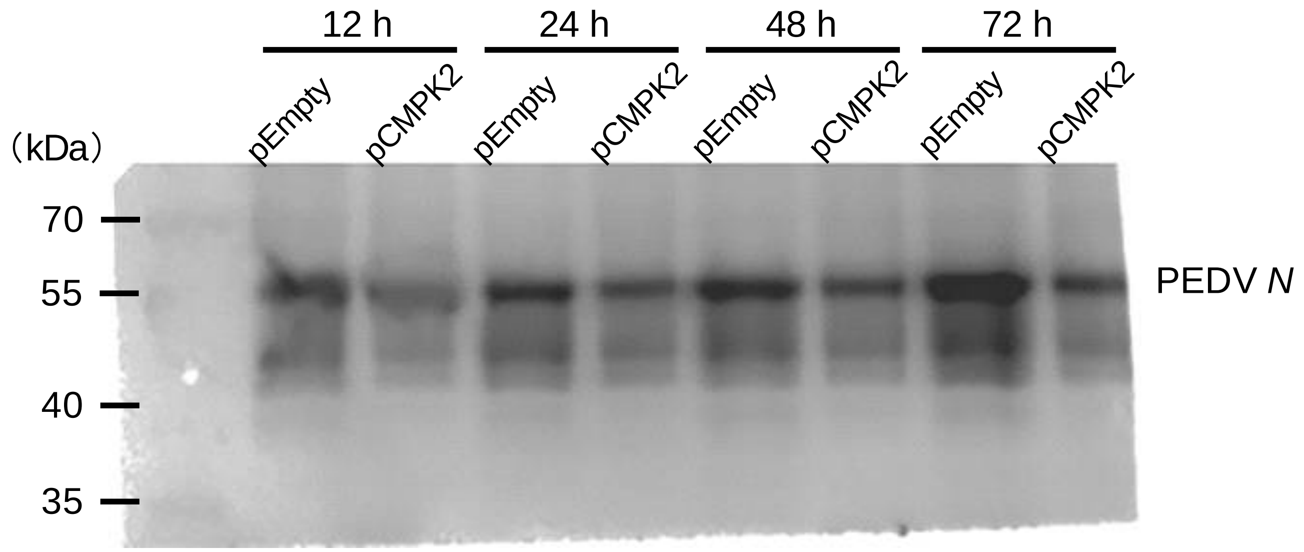

Fig. 4B (CMPK2)

CMPK2, western blot, anti-CMPK2 antibody, chemiluminescence, Tanon 5200

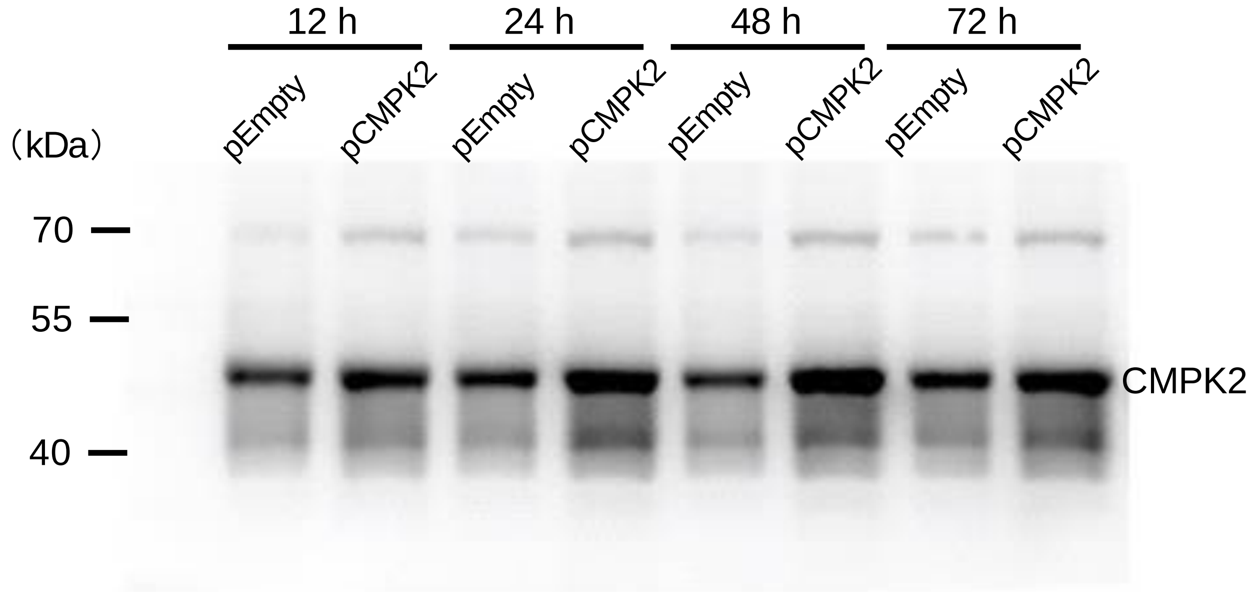

Fig. 4B (β-actin)

β-actin, western blot, anti-β-actin antibody, chemiluminescence, Tanon 5200

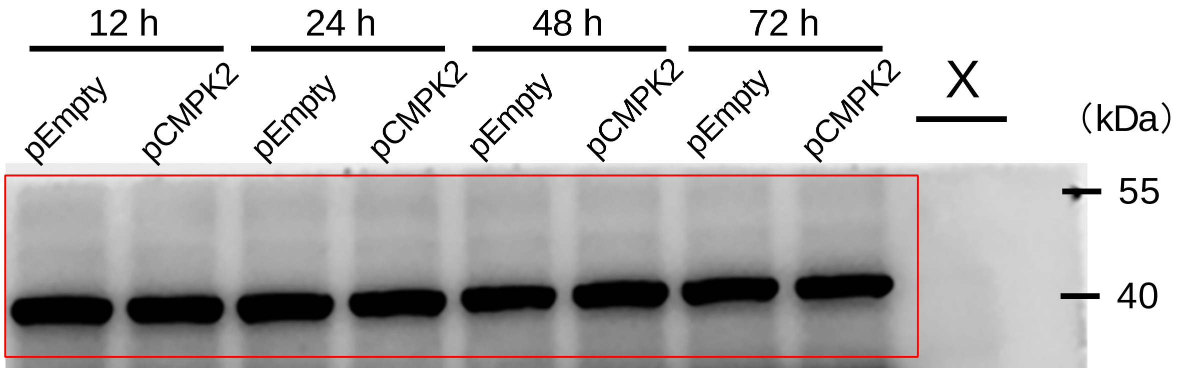

Fig. 4D (PEDV *N*)

PEDV *N*, western blot, anti-N protein antibody, chemiluminescence, Tanon 5200

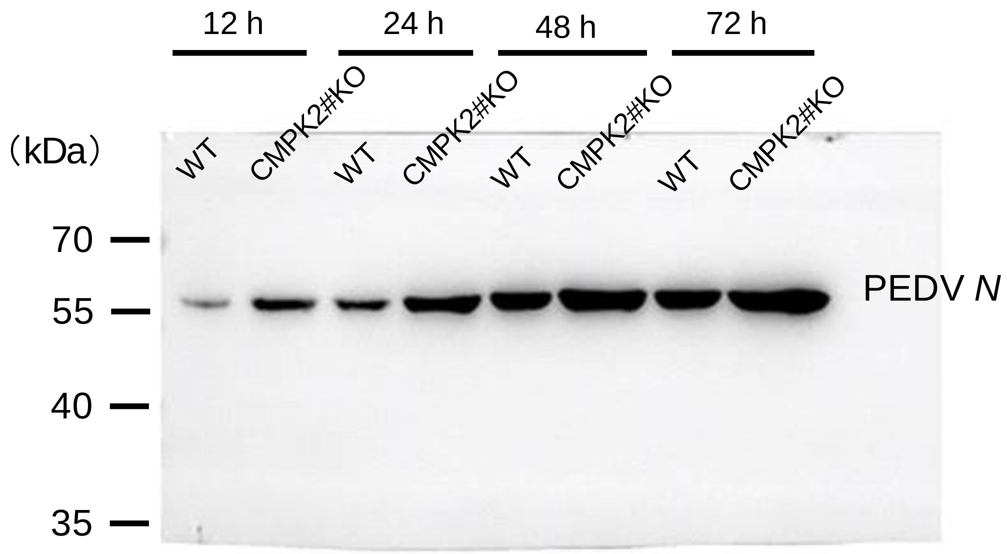

Fig. 4D (CMPK2)

CMPK2, western blot, anti-CMPK2 antibody, chemiluminescence, Tanon 5200

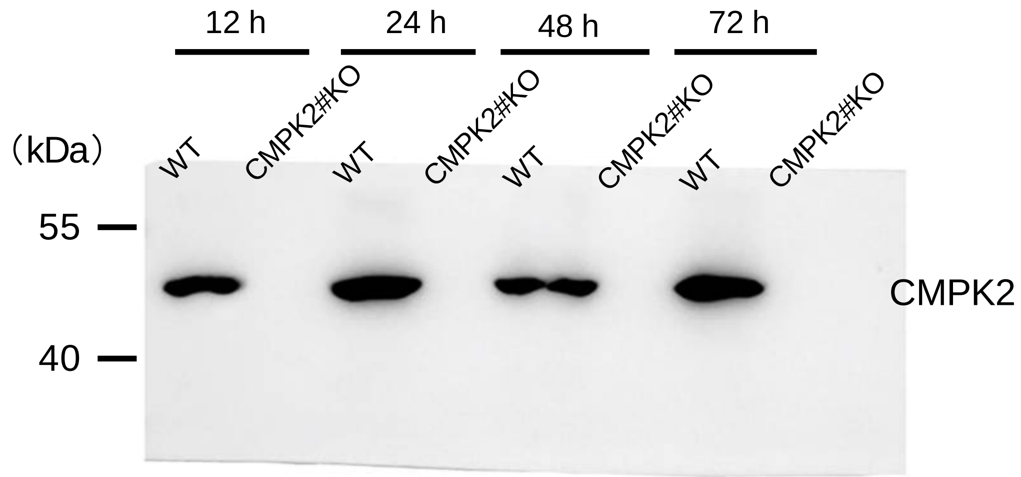

Fig. 4D (β-actin)

β-actin, western blot, anti-β-actin antibody, chemiluminescence, Tanon 5200

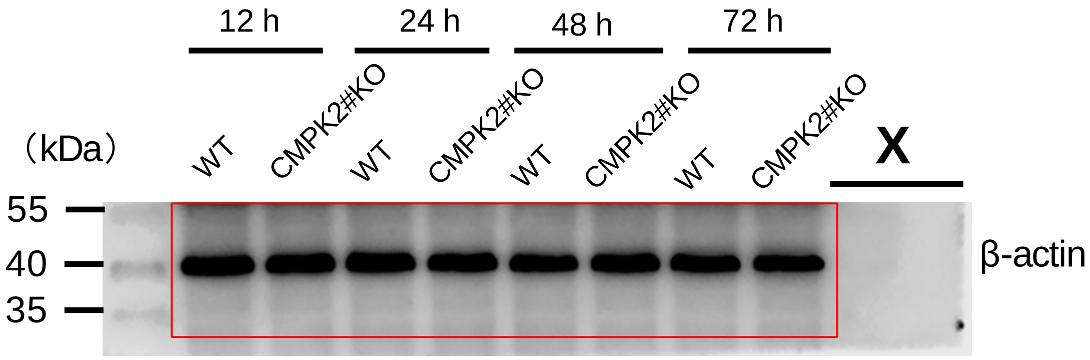

Fig. 4E (PEDV)

PEDV *N*, western blot, anti-N protein antibody, chemiluminescence, Tanon 5200

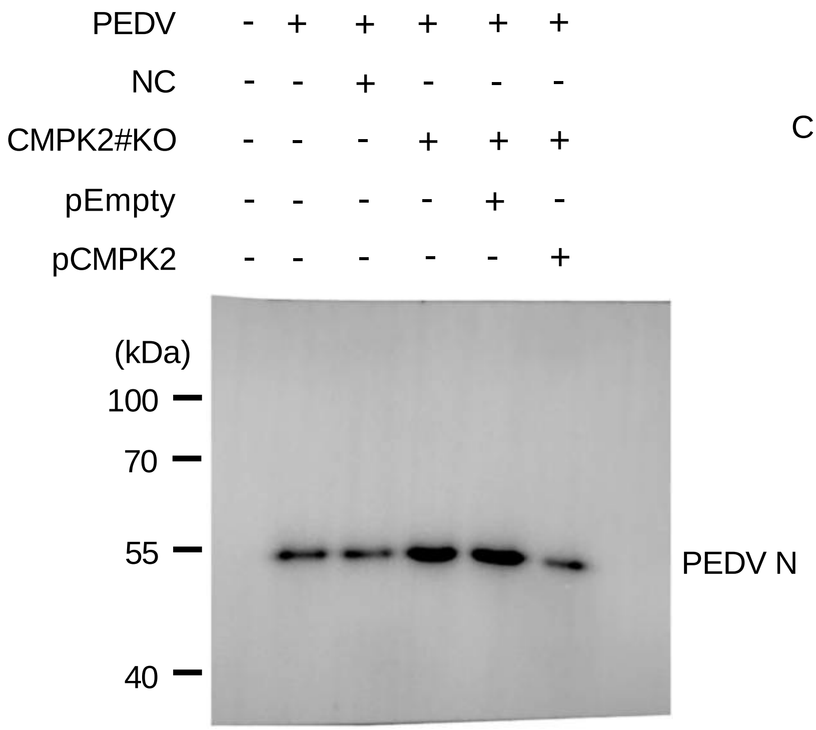

Fig. 4E (CMPK2)

CMPK2, western blot, anti-CMPK2 antibody, chemiluminescence, Tanon 5200

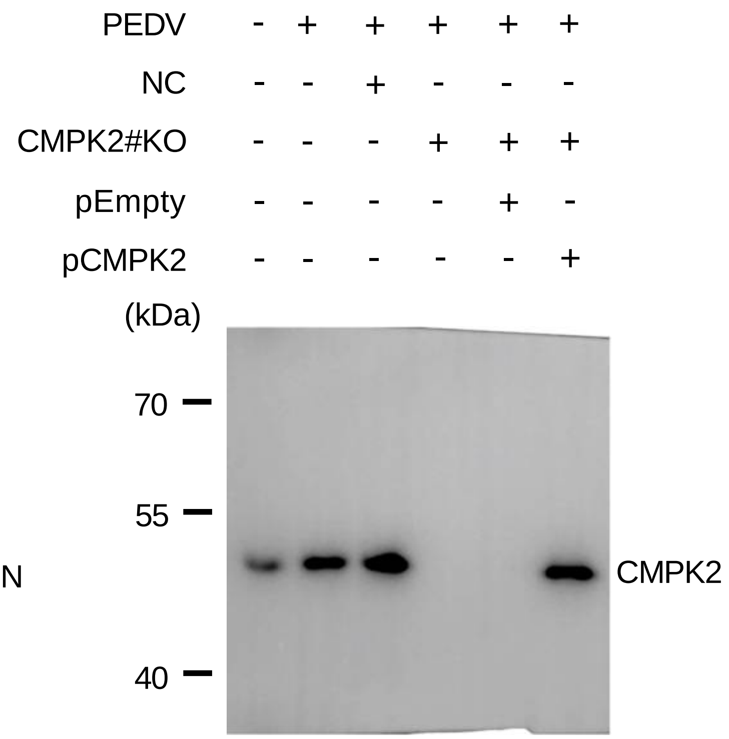

Fig. 4E (β-actin)

β-actin, western blot, anti-β-actin antibody, chemiluminescence, Tanon 5200

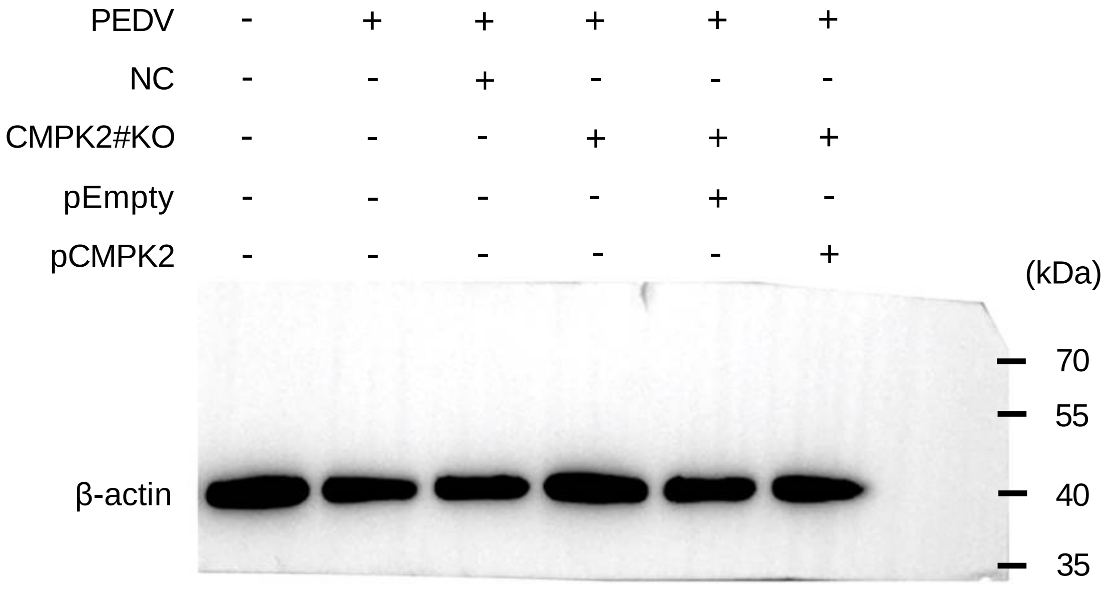

S9B Fig

PEDV *N*, CMPK2 and β-actin, western blot, anti-N protein, CMPK2 and β-actin antibody, chemiluminescence, Tanon 5200

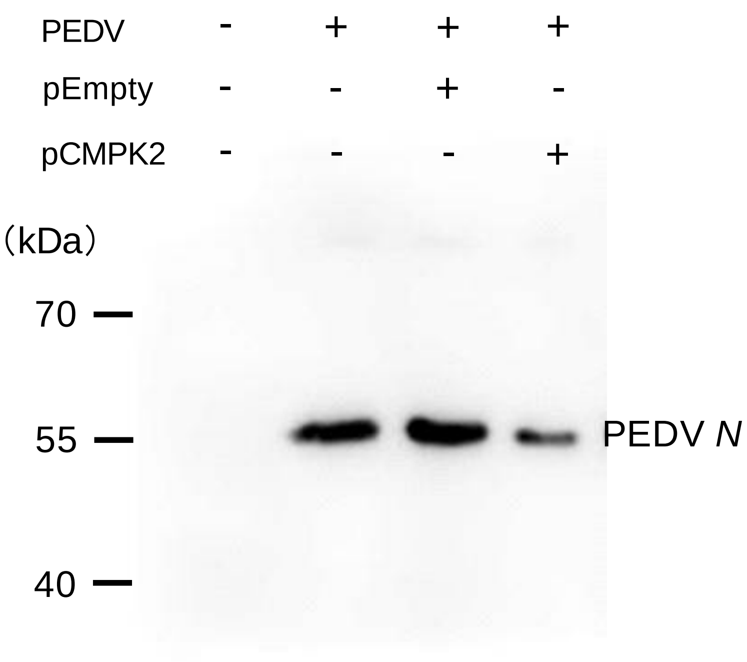

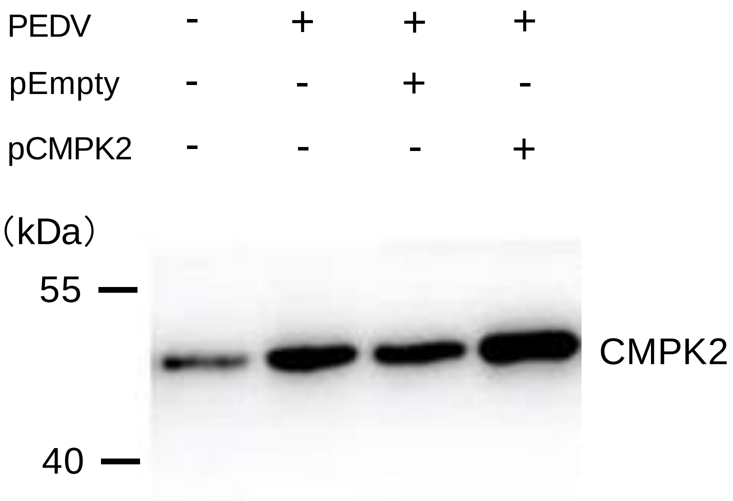

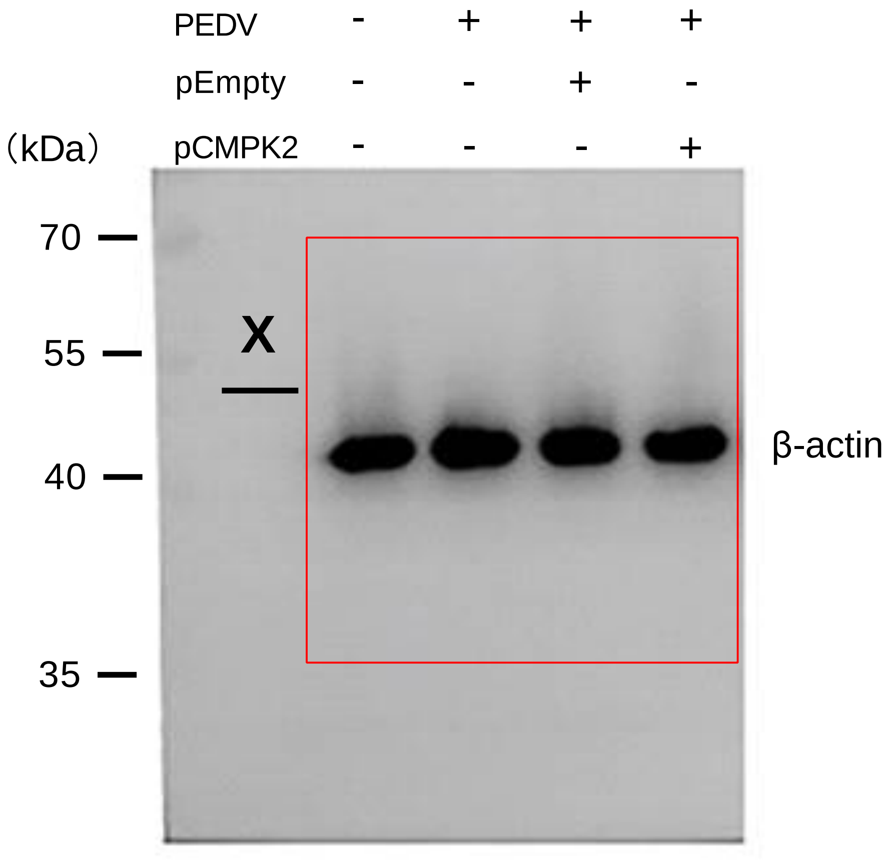

S9D Fig

CMPK2 and β-actin, western blot, CMPK2 and β-actin antibody, chemiluminescence, Tanon 5200

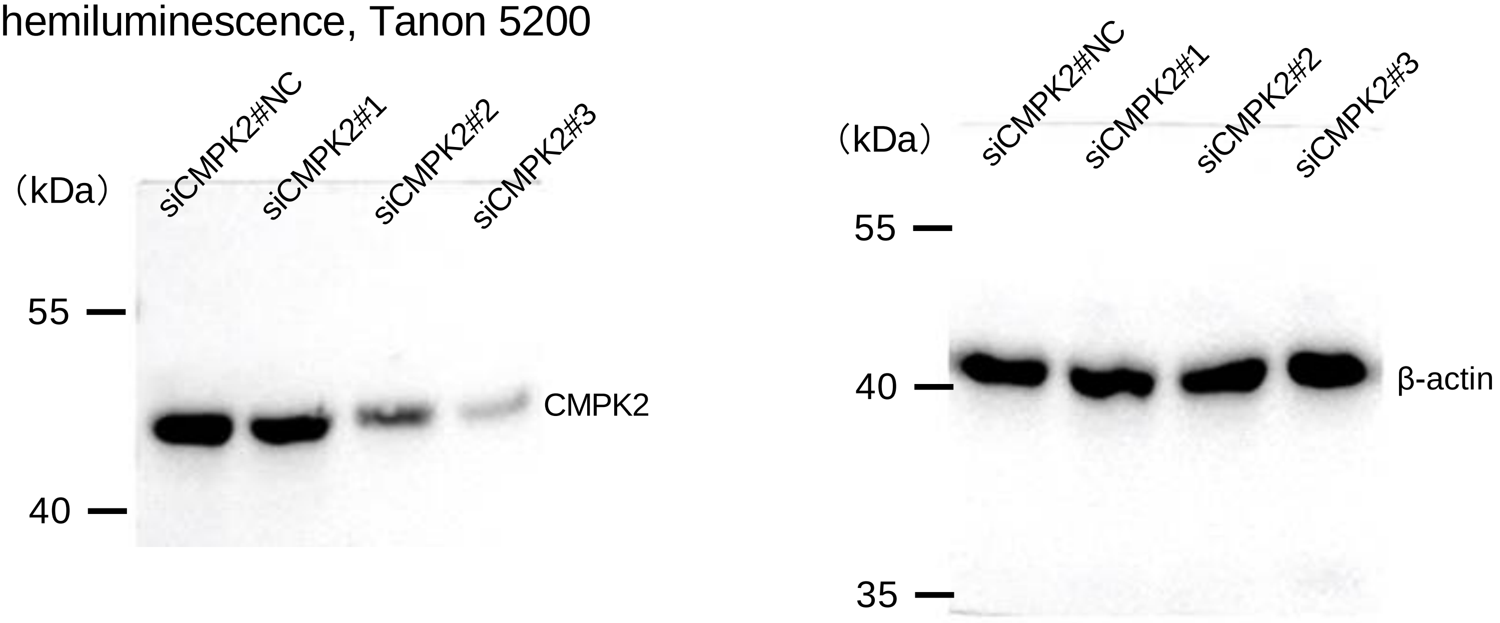

S9E Fig

PEDV *N*, CMPK2 and β-actin, western blot, anti-N protein, CMPK2 and β-actin antibody, chemiluminescence, Tanon 5200

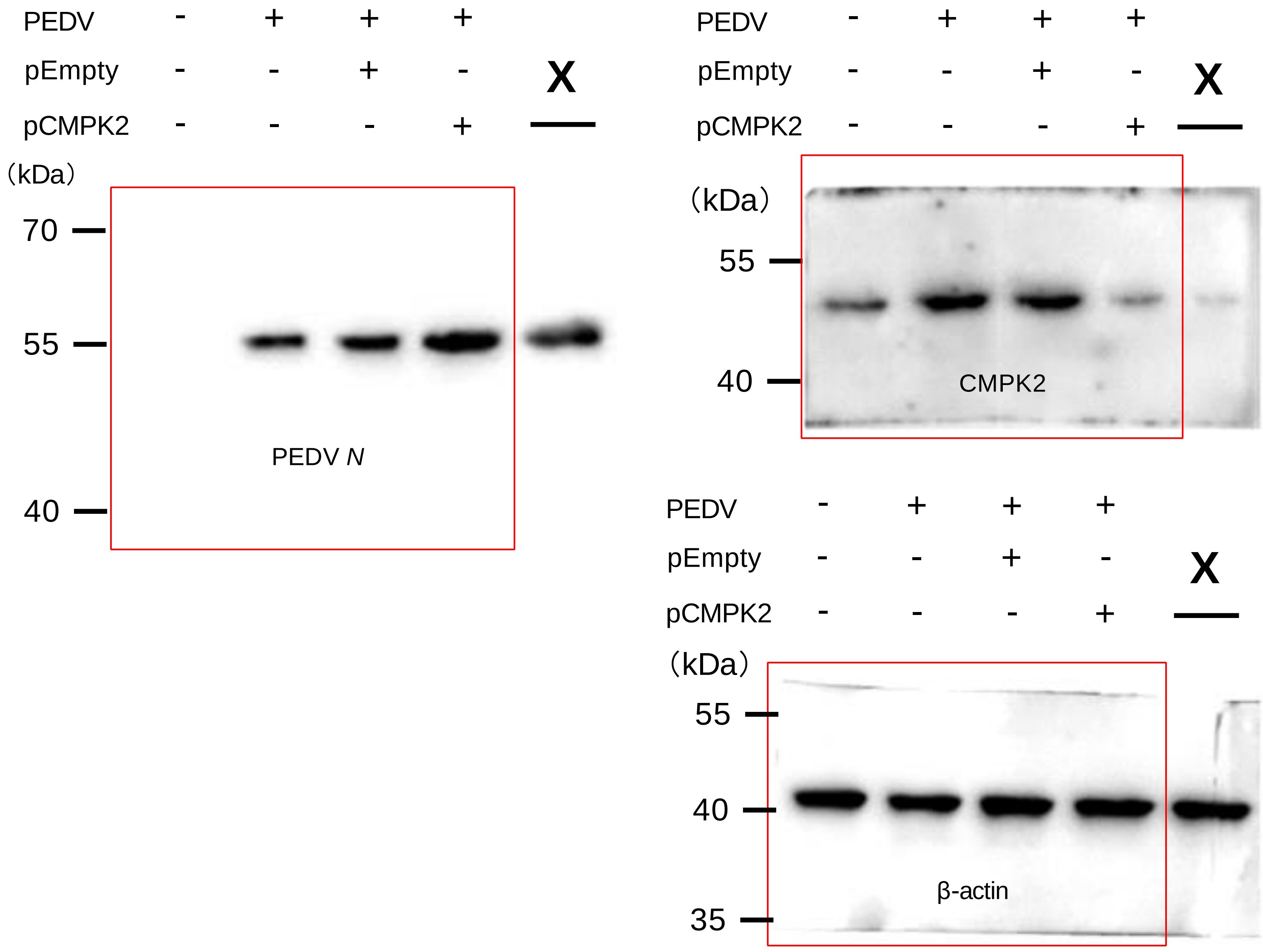

S10D Fig (Top and left)

PEDV *N*, western blot, anti-N protein antibody, chemiluminescence, Tanon 5200

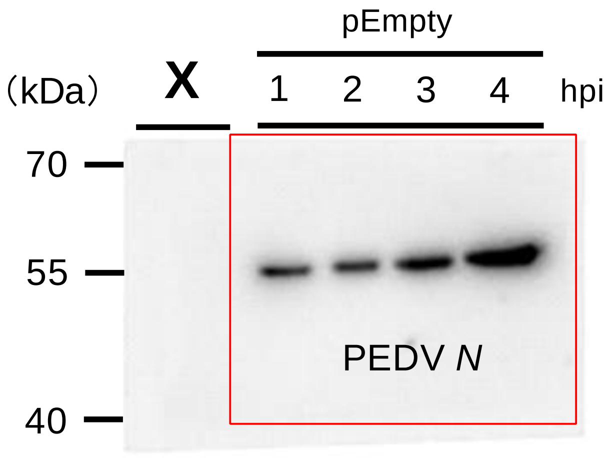

S10D Fig (Top and right)

PEDV *N*, western blot, anti-N protein antibody, chemiluminescence, Tanon 5200

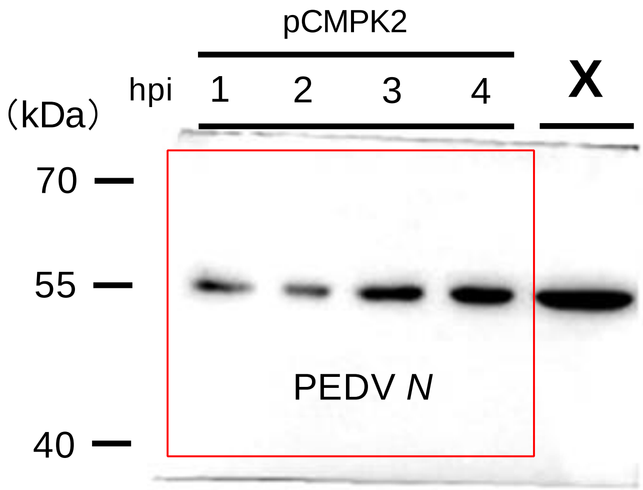

S10D Fig (Bottom and left)

β-actin, western blot, anti-β-actin antibody, chemiluminescence, Tanon 5200

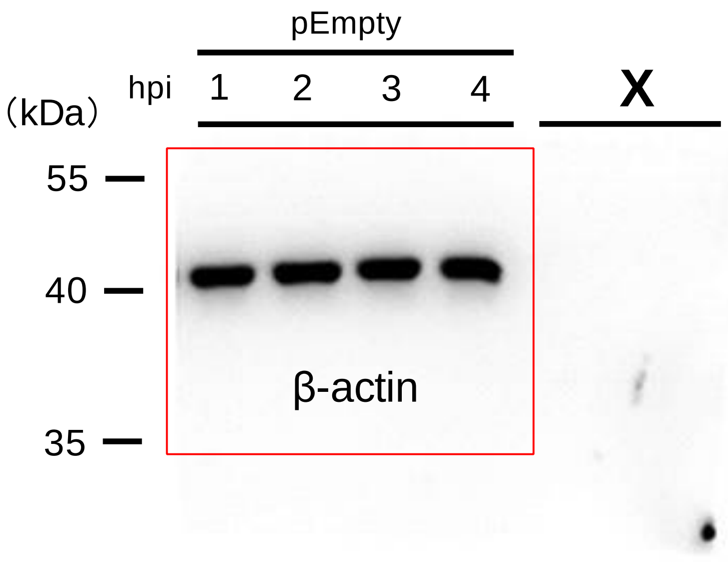

S10D Fig (Bottom and right)

β-actin, western blot, anti-β-actin antibody, chemiluminescence, Tanon 5200

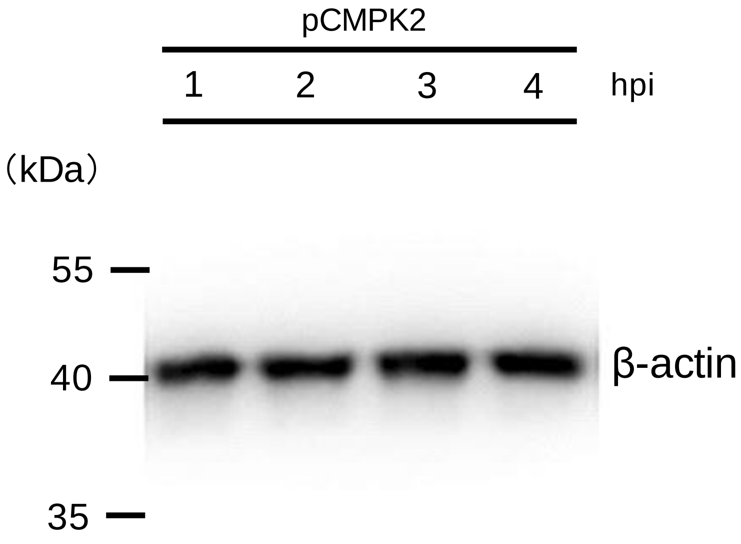

Fig. 5A (PEDV *N*)

PEDV *N*, western blot, anti-N protein antibody, chemiluminescence, Tanon 5200

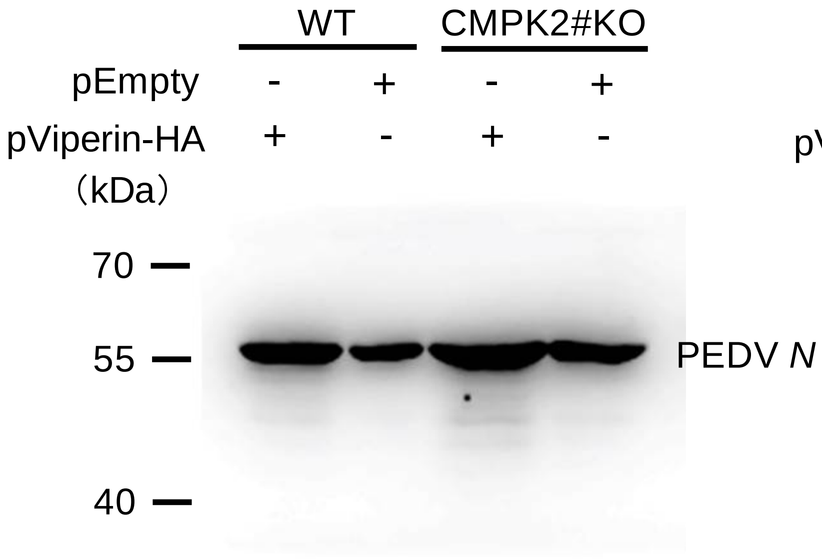

Fig. 5A (Viperin)

Viperin, western blot, anti-Viperin antibody, chemiluminescence, Tanon 5200

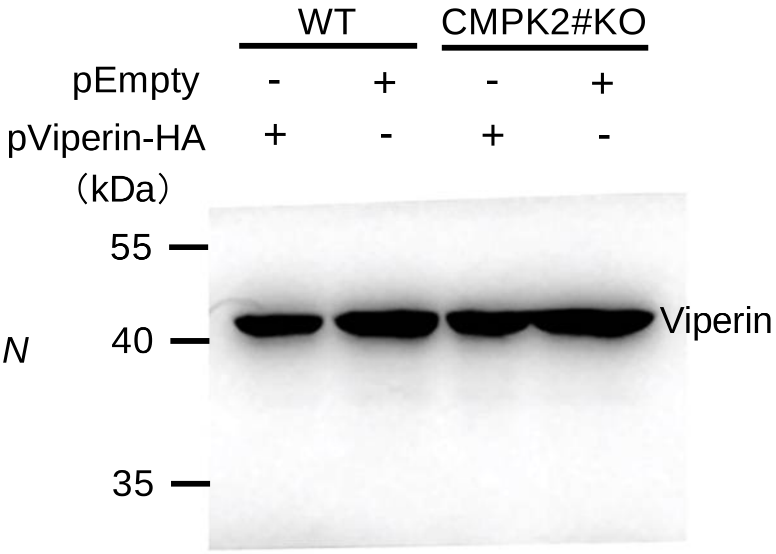

Fig. 5A (CMPK2)

CMPK2, western blot, anti-CMPK2 antibody, chemiluminescence, Tanon 5200

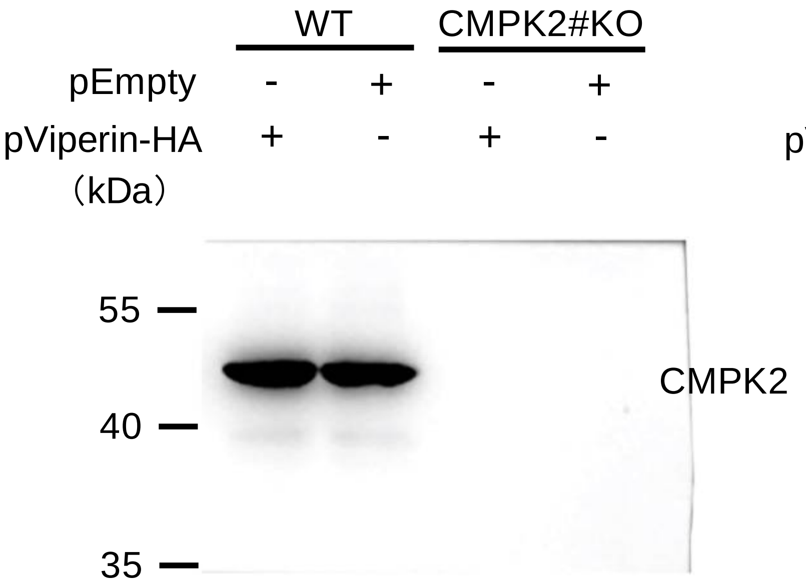

Fig. 5A (β-actin)

β-actin, western blot, anti-β-actin antibody, chemiluminescence, Tanon 5200

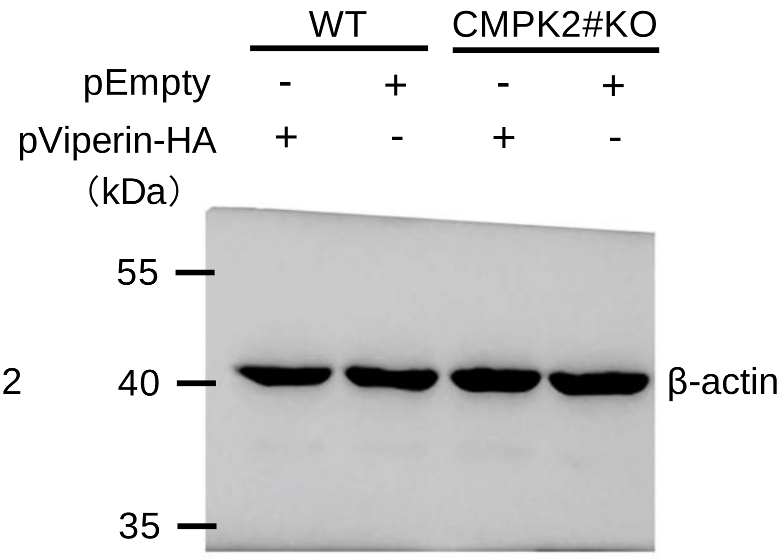

Fig. 5B (PEDV *N*)

PEDV *N*, western blot, anti-N protein antibody, chemiluminescence, Tanon 5200

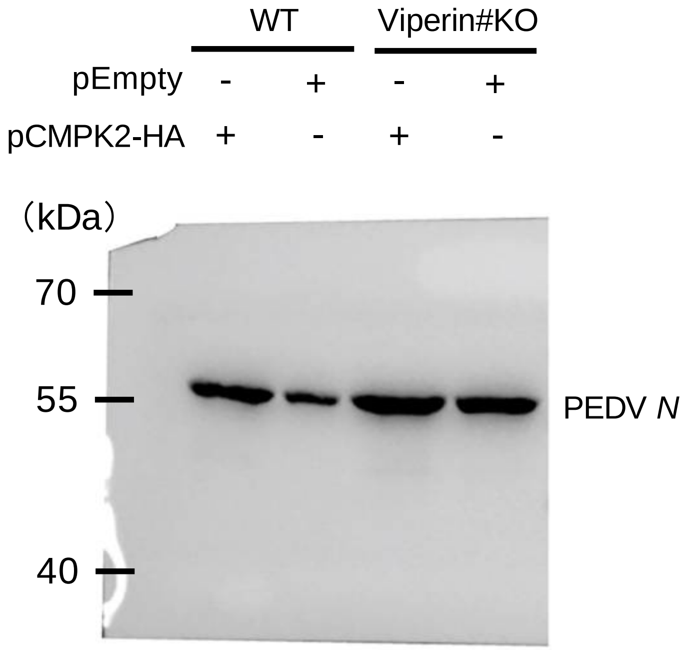

Fig. 5B (CMPK2)

CMPK2, western blot, anti-CMPK2 antibody, chemiluminescence, Tanon 5200

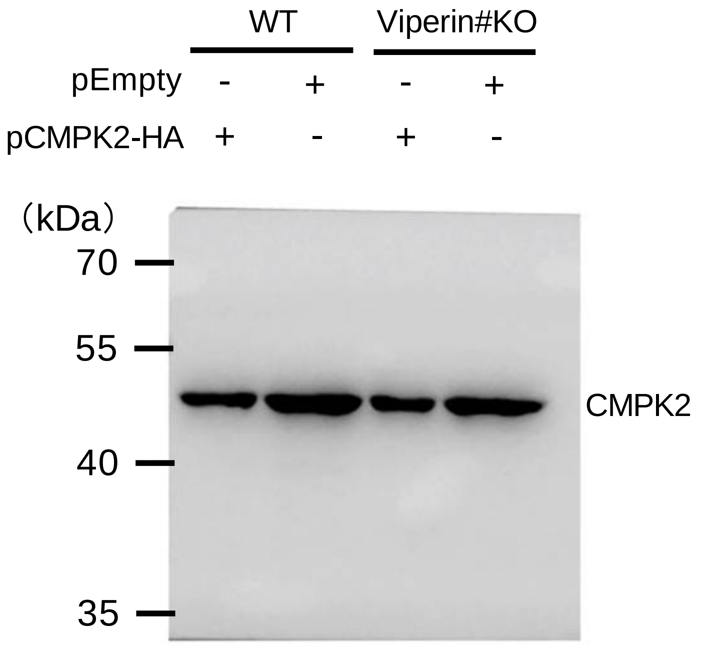

Fig. 5B (Viperin)

Viperin, western blot, anti-Viperin antibody, chemiluminescence, Tanon 5200

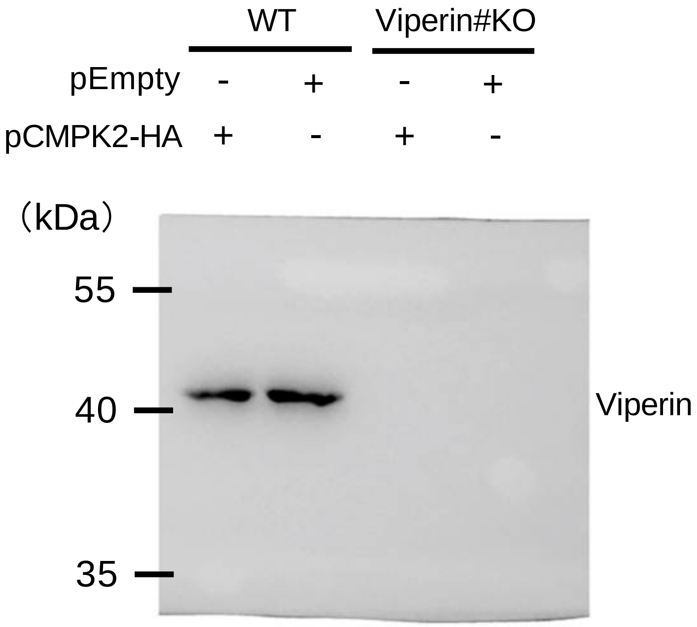

Fig. 5B (β-actin)

β-actin, western blot, anti-β-actin antibody, chemiluminescence, Tanon 5200

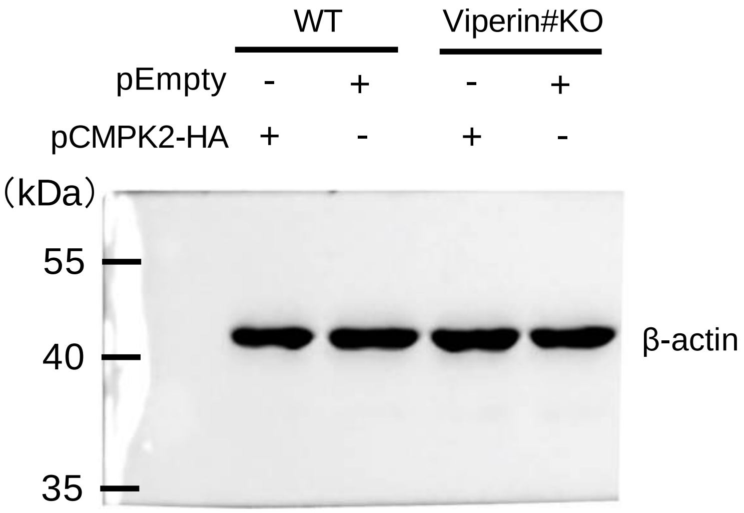

Fig. 5C (PEDV *N*)

PEDV *N*, western blot, anti-N protein antibody, chemiluminescence, Tanon 5200

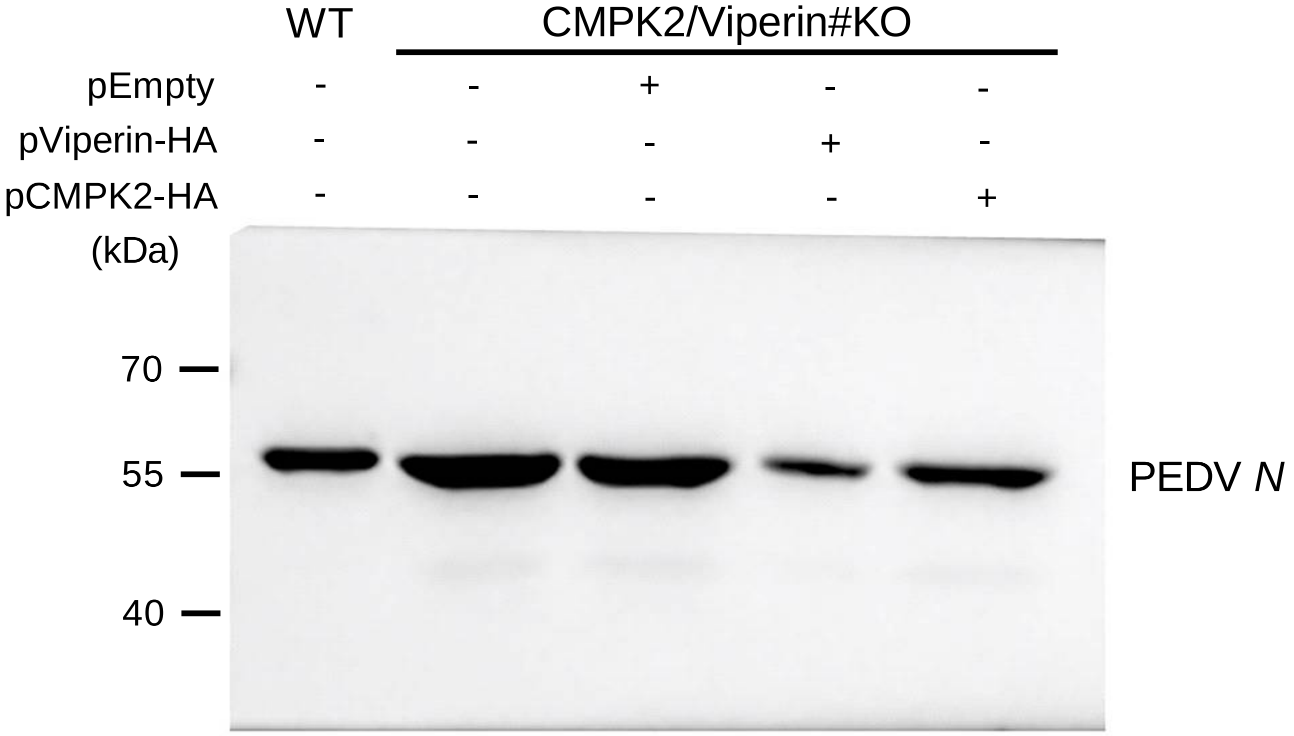

Fig. 5C (CMPK2 and Viperin)

CMPK2 and Viperin, western blot, anti-CMPK2 and Viperin antibody, chemiluminescence, Tanon 5200

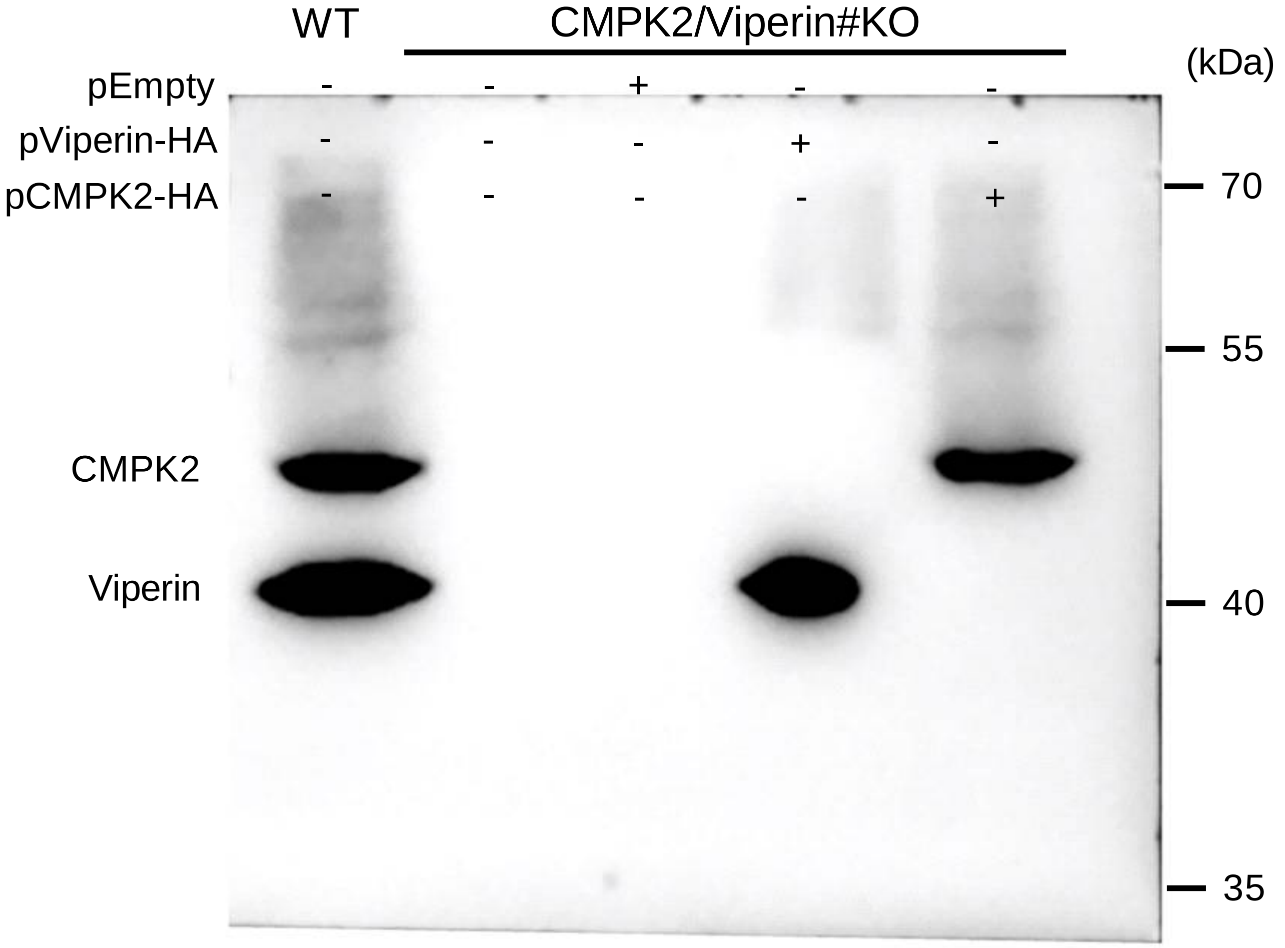

Fig. 5C (β-actin)

β-actin, western blot, anti-β-actin antibody, chemiluminescence, Tanon 5200

	WT	CMPK2/Viperin#KO			
pEmpty	-	-	+	-	-
pViperin-HA	-	-	-	+	-
pCMPK2-HA	-	-	-	-	+

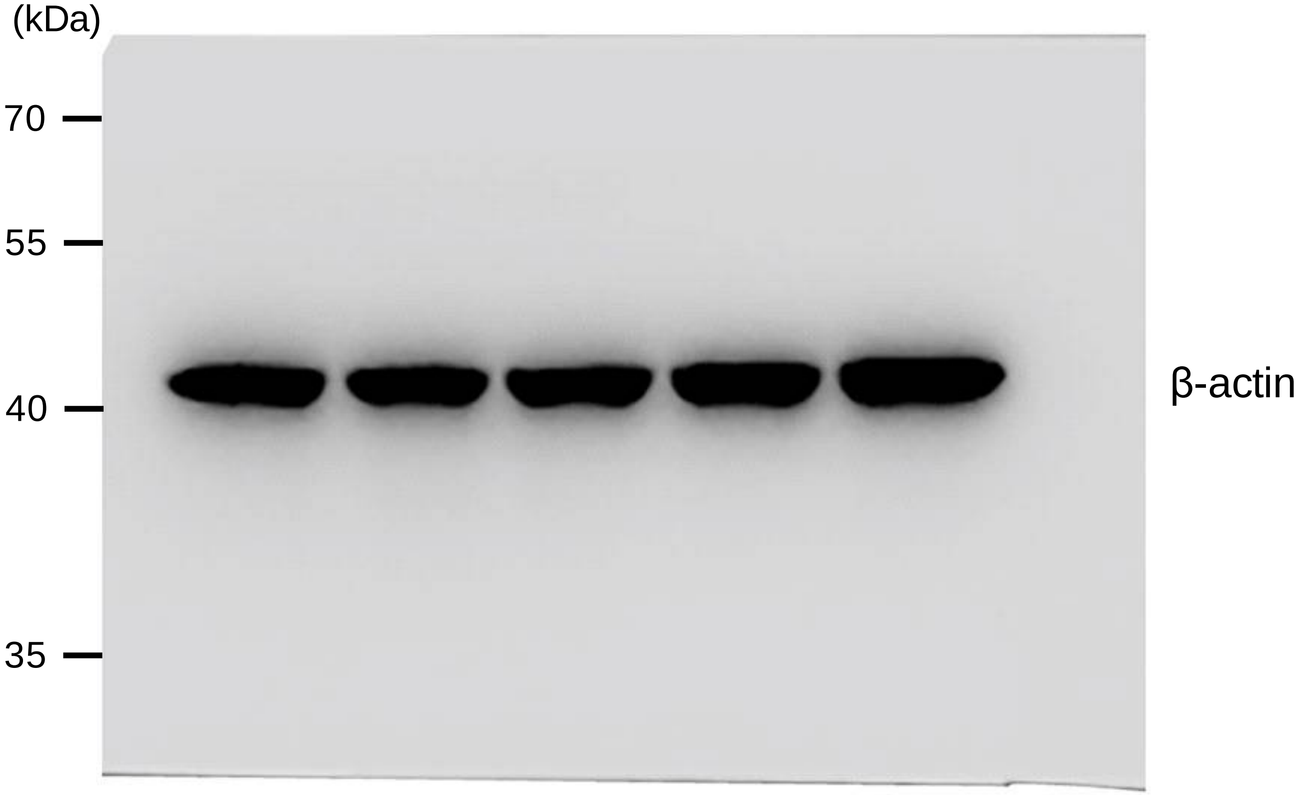

Fig. 5F (PEDV *N*)

PEDV *N*, western blot, anti-N protein antibody, chemiluminescence, Tanon 5200

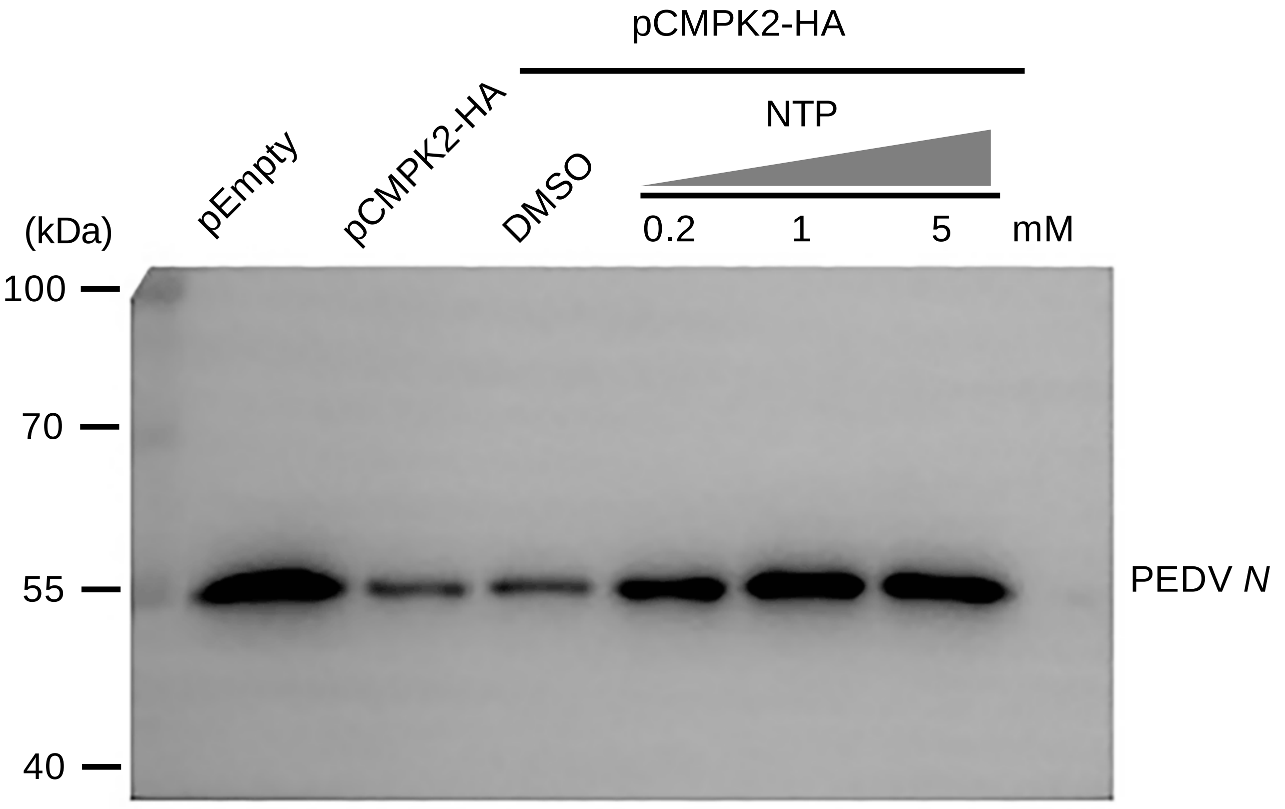

Fig. 5F (CMPK2)

CMPK2-HA, western blot, anti-HA antibody, chemiluminescence, Tanon 5200

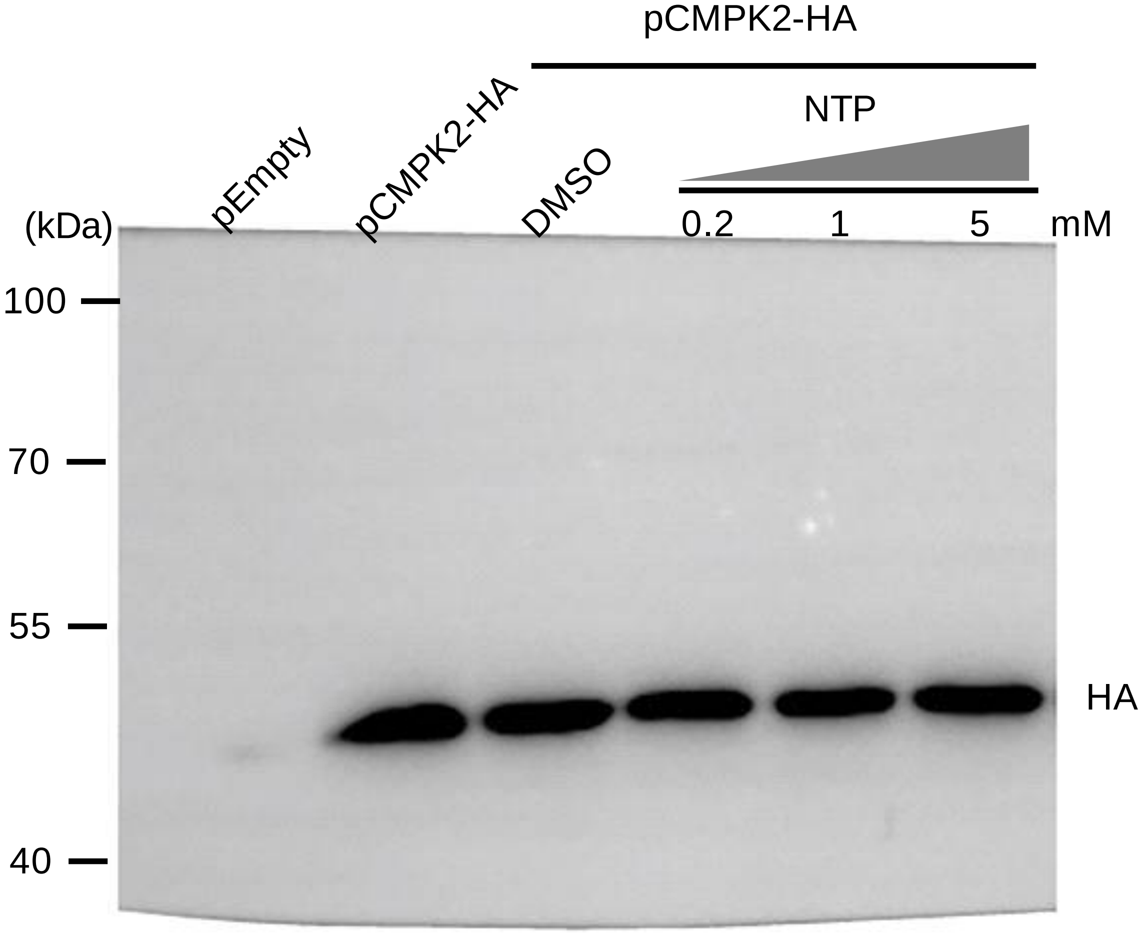

Fig. 5F (β-actin)

β-actin, western blot, anti-β-actin antibody, chemiluminescence, Tanon 5200

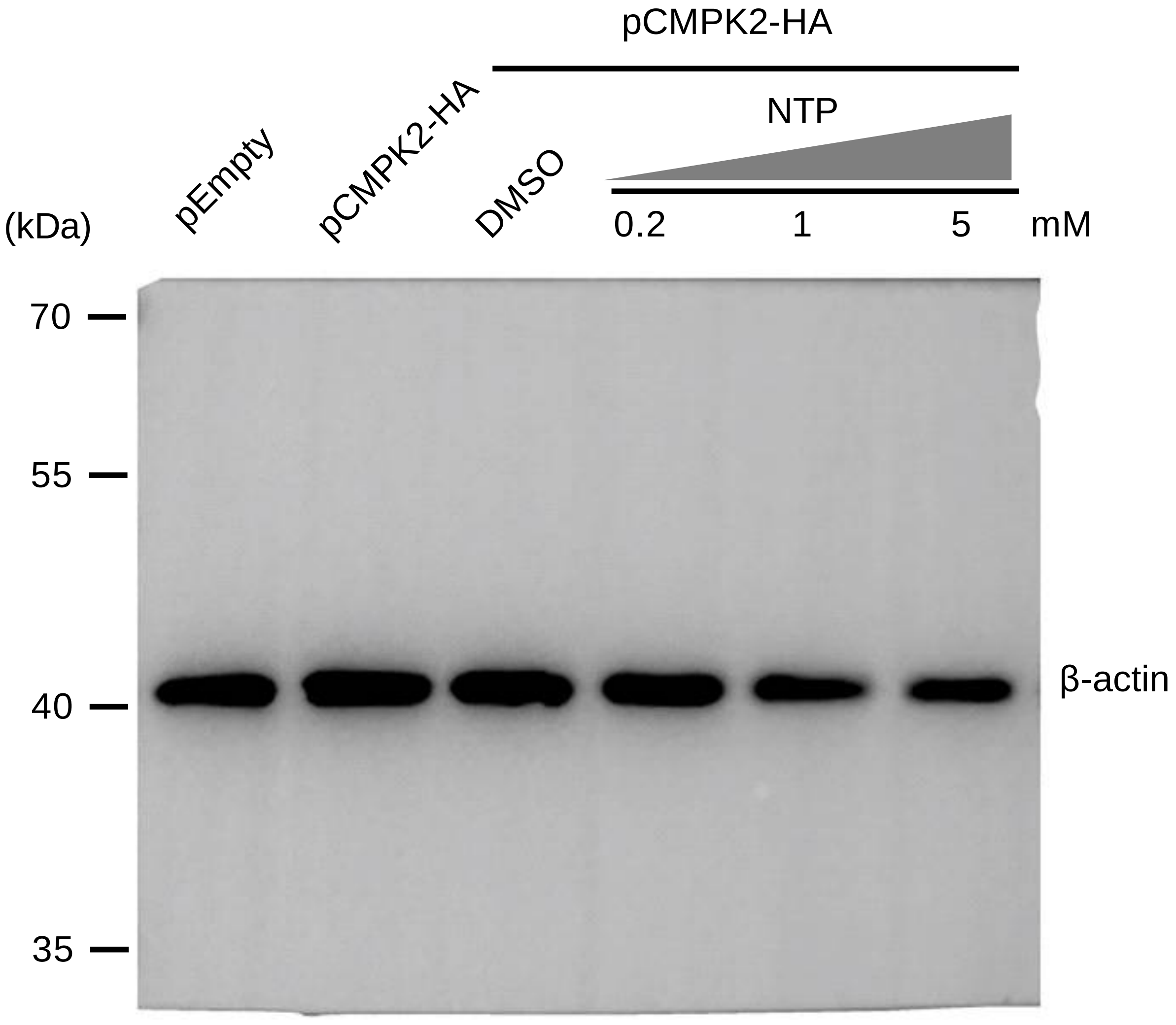

S11A Fig (Viperin)

Viperin, western blot, anti-Viperin antibody, chemiluminescence, Tanon 5200

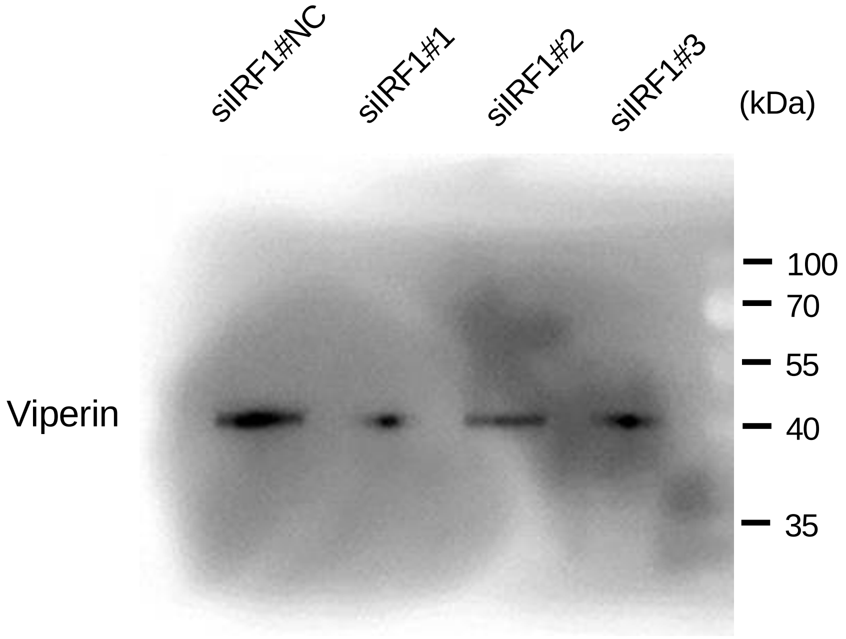

S11A Fig (β-actin)

β-actin, western blot, anti-β-actin antibody, chemiluminescence, Tanon 5200

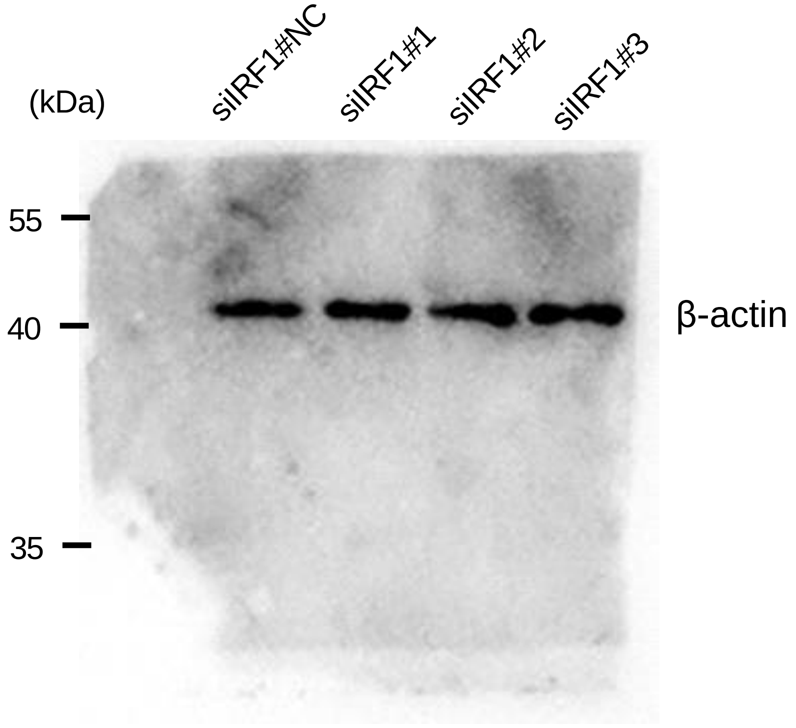

S11B Fig (PEDV *N*)

PEDV *N*, western blot, anti-N protein antibody, chemiluminescence, Tanon 5200

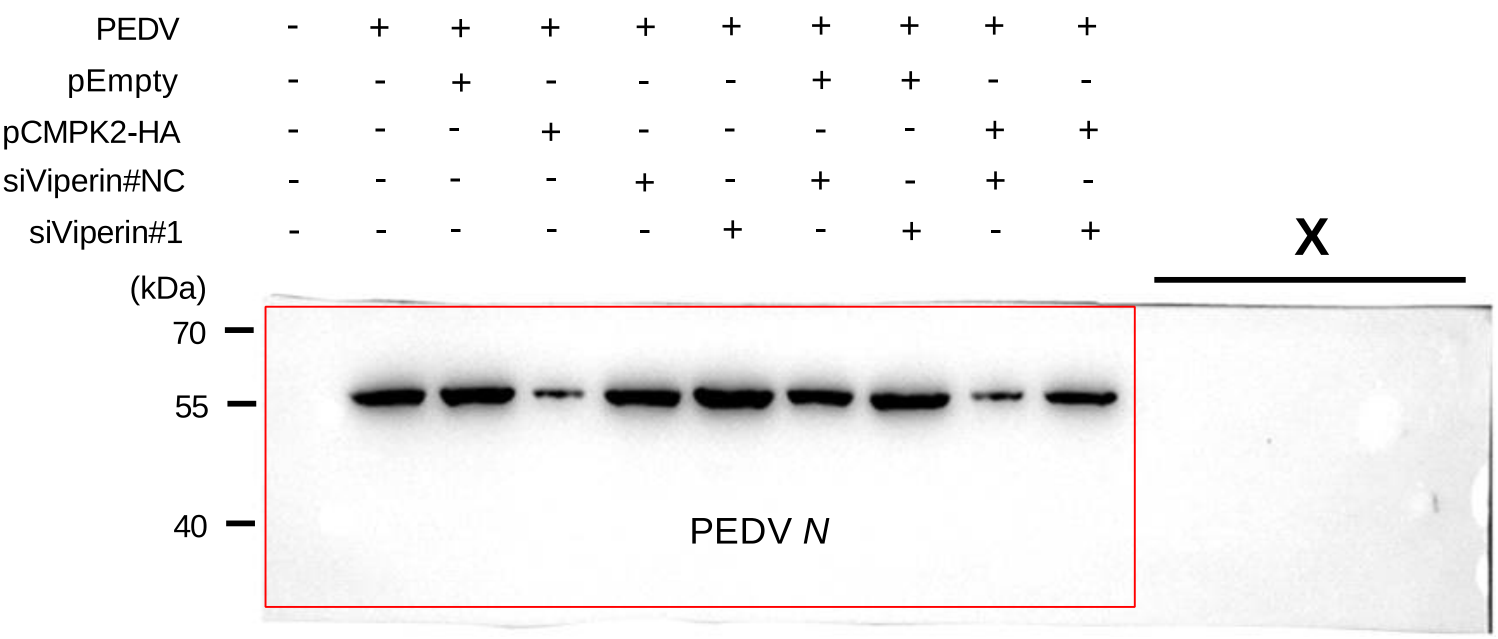

S11B Fig (CMPK2)

CMPK2-HA, western blot, anti-HA antibody, chemiluminescence, Tanon 5200

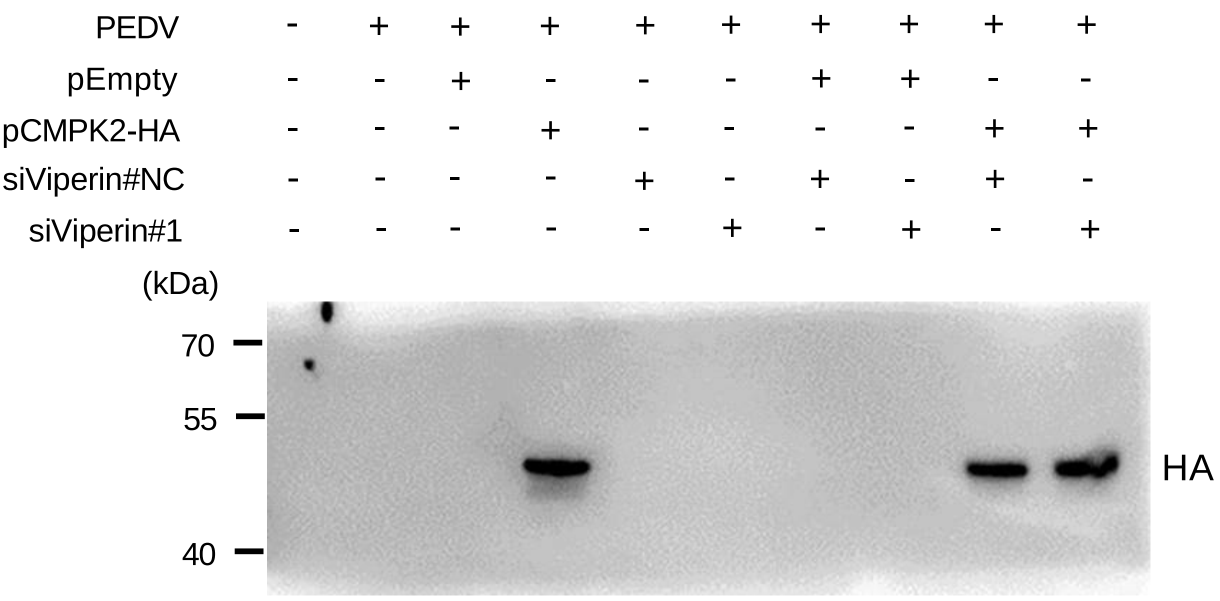

S11B Fig (Viperin)

Viperin, western blot, anti-Viperin antibody, chemiluminescence, Tanon 5200

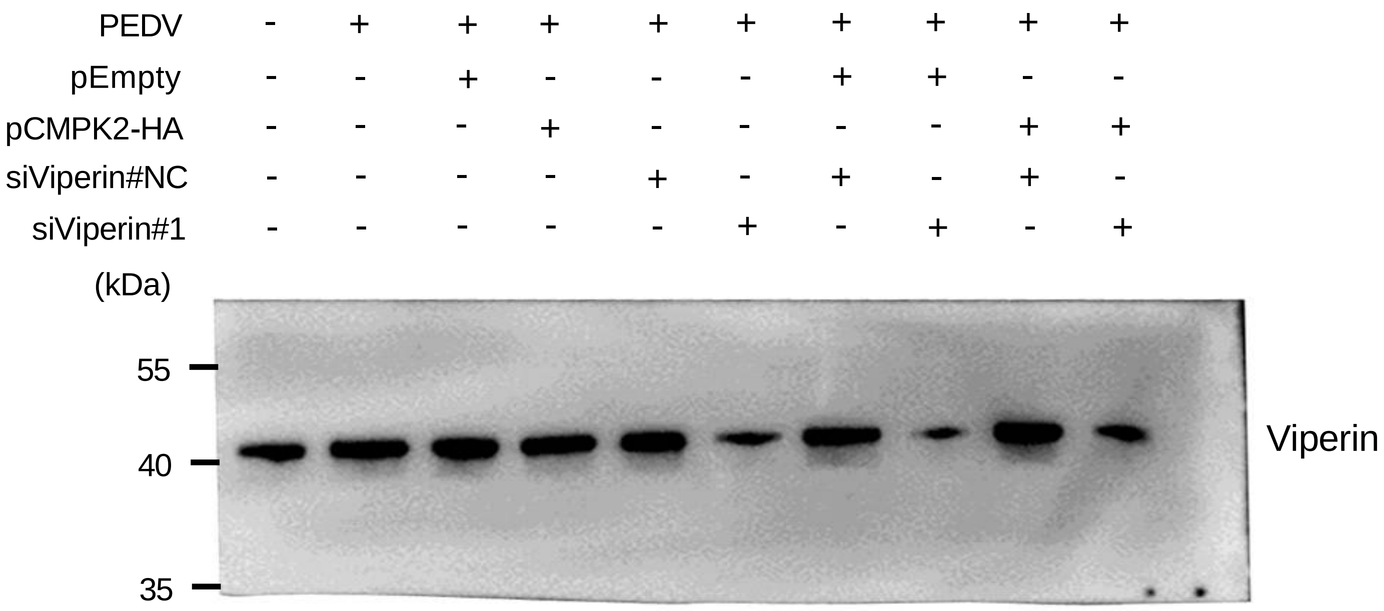

S11B Fig (β-actin)

β-actin, western blot, anti-β-actin antibody, chemiluminescence, Tanon 5200

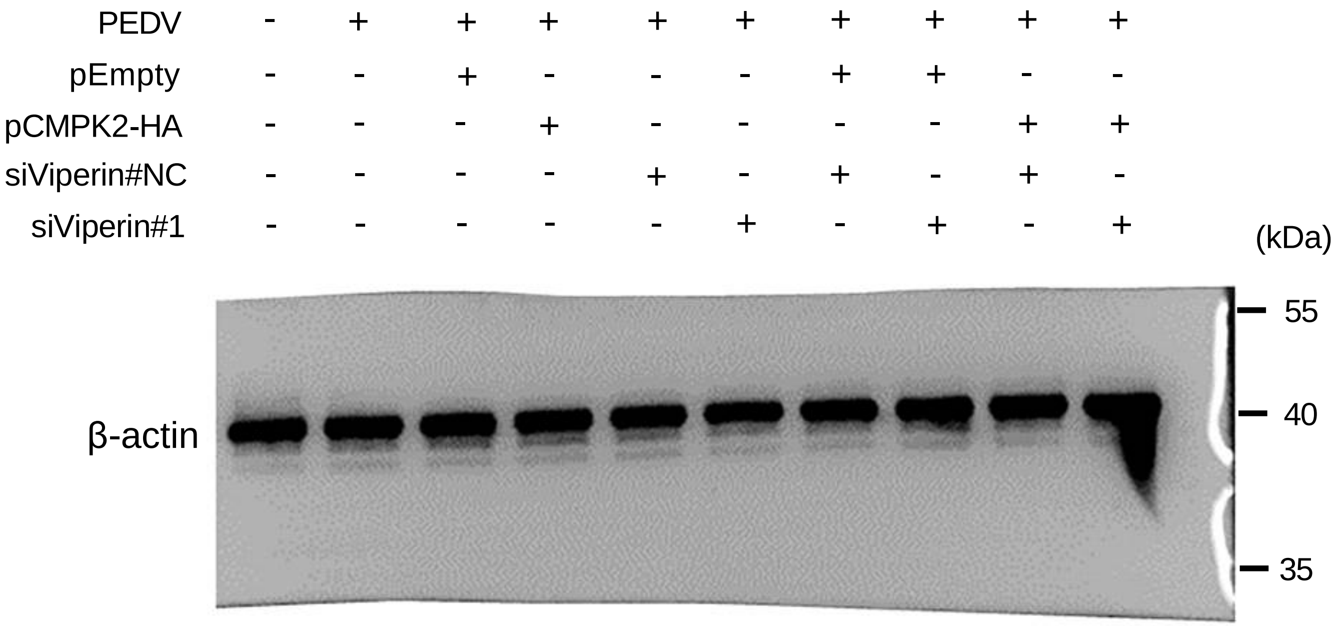

Fig. 6B (PEDV *N* and β-actin)

PEDV *N* and β-actin, western blot, anti-N protein and β-actin antibody, chemiluminescence, Tanon 5200

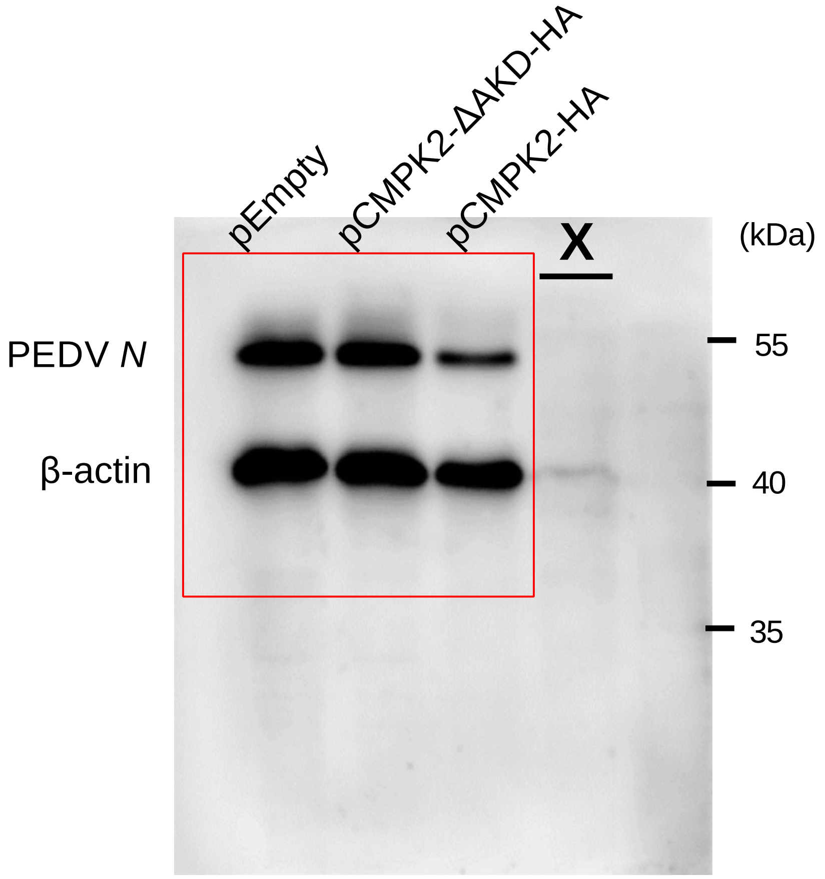

Fig. 6B (HA)

HA, western blot, anti-HA antibody, chemiluminescence, Tanon 5200

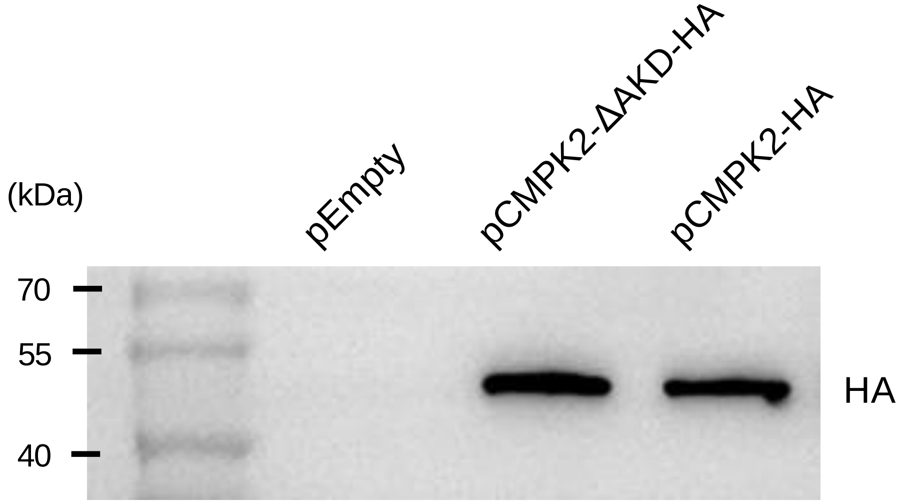

Fig. 6F (PEDV *N*)

PEDV *N*, western blot, anti-N protein antibody, chemiluminescence, Tanon 5200

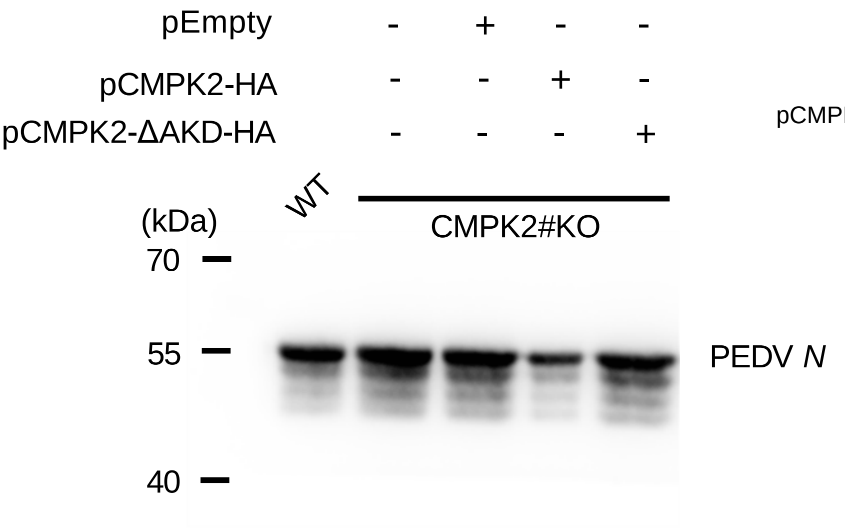

Fig. 6F (CMPK2)

CMPK2, western blot, anti-CMPK2 antibody, chemiluminescence, Tanon 5200

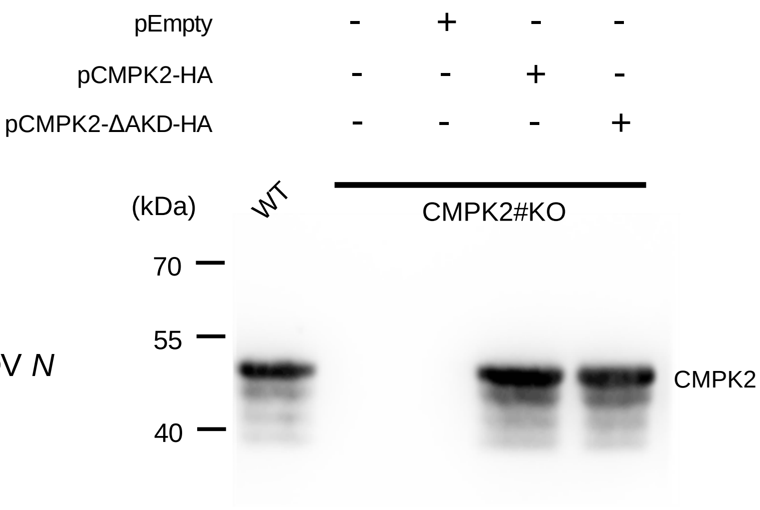

Fig. 6F (HA)

HA, western blot, anti-HA antibody, chemiluminescence, Tanon 5200

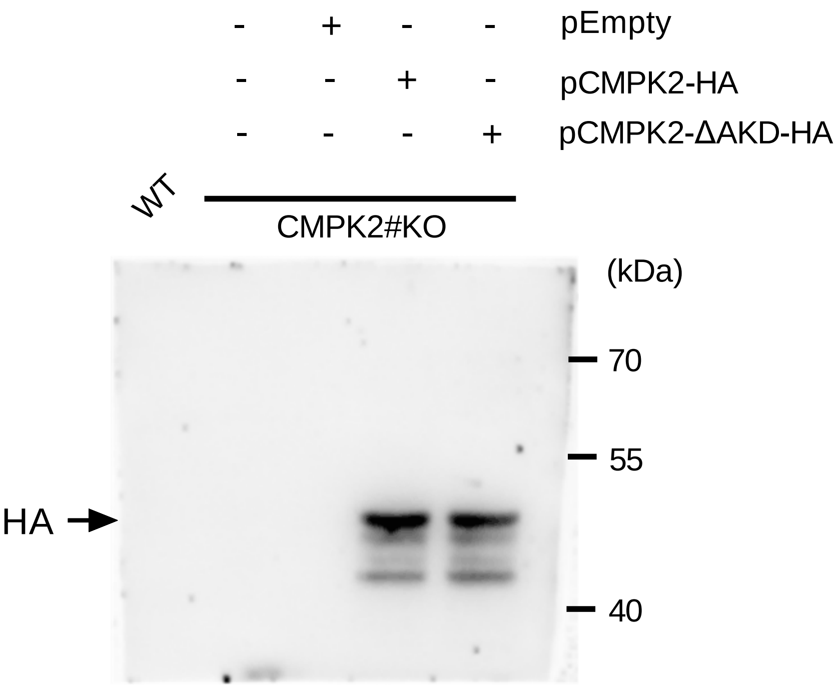

Fig. 6F (β-actin)

β-actin, western blot, anti-β-actin antibody, chemiluminescence, Tanon 5200

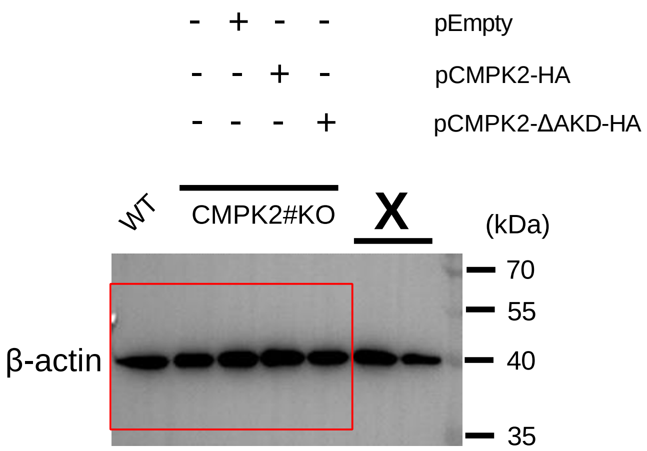

S12C Fig (PEDV *N*)

PEDV *N*, western blot, anti-N protein antibody, chemiluminescence, Tanon 5200

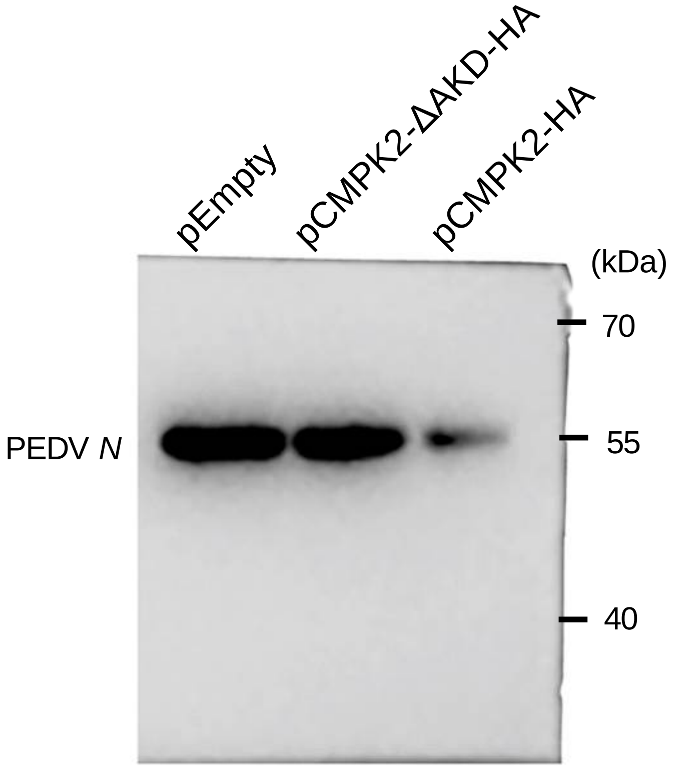

S12C Fig (HA)

HA, western blot, anti-HA antibody, chemiluminescence, Tanon 5200

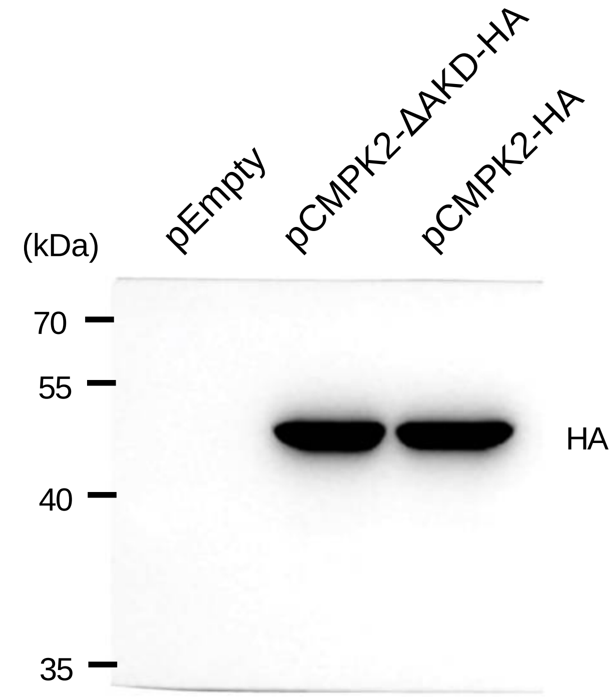

S12C Fig (β-actin)

β-actin, western blot, anti-β-actin antibody, chemiluminescence, Tanon 5200

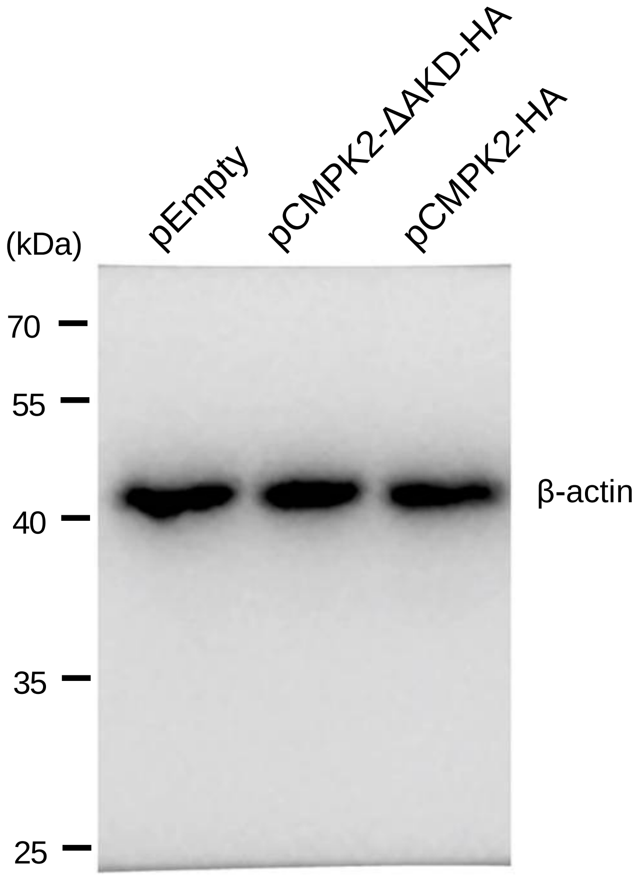

Fig. 8A (IBV *N*)

IBV *N*, western blot, anti-IBV N protein antibody, chemiluminescence, Tanon 5200

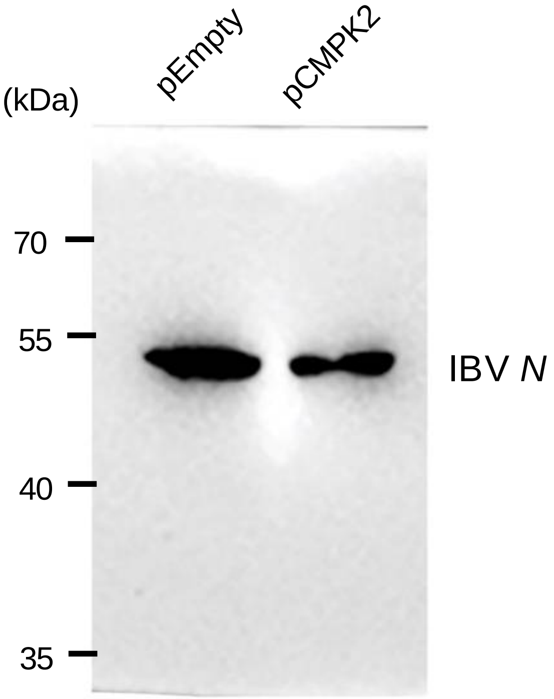

Fig. 8A (CMPK2)

CMPK2, western blot, anti-CMPK2 antibody, chemiluminescence, Tanon 5200

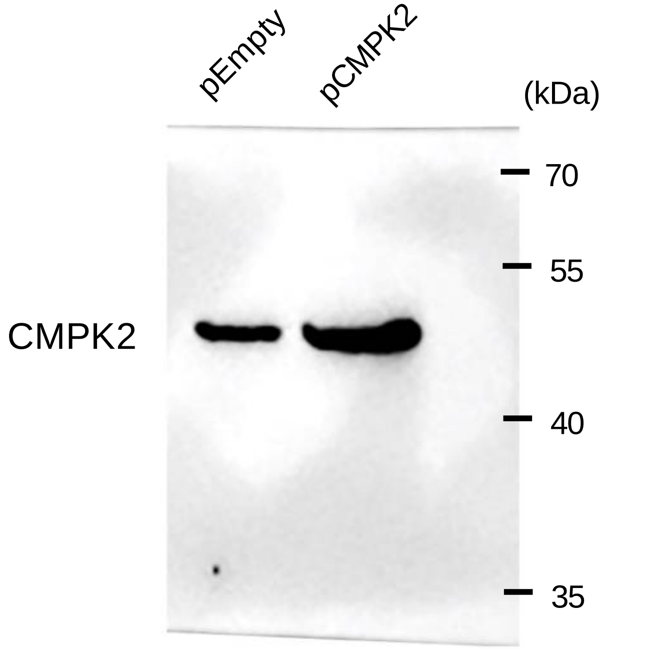

Fig. 8A (β-actin)

β-actin, western blot, anti-β-actin antibody, chemiluminescence, Tanon 5200

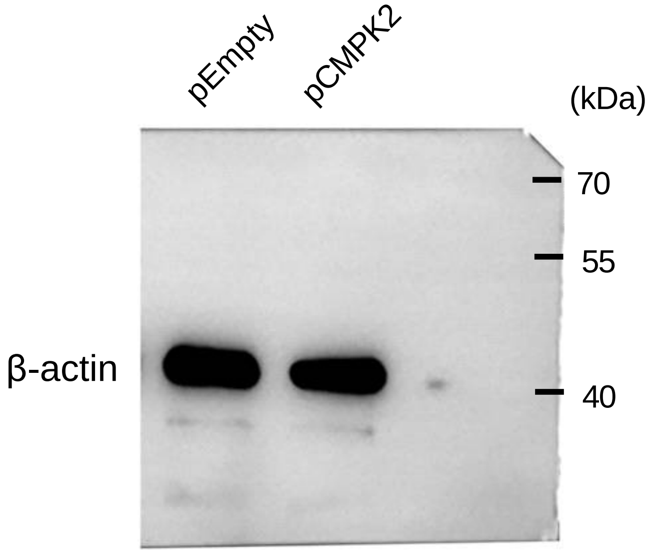

Fig. 8D (PDCoV)

PDCoV *N*, western blot, anti-PDCoV N protein antibody, chemiluminescence, Tanon 5200

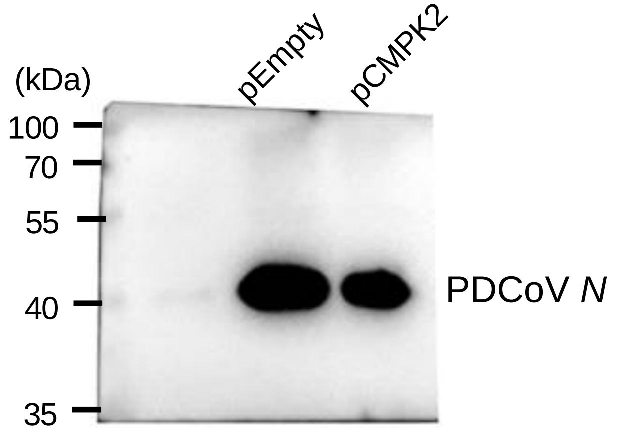

Fig. 8D (CMPK2)

CMPK2, western blot, anti-CMPK2 protein antibody, chemiluminescence, Tanon 5200

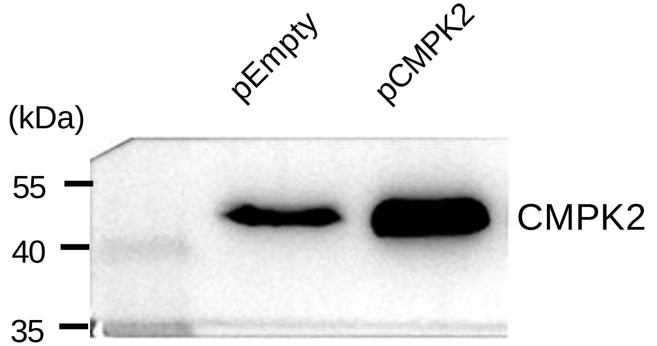

Fig. 8D (β-actin)

β-actin, western blot, anti-β-actin antibody, chemiluminescence, Tanon 5200

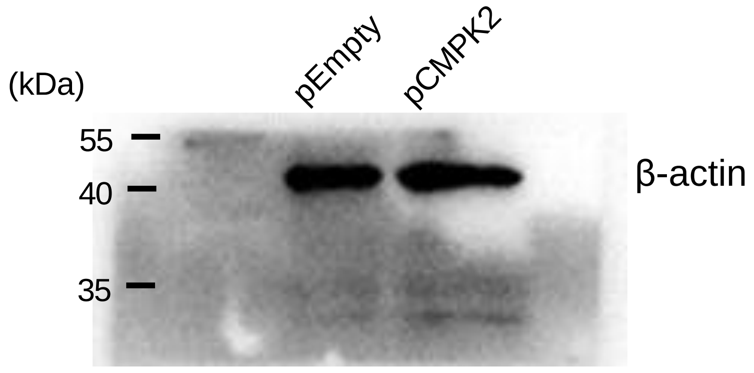

Supplement: S1 Raw images — (PDF) [file pbio.3002039.s018.pdf]
